# On-demand cell-autonomous gene therapy for brain circuit disorders

**DOI:** 10.1126/science.abq6656

**Published:** 2022-11-03

**Authors:** Yichen Qiu, Nathanael O’Neill, Benito Maffei, Clara Zourray, Amanda Almacellas-Barbanoj, Jenna C. Carpenter, Steffan P. Jones, Marco Leite, Thomas J. Turner, Francisco C. Moreira, Albert Snowball, Tawfeeq Shekh-Ahmad, Vincent Magloire, Serena Barral, Manju A. Kurian, Matthew C. Walker, Stephanie Schorge, Dimitri M. Kullmann, Gabriele Lignani

**Affiliations:** 1 Department of Clinical and Experimental Epilepsy, UCL Queen Square Institute of Neurology, University College London, London, UK; 2 Department of Developmental Neurosciences, Zayed Centre for Research Into Rare Disease in Children, GOS−Institute of Child Health, University College London, London, UK; 3 Department of Neurology, Great Ormond Street Hospital for Children, London, UK; 4 Department of Neuroscience, Physiology and Pharmacology University College London, London, UK

## Abstract

Several neurodevelopmental and neuropsychiatric disorders are characterized by intermittent episodes of pathological activity. Although genetic therapies offer the ability to modulate neuronal excitability, a limiting factor is that they do not discriminate between neurons involved in circuit pathologies and “healthy” surrounding or intermingled neurons. We describe a gene therapy strategy that down-regulates the excitability of overactive neurons in closed loop, which we tested in models of epilepsy. We used an immediate early gene promoter to drive the expression of Kv1.1 potassium channels specifically in hyperactive neurons, and only for as long as they exhibit abnormal activity. Neuronal excitability was reduced by seizure-related activity, leading to a persistent antiepileptic effect without interfering with normal behaviors. Activity-dependent gene therapy is a promising on- demand cell-autonomous treatment for brain circuit disorders.

## Introduction

Many neurodevelopmental and neuropsychiatric circuit disorders are characterized by intermittent episodes of pathological activity (1–3). Pharmacotherapy is focused on reducing the frequency and/or severity of such episodes, but often with limited success. For example, 30% of people with epilepsy are refractory to pharmacological treatment, even with drugs that act in a use-dependent manner on their molecular targets (4). Genetic therapies are promising strategies to treat these pathologies because of their ability to modulate neuronal excitability in a region-specific and cell type-specific manner (5–9). However, current experimental genetic therapies do not discriminate between neurons involved in triggering circuit paroxysms and healthy surrounding or intermingled neurons (10–16). There is a need to develop methods that select and treat only those neurons involved in the generation of crises (17, 18). In the case of epilepsy, subpopulations of neurons exhibit stereotypical patterns of pathological discharges during seizures (19, 20). Targeting these neurons specifically would be an important step toward a rational treatment with minimal side effects.

**Fig. 1:**
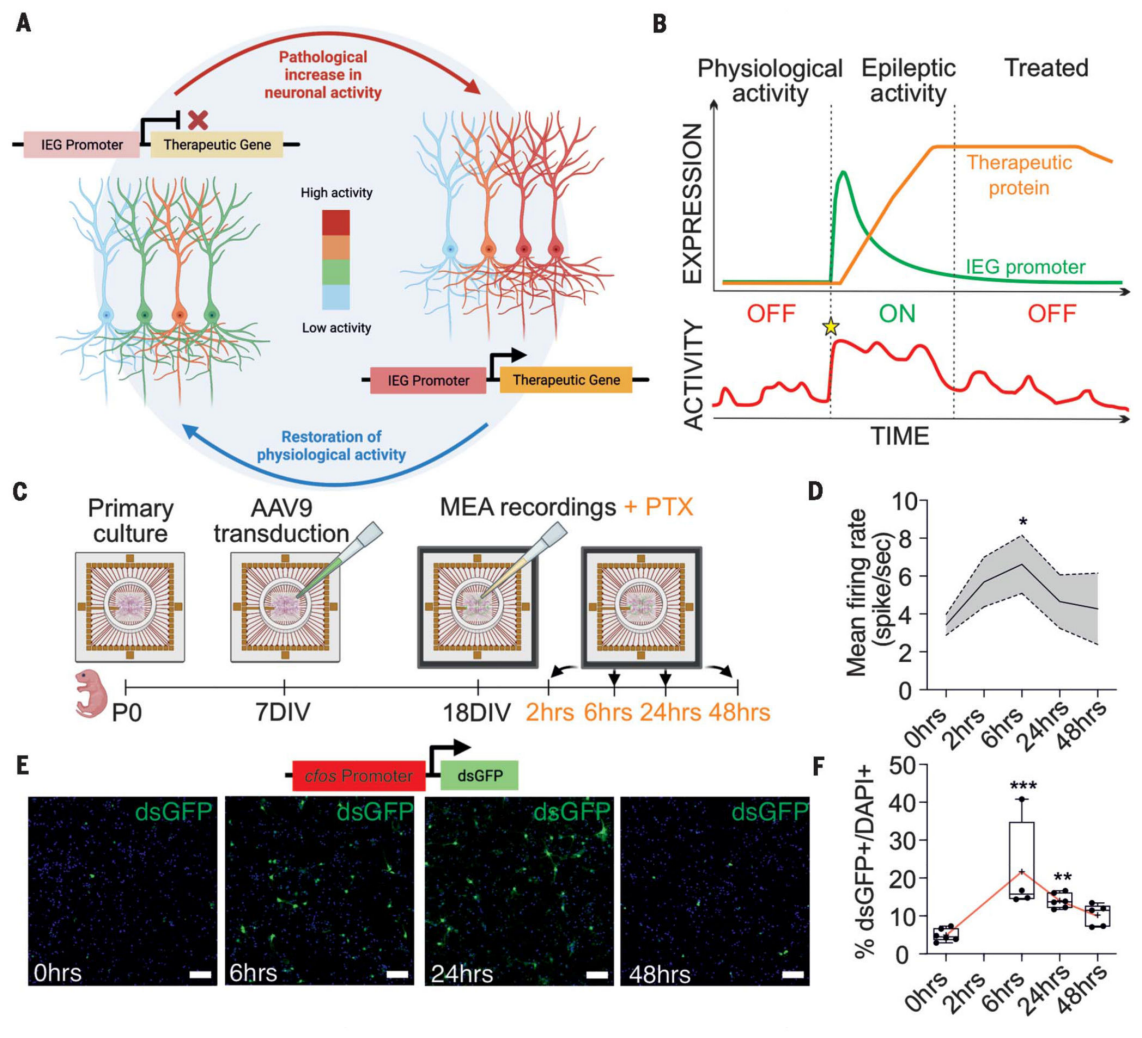
Activity-dependent gene therapy paradigm. (**A**) Cartoon illustrating the activity-dependent strategy. (**B**) Schematic seq uence of activity-dependent promoter and transgene expression. The star indicates the beginning of the pathological event. (**C**) Timeline of MEA recordings from primary cultures. (**D**) Network mean firing rate after disinhibition with PTX application [*P < 0.05; one-way analysis of variance (ANOVA) followed by Bonferroni multiple-comparison test versus 0 hours]. (**E**) dsGFP expression in primary neurons transduced with *cfos*-dsGFP 0, 6, 24, and 48 hours after addition of PTX. Scale bars, 100 μm. (**F**) dsGFP-positive neurons expressed as a percentage of all 4’,6-diamidino-2-phenylindole (DAPI)−positive neurons at different time points after PTX application (**P < 0.01; ***P < 0.001; one-way ANOVA followed by Bonferroni multiple-comparison test versus 0 hours).

Activity-dependent promoters, typically of immediate early genes (lEGs), respond rapidly to increases in neuronal activity (21–23) and have been used to identify hyperactive cells recruited during seizures (24). Although seizures often start and terminate abruptly, they can occur in clusters with intervals comparable with the kinetics of IEGs (25). We therefore asked whether an activity-dependent promoter could be used to drive a therapeutic transgene to reduce neuronal excitability in closed loop. Epileptiform hyperactivity should reduce their likelihood to fire while leaving other neurons unaffected (Fig. 1A). Once seizures resolve, the genetic therapy should switch itself off, unless and until excessive activity occurs again (Fig. 1B).

## Results

### Activity-dependent gene therapy follows neuronal dynamics, decreases network hyperexcitability in vitro

We used an adeno-associated virus (AAV) to express a destabilized version of the fluorescent reporter green fluorescent protein (dsGFP) under the promoter of an extensively characterized IEG, c-Fos (AAV9 *cfos*-dsGFP; shortened to *cfos*-dsGFP) (21–24), and verified that dsGFP expression dynamically follows epileptiform network activity in vitro. Primary neuronal cultures were infected at 7 days in vitro and grown either on multielectrode arrays (MEAs) to record network activity or on coverslips for imaging (Fig. 1, C to F). At 18 days in vitro, we blocked γ-aminobutyric acid type A (GABA_A_) receptors with picrotoxin (PTX) to mimic an epileptic seizure and then recorded network activity or, in parallel, fixed a subset of coverslips, at 2, 6, 24, and 48 hours in the continued presence of PTX. MEA recordings revealed an increase in network activity that peaked 6 hours after addition of PTX and then returned to baseline after 48 hours (Fig. 1D and fig.S1). c-Fos expression and dsGFP fluorescence followed a similar time course (Fig. 1, E and F, and fig. S2).

**Fig. 2:**
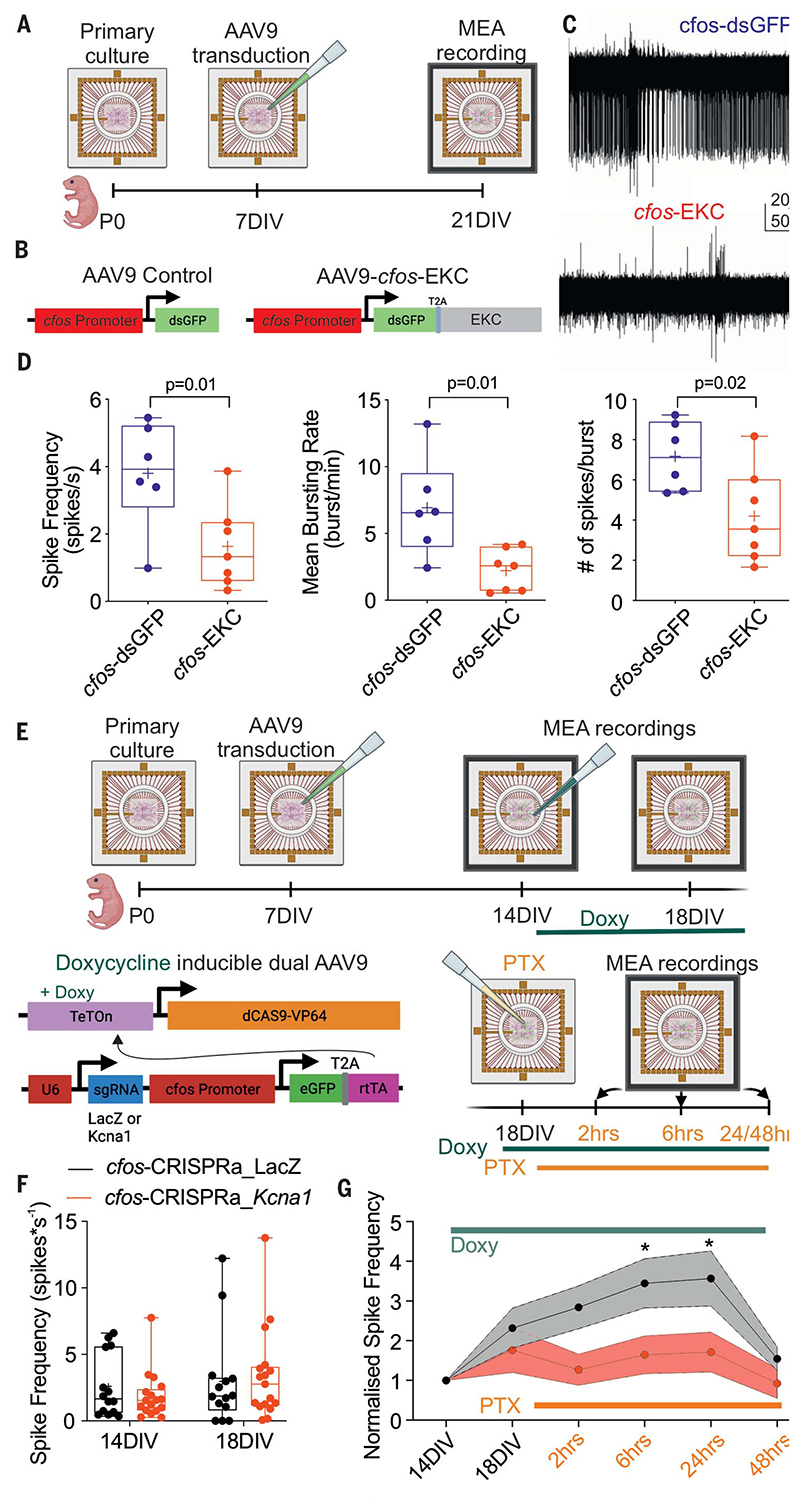
Activity-dependent gene therapy decreases epileptiform activity in vitro. (**A**) Timeline of MEA recordings in primary cultures. DIV, days in vitro. (**B**) Primary cultures were transduced with either cfos-dsGFP as control or cfos-EKC. (**C**) MEA traces. (**D**) Effect of cfos-EKC compared with cfos-dsGFP on spike frequency, mean bursting rate, and average number or spikes per burst. Student’s t test corrected for multiple comparisons, a = 0.02. (**E**) Timeline of MEA recordings in primary cultures transduced with dual AAVs controlled by doxycycline: one expressing an inducible dCAS9 fused to a transcriptional activator, and the other carrying an sgRNA targeting either the Kcna1 promoter or a LacZ sequence. (**F**) Spike frequency before PTX addition was similar between neurons transduced with cfos-CRISPRa_LacZ and cfos-CRISPRa_Kcna1. (**G**) Spike frequency over time normalized to network activity recorded at 14 days in vitro (*P < 0.05; two-way ANOVA followed by Bonferroni multiple-comparison test; interaction time × sgRNA, P = 0.006). (F) and (G) use the same color scheme.

We next placed a transgene that decreases neuronal and network activity under the same *cfos* promoter. We used a codon-optimized version of *KCNA1*, which encodes the potassium channel Kv1.1, with an Ile400Val mutation to bypass the need for posttranscriptional editing and facilitate recovery from inactivation (15). We refer to this engineered potassium (K^+^) Channel as EKC. Kv1.1 overexpression decreases both neuronal excitability and synaptic neurotransmitter release (26). Kv1.1 overexpression using KCNA1, EKC, or transcriptional upregulation has also been used in experimental antiepileptic gene therapies targeting excitatory neurons (11, 12, 15, 27, 28). Because of the innate propensity of primary neuronal cultures in vitro to exhibit bursting activity as they mature, we transduced neurons plated on MEAs at 7 days in vitro with either *cfos*-dsGFP or AAV9 *cfos*-EKC (*cfos*-EKC) and recorded network activity at 21 days in vitro (Fig. 2, A and B). As hypothesized, cultures treated with *cfos*-EKC showed less network activity than that of cultures treated with *cfos*-dsGFP (Fig. 2C). Spike frequency, burst frequency, and number of spikes per burst were all significantly lower in cultures treated with *cfos*-EKC (Fig. 2D and fig. S3). We also asked whether an experimental perturbation aimed at transiently increasing activity is followed by a rapid decrease in neuronal excitability, as would be expected from the activity-dependent promoter driving EKC expression (fig. S5). We increased spontaneous activity by adding PTX for 30 min and then silenced network activity for 2 hours with tetrodotoxin (TTX) before washing off the blockers and recording neuronal firing. We observed a net decrease in firing rate in dsGFP-positive neurons treated with *cfos*-EKC compared with *cfos*-dsGFP (fig. S5).

We asked whether the results with *cfos*-EKC would generalize to other activity-dependent promoters and transgenes (fig. S6). We compared the promoters of several IEGs (*cfos*, *arc*, and *egr1*) as well as synthetic activity−dependent promoters [enhanced synaptic activity-responsive element (ESARE) and NPAS4 robust activity marker (NRAM)], which have different properties (21, 29–31) (table S2), in combination with three transgenes: the control reporter dsGFP; EKC as above; or another potassium channel gene, *KCNJ2*, which encodes the muscle inward-rectifier Kir2.1 (32, 33). Although some promoter-transgene combinations were effective in reducing spiking and/or bursting, none was as consistent across the different electrophysiological measures as *cfos*-EKC (fig. S6).

We sought to determine whether *cfos*-EKC dampens the effect of a proconvulsant manipulation in vitro. However, the lower baseline activity in *cfos*-EKC-treated cultures (Fig. 2D) precluded a simple comparison with *cfos*-dsGFP. Instead, we modified a previously described method, CRISPR activation (CRISPRa), based on dual-AAV9 delivery of a single guide RNA (sgRNA) and a nuclease-defective “dead” Cas9 (dCas9) controlled by doxycycline (14, 34). One AAV9 carried a TeT-ON promoter driving dCas9 fused to a transcriptional activator. The other AAV9 expressed the *cfos* promoter driving the tetracycline trans-activator rtTa linked to enhanced GFP (EGFP) with a T2A peptide, as well as a U6 promoter driving either an sgRNA targeting the endogenous *Kcna1* promoter or an sgRNA designed to target the yeast LacZ gene as control (Fig. 2E) (14, 34). In this system, endogenous *Kcna1* overexpression was designed to be switched on only when all three of the following conditions are met: (i) neurons are co-transduced with both AAV9s, (ii) doxycycline is applied, and (iii) activity is sufficient to activate the *cfos* promoter. Primary cultures plated on MEAs were treated with both AAVs at 7 days in vitro. We then obtained an initial (baseline) recording of network activity at 14 days in vitro without doxycycline treatment. There was no difference in network excitability between cultures transduced with either *cfos*-CRISPRa_*Kcna1* or *cfos*-CRISPRa_LacZ (Fig. 2F). We then added doxycycline to activate CRISPRa and recorded from the same cultures 4 days later. We again observed no significant difference in network activity. Last, we added PTX and recorded from the same cultures 2, 6, 24, and 48 hours later. This revealed a clear difference between cultures treated with *cfos*-CRISPRa_Kcna1 and *cfos*-CRISPRa_LacZ. Indeed, PTX failed to increase network activity in cultures treated with *cfos*-CRISPRa_Kcna1 (Fig. 2G and fig. S7).

### Activity-dependent gene therapy decreases neuronal excitability in a transient, cell-autonomous, on-demand fashion

We next asked whether the strategy is effective in vivo. In the first set of experiments, we asked how the *cfos* promoter behaves in response to chemoconvulsant-evoked seizures. Mice were injected with either *cfos*-dsGFP or AAV9 *CaMKII-dsGFP* (shortened to *CaMKII-dsGFP*) in the visual cortex. The CaMKII promoter was chosen as a control because it has previously been used to bias expression to forebrain principal cells in constitutive antiepileptic gene therapies (10, 15). Two weeks later, we injected either pilocarpine into the same brain region to induce a focal seizure (35) or saline as a control (fig. S8) and sacrificed the mice 2 hours later. Animals treated with *CaMKII*-dsGFP showed widespread expression of GFP whether saline or pilocarpine was injected. By contrast, mice treated with *cfos*-dsGFP showed focal expression of GFP only after pilocarpine injection (fig. S8).

**Fig. 3:**
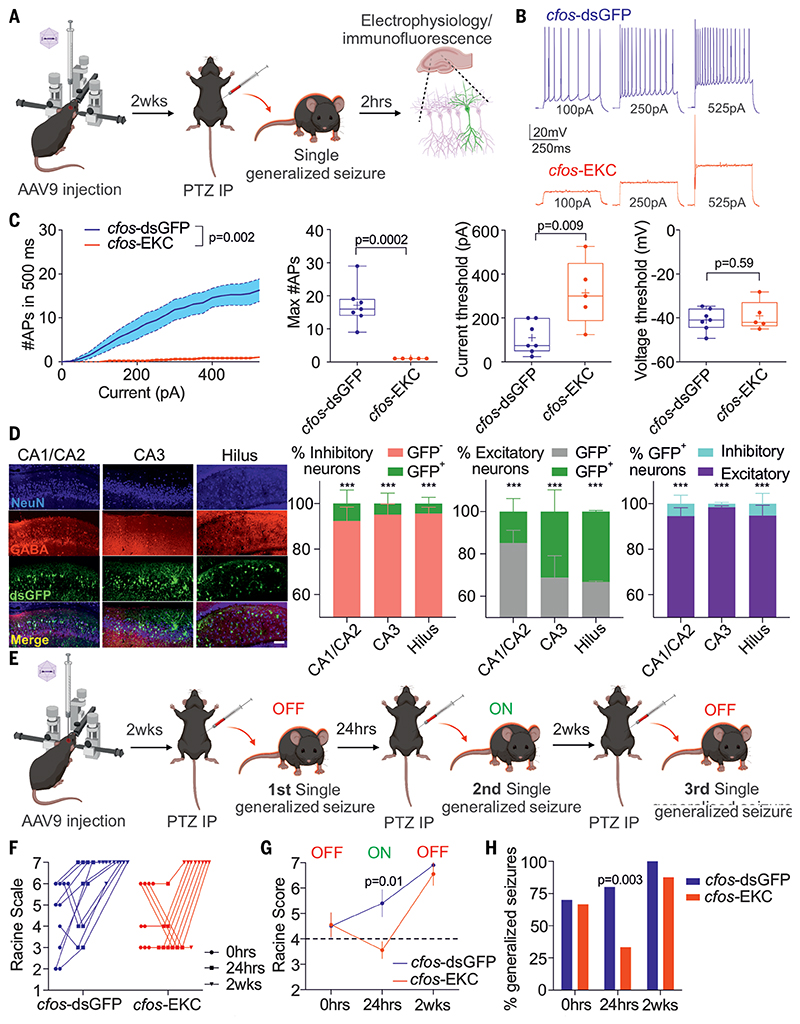
Activity-dependent gene therapy is effective in decreasing excitatory neuronal excitability and in protecting against sequential chemoconvulsant challenges. (**A**) Illustrative timeframe for electro physiology and immunofluorescence analysis. (**B**) Evoked trains of action potentials recorded in neurons transduced with either *cfos*-dsGFP or *cfos*-EKC. (**C**) Neuronal excitability parameters of neurons expressing either *cfos*-dsGFP or *cfos*-EKC after a single generalized seizure. (Left) Number of action potentials evoked by increasing current steps. (Middle) Maximum number of evoked action potential. (Right) Current and voltage thresholds. Input/output, two-way ANOVA; other graphs, Student’s t test corrected for multiple comparisons, α = 0.02. (**D**) Immunofluorescence images and analysis of the percentage of inhibitory neurons positive or negative for dsGFP, of excitatory neurons positive or negative for dsGFP, and of dsGFP positive neurons identified as excitatory or inhibitory (n = 3 animals) (***P < 0.001 two-way ANOVA followed by Bonferroni multiple-comparison test). Scale bar, 100 μm. (**E**) Illustrative timeline for repeated PTZ injections. OFF, basal *cfos*-driven transgene expression; ON, seizure-induced *cfos*-driven expression. (**F**) Raw Racine scale data in animals transduced with either *cfos*-dsGFP or *cfos*-KCNA1 and then injected with PTZ at three time points (0 hours, 24 hours, and 2 weeks). (**G**) Averaged Racine scores of data shown in (F). Racine score of >4 was considered as a generalized seizure. Two-way ANOVA followed by Bonferroni multiple comparison test. (**H**) Percentage of mice experiencing generalized seizures from (F) (χ^2^ test).

To test whether activity-dependent expression of EKC driven by the *cfos* promoter can affect neuronal excitability, we injected either *cfos*-dsGFP or *cfos*-EKC in the hippocampus of adult mice. Two weeks later, we elicited a single generalized seizure with an intraperitoneal pentylenetetrazole (PTZ) injection. Mice were monitored to verify that they reached stage 5 on a revised Racine scale (equivalent to a generalized tonic-clonic seizure) (36) and were sacrificed after 2 hours to prepare acute hippocampal slices for electrophysiological recordings from dsGFP-positive CA1 pyramidal neurons (Fig. 3A). dsGFP-positive neurons treated with *cfos*-EKC exhibited a profound decrease in excitability when compared with *cfos*-dsGFP treated neurons (Fig. 3, B and C), with robust decreases in the maximal firing frequency and in the slope that relates action potential frequency to depolarizing current injection. There was also an increase in current threshold for triggering the first action potential and an increase in the size of the after-hyperpolarization, but other parameters were not affected (Fig. 3C and fig. S9). The effect of activity-dependent EKC expression was more pronounced than previously observed with constitutive expression (11, 14). We were unable to measure the effect of *cfos*-EKC in the absence of seizures because we could not identify the transduced neurons without activation of the fluorescent reporter.

We also tested other activity-dependent promoter-transgene combinations. These revealed a consistent effect of EKC overexpression on action potential firing, independent of the promoter (figs. S10 and S11). When the *KCNJ2* transgene was used, however, neurons exhibited a hyperpolarized resting membrane potential, as expected from the intrinsic properties of this channel (32), but inconsistent changes in firing (figs. S10 and S11 and supplementary text).

To better understand which neurons were activated after a single generalized PTZ-evoked seizure, we performed immunohistochemical analysis in different hippocampal regions (CA1/2, CA3, and hilus) in animals treated with different IEG promoters driving dsGFP in the hippocampus (Fig. 3D and figs. S12 to S17). dsGFP driven by *cfos* was mainly expressed in excitatory neurons, with a very low percentage of inhibitory neurons or of parvalbumin-positive neurons expressing the reporter (Fig. 3D and fig. S15). Although ESARE-driven dsGFP expression was mainly in excitatory neurons, dsGFP driven by a minimal arc promoter (*mArc*) showed a significantly higher percentage of expression in inhibitory neurons (≈12%) (figs. S14, S16, and S17). With *cfos*-dsGFP, there was a trend for a lower percentage of excitatory neurons in CA1/2 to express dsGFP compared with CA3 and hilus (Fig. 3D).

We hypothesized that expression of EKC in response to an evoked generalized seizure should attenuate the effect of a second chemoconvulsant challenge if delivered during the time that potassium channel overexpression persists, but that this protective effect should fade with time. We therefore performed three consecutive intraperitoneal PTZ injections in the same animals (Fig. 3E). We first expressed either *cfos*-dsGFP or *cfos*-EKC in both hippocampi and, after 2 weeks, gave the first PTZ injection. This injection triggered a single generalized seizure with no difference in severity between the two groups (Fig. 3, E to H), which was as expected because the *cfos* promoter is normally inactive under the baseline condition. We then administered a second PTZ injection 24 hours later. This led to markedly attenuated seizures in the animals injected with *cfos*-EKC compared with those injected with *cfos*-dsGFP, with the majority of animals failing to reach a Racine score of 4 (Fig. 3, E to H). Last, to test whether this effect persists, we gave a third PTZ injection after 2 weeks, by which time we anticipated that overexpression of Kv1.1 channels had returned to baseline, as expected from their membrane persistence (37). At this time point, no difference was observed between the two groups (Fig. 3, E to H). Activity-dependent induction of EKC expression thus protects against generalized convulsions, which are associated clinically with mortality and morbidity, in a time-limited manner.

### Activity-dependent gene therapy does not affect normal behavior

**Fig. 4:**
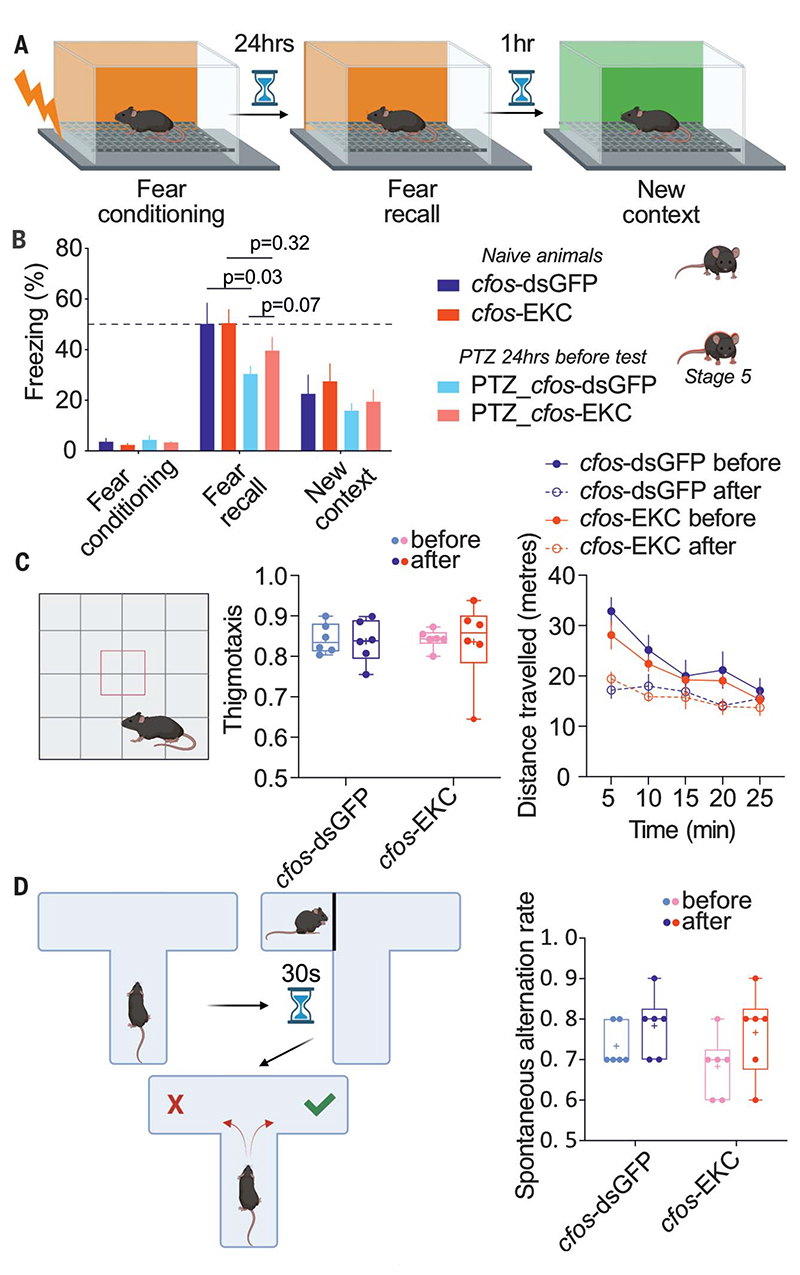
Activity-dependent gene therapy does not affect normal behavior. (**A**) Schematic of the CFC test. (**B**) Percentage of freezing time for naïve or PTZ-treated mice injected with either *cfos*-dsGFP (n = 7 and 8, respectively) or *cfos*-EKC (n = 7 and 9, respectively) during fear conditioning, fear recall, and in a new context. Two-way ANOVA followed by Bonferroni multiple-comparison test. (**C**) Open field test. Shown is thigmotaxis (fraction of time spent in the periphery) and distance traveled before and after either *cfos*-dsGFP or *cfos*-EKC injection in both hippocampi. (**D**) Spontaneous T-maze alternation test. Shown are the spontaneous alternation rates before and after either *cfos*-dsGFP or *cfos*-EKC injection.

A potential limitation of *cfos*-EKC treatment is that it might interfere with normal brain function. Indeed, the *cfos* promoter is recruited during physiological behaviors such as memory formation and learning (38). We therefore asked whether salient sensory and aversive stimuli could lead to the overexpression of Kv1.1 sufficient to interfere with normal behavior. *Cfos* activation has previously been harnessed to manipulate a memory trace or engram, with behavioral readout in a contextual fear conditioning (CFC) task: Chemogenetic or optogenetic modulation of neurons “tagged” in a *cfos*-dependent manner during this test can alter fear recall 24 hours after memory consolidation (39–41). We repeated this behavioral paradigm after bilateral intrahippocampal expression of either *cfos*-dsGFP or *cfos*-EKC and performed the CFC test 2 weeks later (Fig. 4A). We tested this paradigm both in naïve animals and in animals that had received a single PTZ injection 24 hours before fear conditioning (leading to a stage 5 seizure in all such animals). In the naïve animals, no difference in freezing behavior was observed between *cfos*-dsGFP− and *cfos*-EKC−treated groups, either during fear conditioning or 24 hours later during fear recall, or when animals were exposed to a new context (fig. S18). In animals that had received a PTZ injection, we observed a nonsignificant trend for greater freezing during fear recall in the *cfos*-EKC group than in the *cfos*-dsGFP group (Fig. 4B). When animals were tested in several other behavioral tasks, *cfos*-EKC−expressing animals again performed similarly to *cfos*-dsGFP− expressing animals: No differences were observed before or after viral injection (Fig. 4, C and D, and fig. S19) in an open field test to monitor anxiety and locomotor activity (Fig. 4C), a T-maze test to assess working memory (Fig. 4D), or an olfactory discrimination test (fig. S19).

### Activity-dependent gene therapy suppresses spontaneous seizures and interictal activity

**Fig. 5:**
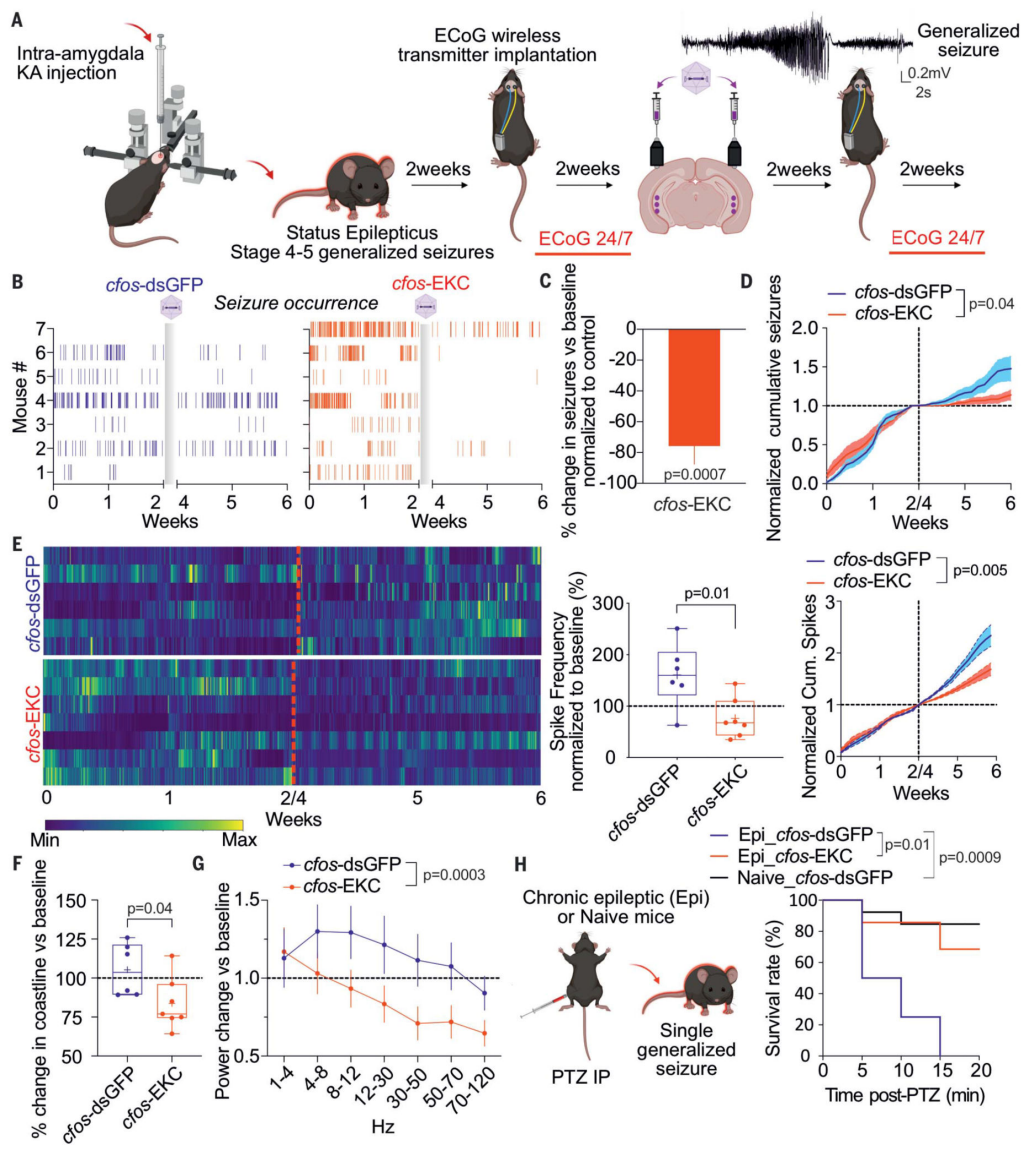
Activity-depieindent gene therapy decreases spontaneous generalized seizures and interictal spikes and protects against a subsequent chemoconvulsant challenge. (**A**) Schematic of the preclinical trial. (**B**) Seizures (vertical bars) over time for each mouse. Gray boxes indicate the viral injection, either with *cfos*-dsGFP or *cfos*-EKC, followed by a 2-week period to allow viral expression. (**C**) Percentage change in spontaneous generalized seizures after *cfos*-EKC treatment compared with baseline, normalized to the same percentage of change in *cfos*-dsGFP treated mice. One Sample t test versus 0 hours. (**D**) Weighted cumulative plot normalized by the total seizure count before treatment. Two-way ANOVA. (**E**) (Left) Number of spikes per hour plotted against time. Red dotted lines indicate the viral injection, with either *cfos*-dsGFP or *cfos*-KCNA1. Spike rates were normalized to the maximum for each animal (yellow, maximum; blue, minimum spike rate). (Middle) Spike frequency normalized to baseline (before viral injection) (Student’s t test). (Right) Weighted cumulative plot normalized by the total interictal spike count before viral treatment (two-way ANOVA). (**F**) Percentage change in coastline normalized to baseline (before viral injection). Student’s t test. (**G**) Percentage power change versus baseline in different frequency bands (two-way ANOVA). (**H**) Chronic epileptic animals or naïve animals (injected with *cfos*-dsGFP) received an intraperitoneal PTZ injection at the end of the study. Kaplan-Meier plot showing survival rate. Log-rank (Mantel-Cox) test.

in a chronic model of intractable epilepsy Although treatment with *cfos*-EKC is effective in protecting against a second chemoconvulsant challenge delivered shortly after a first, it remained to be determined whether it could modify the disease course in epilepsy, in which seizures occur sporadically. We therefore switched to a model of chronic limbic epilepsy. We used a clinically relevant model of drug-resistant epilepsy that consists of an intra-amygdala kainic acid (KA) injection to induce a period of status epilepticus (14, 42, 43). Mice started to exhibit spontaneous seizures after 2 weeks and were implanted with wireless electrocorticogram (ECoG) transmitters (Fig. 5A). We recorded spontaneous generalized seizures for 2 weeks and then randomized animals for injection with either *cfos*-dsGFP or *cfos*-EKC in both hippocampi. Two weeks later, after waiting for viral expression, the ECoG was recorded for 2 more weeks (Fig. 5A). *cfos*-EKC−treated animals showed a robust decrease in the number of spontaneous seizures when compared with animals receiving *cfos*-dsGFP (Fig. 5, B to D).

We also analyzed interictal spike activity in the same animals (Fig. 5E). We observed a net decrease in spike activity in animals treated with *cfos*-EKC compared with *cfos*-dsGFP (Fig. 5E), with a trend to more consistently follow a circadian rhythm (fig. S20). Despite a slight decrease in seizure frequency with time in *cfos*-dsGFP−treated animals, interictal spike frequency increased, suggesting that this phenomenon is a natural evolution of this chronic model. By contrast, animals treated with *cfos*-EKC showed significant decreases in both spontaneous seizures and interictal spike activity, suggesting a broader impact of the treatment than only raising the seizure threshold. Indeed, treatment efficacy was reflected in significant decreases in ECoG coastline (a measure of overall activity) and in total power, especially at higher frequencies (Fig. 5, F and G).

We also asked whether the activity-dependent transgene expression was maintained in the absence of overt seizures. *cfos*-dsGFP was injected bilaterally in the dentate gyrus. Two weeks later, KA was injected unilaterally, also in the dentate gyrus. After 5 days, at which stage interictal spikes occur without seizures (and dsGFP induced by KA has been degraded), we sliced the brain and observed ipsilateral *cfos*-dsGFP expression (fig. S21). This finding suggests that interictal activity is sufficient for activation of the treatment.

At the end of the chronic epilepsy study, we asked whether the treatment protected animals from a lethal dose of PTZ (Fig. 5H). We observed a significant survival advantage in animals injected with *cfos*-EKC compared with *cfos*-dsGFP, with a survival rate close to that of *cfos*-dsGFP−transduced naïve animals injected with the same PTZ dose (85%) (Fig. 5H). We also tested the effect of *cfos-KCNJ2* on spontaneous seizures in the same chronic epilepsy model (fig. S22). In contrast to *cfos*-EKC, no impact on seizure frequency was observed in animals injected with *cfos-KCNJ2* when compared with *cfos*-dsGFP (fig. S22), implying that transient Kir2.1 expression does not have an antiepileptic action.

Last, we asked whether treatment with *cfos*-EKC affected performance in behavioral tests that are affected by chronic epilepsy. Having quantified several behavioral defects in chronic epileptic animals compared with naïve animals, we injected either *cfos*-dsGFP or *cfos*-EKC in epileptic animals and waited 4 weeks to measure the same behavioral parameters (figs. S23 to S25). We observed no worsening in animals treated with *cfos*-EKC compared with *cfos*-GFP in either T-maze, open field, or olfactory discrimination tests (figs. S23 to S25).

### Activity-dependent gene therapy suppresses epileptiform activity in human neurons

**Fig. 6:**
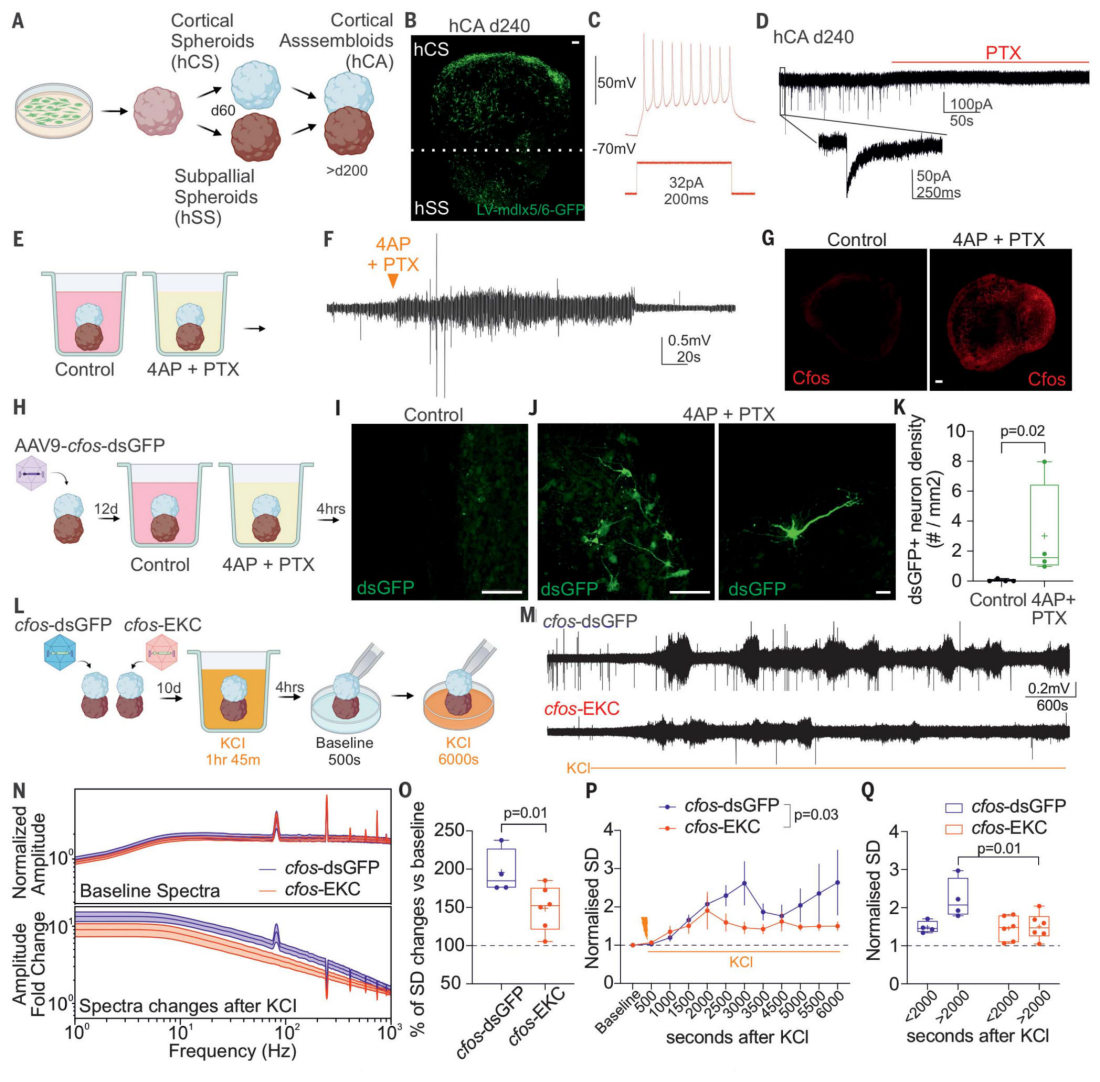
Activity-dependent gene therapy decreases epileptiform activity in hCAs. (**A**) Schematic representation for the generation of human fore brain assembloids after fusion of hCSs and hSSs. (**B**) Inhibitory neurons migrate from hSS to hCS, recapitulating human cortical development. Scale bar, 100 μm. (**C** and **D**) Trains of evoked action potentials and spontaneous inhibitory postsynaptic events recorded in excitatory neurons in cortical assembloids at the latest stage of differentiation (d240). (**E**) Cortical assembloids were maintained either in control medium or in a medium with 4AP and PTX. (**F**) Local field potential showing an increase in activity after addition of 4AP and PTX (orange triangle). (**G**) Immunofluorescence images of c-Fos obtained 4 hours after control medium (left) or medium supplemented with 4AP and PTX (right). Scale bar, 100 μm. (**H**) Cortical assembloids were transduced with *cfos*-dsGFP and then, 12 days later, moved to either control medium or medium supplemented with 4AP and PTX. (I to K) After 4 hours, cortical assembloids were fixed and stained for dsGFP, and the density of dsGFP-positive neurons was plotted. Scale bars, (**I**) and (**J**), 100 μm; (**K**) 10 μm. Student’s t test. (**L**) Schematic representation for a test of activity-dependent gene therapy in hCAs. (**M**) Local field potential showing a decrease in activity after addition of KCl (orange line) in hCAs transduced with *cfos*-EKC compared with *cfos*-dsGFP. For the purpose of illustration, the voltage range illustrated is ± 0.5 mV. (**N**) (Top) Baseline LFP amplitude spectra for hCAs treated with *cfos*-dsGFP or *cfos*-EKC. (Bottom) Amplitude changes across frequencies after KCl. (**O**) Percentage change in the median standard deviation (SD), normalized to baseline activity, as a proxy for network activity. Student’s t test. (**P**) Time course of SD during the experiment. Two-way ANOVA. (**Q**) Change in SD in hCA transduced with either *cfos*-dsGFP or *cfos*-EKC divided into two periods: <2000 s before *cfos* activation and >2000 s after *cfos* activation (two-way ANOVA followed by Bonferroni multiple comparison test).

We asked whether *cfos* can be activated by epileptiform activity in human neurons. We used an induced pluripotent stem cell line to derive human cortical assembloids (hCAs) by fusing cortical spheroids (hCSs) with subpallial spheroids (hSSs) (Fig. 6A) (44–47). Immunofluorescence analysis at different time points showed expression of hCS and hSS region-specific transcription factors [forkhead box G1 (FOXG1), paired box 6 (PAX6), and NK2 homeobox 1 (NKX2.1)] and mature neuronal markers [microtubule associated protein 2 (MAP2), NeuN, and GABA)] (fig. S26). We demonstrated successful fusion of hCSs and hSSs, with inhibitory neurons migrating to, and integrating with, the cortical side (Fig. 6B). We then performed electrophysiological recordings in neurons located in the hCS area of acute hCA slices and showed that neurons can fire trains of action potentials and exhibit spontaneous PTX-sensitive inhibitory postsynaptic currents, confirming that interneurons integrate in the network (Fig. 6, C and D). We treated mature hCAs with 4-aminopyridine (4AP) and PTX to increase network excitability and recorded epileptiform activity using local field potentials (Fig. 6, E and F). Four hours later, we observed c-Fos-positive neurons that were not present when hCAs were treated with a control solution (Fig. 6G). Last, we transduced hCAs with AAV9 *cfos*-dsGFP and after 12 days incubated them in either a control solution or 4AP and PTX (Fig. 6H). No dsGFP-positive neurons were present in the control condition (Fig. 6I). By contrast, we observed dsGFP-positive neurons in hCAs treated with 4AP and PTX, suggesting that this minimal promoter is also activated in human neurons after epileptiform activity (Fig. 6, J and K). Last, we tested the ability of *cfos*-EKC to suppress epileptiform activity in hCAs. We transduced hCAs with a lentivirus expressing either *cfos*-dsGFP or *cfos*-EKC and after 10 days treated them for 1 hour and 45 min with 55 mM KCl (fig. S27). Four hours later, we recorded baseline local field potential activity and then added 55 mM KCl a second time (Fig. 6L). We observed a clear reduction in overall network activity in hCAs transduced with *cfos*-EKC compared with *cfos*-dsGFP (Fig. 6, M to Q).

## Discussion

We have reported an activity-dependent, cell-autonomous, on-demand gene therapy that follows network dynamics, driving the expression of a transgene that in turn decreases neuronal excitability (Fig. 1, A and B). This approach is specific for neurons that participate in pathological network activity but is also time-limited in that transgene expression persists only for as long as neurons are hyperactive (Fig. 3, E to H). In a chronic model of epilepsy, we achieved a greater decrease in seizure frequency than previously reported for gene therapy with constitutive promoters or with widely used antiseizure drugs (11–13, 43), and without deleterious effects on normal behaviors (Figs. 4 and 5). Given that the components of activity-dependent gene therapy are normal mammalian genetic elements packaged in a well-tolerated viral vector that is already in the clinic, there is a relatively straightforward path to first-in-human studies. The translational potential is underlined by the preliminary evidence of effectiveness in hCAs (Fig. 6).

*Cfos* tagging has been used to track the recruitment of neurons in pathological behaviors and as a test of causality in CFC (39–41). The combination of *cfos* with EKC proved highly effective in downregulating neuronal excitability after chemoconvulsants and in suppressing spontaneous seizures, without deleterious effects in a battery of behavioral tests. We observed less consistent effects on neuronal excitability with other promoters. Why other IEG promoters, and synthetic promoters, were less effective is unclear. Among factors determining their potential utility are the different time courses of activation and deactivation, and the triggering mechanisms for each promoter. For example, mArc activation has been linked to synaptic transmission and plasticity processes rather than simply responding to increases in neuronal activity, as is the case for *cfos*, although the intermediate intracellular signaling cascades overlap (21). We also found that *KCNJ2* could not be substituted for EKC, even though it profoundly hyperpolarized neurons when driven in an activitydependent manner (fig. S4). If anything, animals treated with AAV9 *cfos-KCNJ2* had a worse outcome in the chronic epilepsy study when compared with animals injected with *cfos*-dsGFP (fig. S10). A possible explanation for the efficacy of Kv1.1 overexpression is that it reduces not only excitability but also neurotransmitter release through a modulatory effect on the presynaptic action potential waveform (supplementary text) (26, 48).

It remains to be determined whether the powerful antiepileptic effect of *cfos*-EKC depends on suppressing the excitability of a small number of “detonator” or “hub” neurons responsible for triggering seizures, or of a network of neurons whose activity is prone to reaching a critical point (20, 49). The data are therefore silent on whether successive seizures arise from a stereotypical or variable pattern of neuronal recruitment in an ictogenic network.

Because patients with focal epilepsy often experience two or more seizures within 24 hours (25), *cfos*-EKC would be expected to abort such clusters. However, we observed the persistence of the antiepileptic treatment in the chronic model despite evidence from the PTZ injections that the effect of a single evoked seizure faded by 2 weeks (Figs. 3 and 5). A possible explanation is that in the chronic epilepsy model, interictal discharges repeatedly reactivated the *cfos* promoter, so that the epileptiform activity remained below threshold for generalization. This is supported by the findings that interictal activity is sufficient to activate *cfos*-driven dsGFP expression (fig. S21) and that treated animals were relatively protected from the effects of a chemoconvulsant challenge despite a prolonged absence of overt seizures (Fig. 5H).

A cell-autonomous self-regulated tool that normalizes network dynamics has potential clinical applications beyond epilepsy. Several other neuropsychiatric diseases are characterized by circuit hyperactivity, including early stages of schizophrenia [in which anterior hippocampal hyperactivity has been implicated (50)], Parkinson’s disease [subthalamic nucleus (51)], migraine and cluster headache [posterior hypothalamus (52)], and obsessive-compulsive disorder [anterior cingulate cortex (53)]. Early hyperactivity has also been documented in mouse models of Alzheimer’s disease and is associated with high levels of arc expression (54). In this case, arc or ESARE could represent appropriate therapeutic activity-dependent promoters.

## Supplementary Material

Supplementary Materials

## Data Availability

All Python and Matlab scripts are available at https://github.com/KullmannLab/Qiu_et_al_2022. PyEcog software is available here: https://www.pyecog.com. All the vectors used in this manuscript will be made available on a suitable platform. All data are stored in the UCL cloud database. All raw data supporting the main and supplementary figures are available here: https://doi.org/10.5522/04/20867117.
