# Supplementary material for: On-demand cell-autonomous gene therapy for brain circuit disorders
Source: Science. Author manuscript; Available in PMC 2022 Dec 28. (PMC7613996; doi:10.1126/science.abq6656)
Supplement: Supplementary Materials [file EMS158742-supplement-Supplementary_Materials.pdf]

Materials and Methods

Study Design

All the treatment allocations were randomized and researchers were blinded to virus injected during recordings and analysis. The aim of this study was to test the hypothesis that activity-dependent gene therapy is transient, closed-loop, and can treat intractable temporal lobe epilepsy without affecting physiological behavior. The *in vivo* experiments were designed to achieve a power >0.8 with an $\alpha = 0.05$. For *in vivo* experiments the 3Rs guidelines for animal welfare were followed. Outliers were not excluded and at least 3 independent repetitions were performed for *in vitro* experiments.

Animals and ethics

All animal work was conducted in accordance with the Animals (Scientific Procedures) 1986 Act, and approved by the local ethics committee. All procedures mentioned in this project were carried out in accordance with appropriate project and personal licenses, and following ARRIVE guidelines. Male and female wild-type (WT), C57BL6 mice (15-30g, 2-3 months) were obtained from Envigo, Charles River Laboratory, or the UCL breeding facility. They were housed with free access to food and water in 12 h light/dark cycles, in an enriched environment and whenever possible in groups of 2 or 3. Animals were acclimatized for at least 7 days before any procedures or behavioral assessments.

Plasmid Design and Cloning

The *KCNA1*$^{\text{co-I400V}}$ sequence (Engineered potassium channel, EKC) was previously published (*15*). The lentiviral vector pCCL-*cfos*-dsGFP vector was generated by cloning the *cfos* promoter sequence modified from Fos-tTa (Addgene #34856). The

pCCL-cfos-dsGFP-T2A-EKC plasmid was produced by subcloning dsGFP-T2A-EKC into *cfos*-dsGFP using BamHI and SalI. The pX552-*cfos*-dsGFP and pX552-dsGFP-EKC constructs were produced with restriction enzyme digestion and ligation into an empty PX552 construct backbone using MluI and SalI (Addgene #60958). The dsGFP-T2A-*KCNJ2* fragment was synthesized by GeneArt customized DNA synthesis service (ThermoFisher) and was used to replace the dsGFP-T2A-EKC cassette in the previous construct via restriction enzyme digestion and ligation. The miniArc (mArc) promoter was amplified from a pAAV-ESARE-miniArc-ERT2-Cre-ERT2, a gift from Francesco Gobbo (University of Edinburgh), using PCR to introduce a 5' MluI restriction site. mArc was subsequently subcloned into the pX552-*cfos*-dsGFP vector using MluI and EcoRI restriction sites to replace the *cfos*. The same subcloning protocol was used to generate mArc-dsGFP-EKC and mArc-dsGFP-*KCNJ2* AAV vectors. The steps for creating ESARE constructs were as described above. The EGR1 promoter sequence (516bp) was extracted from the sequence upstream of the mouse EGR-1 gene locus(*55*). This mouse EGR-1 promoter fragment, flanked by MluI and EcoRI restriction enzyme sites, was synthesized by GeneArt (ThermoFisher). It was subcloned as a promoter into vector backbones pX552- dsGFP-EKC and pX552 -dsGFP-*KCNJ2,* using Mlu1 and EcoR1 restriction sites. The NRAM enhancer sequence as published(*31*). The enhancer repeats were followed by the human promoter sequence (hcFosP)(*30*) to form the NRAM promoter. This NRAM promoter was synthesized by GenScript and cloned into pX552 backbone plasmids as above. A pLenti-U6-sgRNA19-*cfos* construct was produced by cloning of the *cfos* promoter sequence from pCCL-*cfos*-dsGFP-T2A-EKC into a pLenti-U6-sgRNA19 backbone (gift from V. Broccoli, San Raffaele Institute) using XhoI and AgeI restriction sites. sRNA19 targets *Kcna1* as previously

reported *(14)*. The rtTA-EGFP construct was synthesized by GeneScript and subcloned into pCCL-U6-sg19-*cfos*, using BamH1 and Age1, to produce pLenti-U6-sgRNA19-*cfos*-rtTA-T2A-EGFP. A control construct expressing sgLacZ was produced by amplifying a U6-sgLacZ cassette from pLenti-U6-sgLacZ-Hpgk-rtTA using PCR primers, and subsequent subcloning into pCCL-U6-sgRNA19-*cfos* using PpuMI and XhoI to produce pCCL-U6-sgLacZ-*cfos*-rtTA-T2A-EGFP. The U6-sgRNA19-*cfos*-rtTA-T2A-EGFP and U6-sgLacZ-*cfos*-rtTA-T2A-EGFP fragments were then amplified with PCR using MluI_F and SalI_R primers. An empty AAV backbone was produced by MluI and SalI restriction digestion of pAAV-U6-sgRNA19-hSyn-rtTA (gift from Dr. Gaia Colasante, San Raffaele Institute). The U6-sgRNA-*cfos*-rtTA-T2A-EGFP and U6-sgLacZ-*cfos*-rtTA-T2A-EGFP cassettes were subsequently cloned into this backbone to produce pAAV-U6-sgRNA19-*cfos*-rtTA-T2A-EGFP and pAAV-U6-sgLacZ-*cfos*-rtTA-T2A-EGFP. The following constructs: pAAV-CaMKII-tdTomato-T2A-rtTA-U6-sgRNA19; the control construct pAAV-CaMKII-tdTomato-T2A-rtTA-U6-sgLacZ, and pAAV-TetON-dCas9-VP64 were previously published(*14*).

Viral vector synthesis

Lentiviral synthesis. Lentiviruses were synthesized in-house using pCCL-*cfos*-dsGFP and pCCL-*cfos*-dsGFP-EKC transfer plasmids as in Carpenter et al. 2021(*56*). Human embryonic kidney (HEK) 293T cells were maintained at 37°C in a humidified 5% CO_2 environment, and transfected with second-generation lentiviral packaging, envelope and transfer plasmids at a ratio of 2:1:2.25. The cell medium containing viral particles was harvested 48 hours after transfection. The viral medium was clarified by centrifugation at 1000 rpm for 3 minutes at 4°C and passed through a

0.45 µm filter. The viral particles were concentrated by centrifugation at 20,000 rpm for 2 hours at 4°C (Beckman Ultracentrifuge). The viral pellet was then re-suspended in 200µl PBS after a 6-hour incubation at 4°C.

AAV synthesis. AAV synthesis was carried out using a protocol adapted from Grieger *et al*, 2006 (*57*). HEK 293T cells were plated on 20 x 150 cm^2 Petri Dishes (Thermo Scientific™ Nunc™ Cell Culture/Petri Dishes). Once at 80% confluency, cells were co-transfected with helper, cap-rep and transfer plasmids at a 3:1:1 ratio, using PEI MAX transfection reagent (PEI MAX - Transfection Grade Linear Polyethylenimine Hydrochloride (MW 40,000)) at a ratio of approximately 2 PEI : 1 DNA in Opti-MEM (Opti-MEM I Reduced Serum Media). Typical viral production time was 96-120 hours after transfection with AAV9 constructs. The viral particles were purified by centrifugation using an iodixanol gradient. The layer containing viral particles was carefully withdrawn using a 19-gauge needle from the side of the tube. The titers of AAVs produced in-house were calculated using AAVpro® Titration Kit (Takara). For in-house AAV production, all viruses used in experiments had an estimated titer of at least 10^{13} vg/ml, sufficient for *in vitro* and *in vivo* applications. AAV9-*cfos*-LacZ-dCas9A and AAV9-*cfos*-*Kcna1*-dCas9A viruses were produced by Vector Builder with quoted titers of > 10^{12} vg/ml.

Primary Neuronal Cultures

Primary cortical neuronal cultures were prepared from postnatal 0-1 (P0-1) WT C57BL/6J mouse pups, as previously described (*56*). Cells were maintained in Neurobasal A medium (Neurobasal A, Invitrogen, # 10888022) supplemented with B27 (Invitrogen, #17504044), Glutamax (Invitrogen, #35050038), and 1/1000 Penicillin-Streptomycin (5,000 U/ml, TFS, #15070063). With the exceptions of MEA

experiments, neurons were plated at a density of 120,000 – 150,000 cells/well on poly-L-lysine (10 mg/ml, in borate buffer) treated coverslips (13mm, VWR, #631-0148) in 24-well cell culture plates. Neurons were maintained at 37 °C in a humidified 5% CO_2 environment, and 50% of the media was replaced each week.

Lentiviral transduction of primary cultures

Lentiviral transduction of cortical neurons was performed on DIV 1. Neurons were incubated with the lentivirus for 24 hours and then all of the virus-containing media was removed and replaced with preconditioned media (*56*).

AAV transduction of primary cultures

AAV transduction of cortical cultures was performed between DIV 6 and DIV 8. Cells were transduced by directly adding the virus into the culture media at a MOI (multiplicity of infection) of >5000. Cultures were monitored for 24 hours post-transduction for signs of toxicity. If no cytotoxicity was observed, the AAV was left in the media. If some toxicity was apparent, a 50% media change was performed to reduce the number of viral particles.

***In vitro* whole-cell current-clamp recordings**

For current-clamp recordings of culture neurons, the internal solution contained (in mM): 126 K-gluconate, 4 NaCl, 1 $MgSO_4$, 0.02 $CaCl_2$, 0.1 1,2-Bis(2-aminophenoxy)ethane-N,N,N′,N′-tetraacetic acid (BAPTA), 15 Glucose, 5 HEPES, 3 ATP-Na_2, 0.1 GTP-Na_2, pH 7.3. The extracellular (bath) solution contained (in mM): 140 NaCl, 4 KCl, 2 $CaCl_2$, 1 $MgCl_2$, 10 HEPES, 10 glucose, pH 7.3. D-(−)-2-amino-5-phosphonopentanoic acid (D-AP5; 50 µM), 6-cyano-7-nitroquinoxaline-2,3-dione

(CNQX; 10 μM) and picrotoxin (PTX; 30 μM) were added to block synaptic transmission. Cultured cortical neurons grown on coverslips were used for electrophysiological recordings. At 1DIV neurons were transduced with lentivirus *cfos*-dsGFP or *cfos*-EKC as described above. The electrophysiology experiments were performed on 18-20DIV. Experiments were performed on pyramidal neurons identified by their shape as previously described (*58*). Prior to patch clamp recordings, 50% of the medium was removed, and conserved, and cells were incubated for 30 minutes with 30μM PTX. The cells were then treated by exchanging the reserved 50% medium containing 1 μM tetrodotoxin (TTX) for 2 hours to prevent further dsGFP expression. The coverslips were washed at least twice in extracellular solution to wash out TTX just before the experiment. Patch clamp pipettes were pulled from thin wall capillary (1.5OD, 1.17ID) (Harvard Apparatus) with a two-step vertical puller. Action potentials were counted only if the voltage crossed 0 mV. All the recordings were carried out from neurons held at -70mV at room temperature with continuous perfusion of the extracellular solution. Electrophysiological recordings were made with a Multiclamp 700A amplifier (Axon Instruments, Molecular Devices). The amplifier was used in combination with Power 1401 (CED) and Signal 6.0 software (Cambridge Electronic Design, Ltd). The data were filtered at 10 kHz and digitized at 50 kHz (BNC-2090, NI-6221, National Instruments). Bridge balance was applied. The current step protocol consisted of 500ms 10pA current steps from -20pA to 250pA. If no action potential was evoked, the neuron was injected with a higher current step of 350pA.

Multi-electrode arrays (MEAs)

6-well-MEA chambers with 60 electrodes were purchased from MultiChannel Systems (60-6wellMEA200/30iR-Ti). The bottoms of wells were coated with Bovine Serum (TFS, #261700430) or HI-Fetal Bovine Serum (TFS) for 2-24 hours, washed with sterile dH_20, air-dried and then coated with a fresh solution of Poly-L-lysine (1mg/ml in borate buffer, Sigma-Aldrich P2636) and laminin (laminin from Engelbreth-Holm-Swarm murine sarcoma, #L2020-1MG, and PBS (Phosphate-Buffered Saline, pH = 7.4, TFS, #10010023) in a ratio of 4:1:55. Cortical neurons were plated directly onto the electrodes with a high density of 90,000 – 100,000 cells/well. Neurons were transduced with AAV on DIV6-8 with MOI > 5000. For the recordings, MEA chambers were placed on the MEA recording setup, which was grounded, fixed on an air-supported platform and enclosed in a Faraday cage. The recording chamber was maintained at 37°C with a 1-channel temperature controller (Multichannel systems, #TC01). For experiments that required repeated recordings from the same chamber, the top was tightly covered with sterile Parafilm. Raw data were collected using 'MC_Rack' (V 4.6.2, MultiChannel Systems). The sampling frequency was 25kHz and the recording time was 5-10 minutes per session. Data processing and analysis were performed with MatLab based software, 'SPYCODE', which was a generous gift from Drs Ilaria Colombi and Michela Chiappalone (*60*). The data analysis pipeline consisted of data conversion, data filtering, baseline thresholding, spike detection and burst detection. The data filtering used a cut-off frequency of 300 Hz to select Multi-Unit-Activity components of the signal. The threshold for spike detection was set as 10x the standard deviation. Channels with high levels of noise were removed from the dataset. Bursts were defined as clusters of spikes that were simultaneously detected in at least 50% of the electrodes. The

algorithm to detect and analyze all the parameters has been described in Pasquale et al. 2010 (*61*).

Ex vivo whole cell current clamp recordings

Mice were lightly anesthetized with isoflurane and then administered with a lethal dose of pentobarbital (300-600 mg/kg, intraperitoneally). Once a deep plane of anesthesia was achieved, as indicated by a reduction in respiratory rate and complete absence of reflexes, the mouse was transcardially perfused with cold (2-4 °C), carbogenated (95 % O2/5 % CO2) sucrose-ACSF, containing (in mM): 87 NaCl, 2.5 KCl, 25 $NaHCO_3$, 1.25 NaH_2PO_4, 25 glucose, 75 sucrose, 7 $MgCl_2$, 0.5 $CaCl_2$. The brain was then excised from the skull and cooled for 1 minute in a beaker containing a semi-frozen ‘slush’ of carbogenated sucrose-ACSF. The brain was placed on filter paper (Whatman) in a petri-dish and surrounded by sucrose-ACSF: the cerebellum and the rostral third of the frontal cortices were removed, and the brain was hemisected along the sagittal fissure. The right hemisphere was placed on its medial surface and the dorsal surface of the cortex was removed at an angle that best preserves the integrity of the CA1 hippocampal subregion, the so-called ‘magic cut’ (blade tangential to the dorsal surface and 10° acute to the sagittal plane) (*61*). The right hemisphere was mounted on the freshly cut dorsal surface to a vibrating microtome stage (Leica VT1200S, Leica Microsystems) that was coated with a shallow strip (~4 cm x 2 cm) of cyanoacrylate glue. The stage was then placed into the slicing chamber, submerged in sucrose-ACSF, and oriented so that the lateral aspect of the brain faced the vibratome blade. 400 µm brain slices were prepared at a horizontal oscillation amplitude of 1.2 mm and a forward velocity of 0.05 $mm.s^{-1}$. Cut slices were transferred individually using a disposable wide mouth Pasteur

pipette into a submerged brain slice holding chamber that contained carbogenated sucrose ACSF maintained at 33°C. After 30 minutes the holding chamber was removed from the water bath and stored at room temperature until needed. Individual slices were transferred to a submerged recording chamber that was continually perfused with room temperature, carbogenated ACSF containing (in mM): 125 NaCl, 2.5 KCl, 25 $NaHCO_3$, 1.25 NaH_2PO_4, 25 glucose, 1 $MgCl_2$, 2 $CaCl_2$). The ACSF flow rate was 6-8 $mL.min^{-1}$ and slices were held in place with a 'harp' fabricated from an O-shaped platinum wire strung with parallel nylon threads. CA1 pyramidal neurons were visualized using an upright microscope (Scientifica SliceScope) equipped with infrared differential interference contrast illumination and a water immersion objective (Olympus XLUMPLFLN water-immersion objective, 1.00 NA). Fluorescently labelled neurons were identified under epifluorescence using a metal-halide lamp epifluorescence illumination system (X-cite 120Q) and GFP filter sets (Chroma), and a CMOS camera (Hamamatsu C11440-36U ORCA-spark). Whole cell patch-clamp recordings from GFP-positive CA1 pyramidal neurons were performed using thin-walled borosilicate glass pipettes (1.5 mm OD, 1.17 mm ID; Harvard Apparatus). Pipettes had a tip resistance of 2-3 MΩ when filled with a K-gluconate based internal solution (in mM 142 K-gluconate, 4 KCl, 0.5 EGTA, 10 HEPES, 2 $MgCl_2$, 2 Na_2ATP, 0.3 Na_2GTP, 1 Na_2Phosphocreatine, pH = 7.25, 285-295 mOsm). A calculated +14 mV liquid junction potential was left uncorrected. Electrophysiological recordings were made with a Multiclamp 700B amplifier (Molecular Devices), filtered online at 10 kHz with the built-in 4-pole Bessel Filter, and digitized at 62.5 kHz (NI-6221, National Instruments) using WinWCP software (courtesy of John Dempster, University of Strathclyde, Glasgow, UK). Bridge balance was applied. Neuronal excitability was assessed from the input-output relationship of

the neuron. Action potentials were elicited using square-wave depolarizing current steps (25 pA steps, 500 ms; 0 – 525 pA) from a holding potential of -60 mV and then from RMP. If no action potentials were observed using this protocol, 50 pA steps (500 ms; 0 – 1 nA) were applied from a holding potential of -60 mV to try to obtain the action potential current threshold. The number of action potentials during each 500 ms step was obtained using the threshold (0mV) event detection function in Clampfit. Membrane resistance (Rm) and capacitance (Cm) values were obtained in current-clamp mode from holding potential of -60 mV. Hyperpolarising current steps (-10 pA steps, 50 ms) were applied and five of the resultant event-free voltage transients were averaged. Rm was calculated according to Ohm's law from the amplitude of the average voltage transient. Cm was calculated according to τ = RmCm; τ was obtained by fitting a monoexponential curve to the rising phase of the average voltage transient.

Action potential waveform analysis. Action potential (AP) kinetics were analyzed for the first AP that was elicited from a holding potential of -60 mV using a custom MATLAB script. AP threshold was defined as the first point at which $dV/dt > 20$ mV/ms. Maximal rising and repolarizing slopes were also obtained from the first derivative. AP amplitude and AHP amplitude (the minimum point occurring 5ms after AP peak) were measured relative to AP threshold. AP half-width was the time window between the AP reaching the half-maximal amplitude on the upstroke and downstroke.

Acute pilocarpine in visual cortex

1.5ul AAV *cfos*-dsGFP or AAV CamKII-dsGFP (*15*) was injected into all the V1 cortical layers (DV: 0.7/0.5/0.3). A cannula for guided substrate injection were

implanted at the same coordinate after the viruses were injected. Mice were returned to the home cage for post-surgery recovery for two weeks before induction of acute seizures by pilocarpine. Pilocarpine (3M in saline) was injected 0.5 mm below the cannula (DV = -1.0) with a Hamilton 5 µl syringe in a volume of 300 nl. The mice were observed for 2 hours post-pilocarpine injection to evaluate seizure severity. The brains were then extracted and fixed with PFA for immunohistochemistry.

Acute pentylenetetrazole (PTZ)

Male and female mice wild-type C57BL/6J mice (3 months old) were placed in a stereotaxic frame and injected with 1.5 µl AAV of *cfos*-GFP/*cfos*-EKC into bilateral ventral hippocampi (AP: 3 mm, ML: 2/3 bregma-lambda, DV = 3.5/3/2.5) with 500 nl at each depth. Two weeks after the virus injection, mice were subjected to PTZ seizure induction. A single dose of PTZ (Sigma-Aldrich) dissolved in saline was administered intraperitoneally at 55mg/kg. The mice were observed closely for the first 30 minutes and their behavior was scored every 5 minutes according to the Racine scale (*36*). The latency to seizure onset was recorded. The mice were further monitored for 2 hours until they had completely returned to normal and then they were returned to the home cages. The PTZ-seizure induction was repeated, with the same dose and observation period, at two time-points: 24 hours and 14 days after the initial PTZ administration. The researcher who performed the experiments was blinded to the virus injected.

Scores: 1. sudden behaviour arrest; 2. facial jerking; 3. neck jerks; 4. clonic seizure or sitting; 5. tonic-clonic seizures (lying on belly); 6. tonic-clonic seizure (lying on side) or wild jumping; 7. tonic extension, possibly leading to respiratory arrest and death (*36*).

Intra-amygdala kainic acid model of chronic epilepsy

Male wild-type C57BL/6J mice (9-12 weeks old) were injected with kainic acid (0.3µg, 10mg/ml, Tocris) in the right amygdala (AP: 0.94, ML: +2.85, DV: 3.75) using a microinjection pump (WPI Ltd.). The injection volume was 200nl at 200 nl/min using a Hamilton Syringe (900 series, 5 µl, 33 gauge flat-end needle, EssLab Ltd.), under isoflurane anesthesia (typical surgery time 10-15 min). The mice were closely monitored during status epilepticus (SE) immediately after the kainic acid administration. SE was terminated with intraperitoneal injection of diazepam (10mg/kg) no more than 40 minutes after kainic acid injection. Only animals that exhibited seizure episodes above stage 4 were carried into the subsequent studies. An electrocorticogram (ECoG) transmitter (single channel, 256 Hz, A3028C-CC, Open Source Instruments, Inc.) was implanted subcutaneously two weeks post-kainic acid injection; the recording electrode was placed above the right ventral hippocampus (AP: -2/3 of bregma/lambda, ML: +3 mm). At the same coordinate, a cannula was implanted for subsequent guided injection. An identical cannula was placed above the left ventral hippocampus. The ground electrode of the transmitter was placed in the posterior region of the left hemisphere. The mice were then returned to their home cage and kept single housed for the remainder of the study. After two weeks of ECoG data acquisition, we performed bilateral hippocampal virus injections. 1.5µl AAV9 *cfos*-dsGFP, *cfos*-EKC or *cfos-KCNJ2* was injected bilaterally via cannulas into the ventral hippocampus (DV = 3.5/3/2.5 mm, cannula length = 7.05) under isoflurane anesthesia. The mice were returned to home cages for

recovery. Two weeks later, transmitters were turned back on, in some cases with video recordings. Animals were sacrificed after the experiments for brain collection.

Intra-hippocampal kainic acid model

Following a 2 week recovery period after viral injection, mice were briefly anaesthetized and kainic acid (KA) injected into the dorsal hippocampus to induce status epilepticus. The injection needle was inserted to a depth of -2.0mm relative to the dura. 50 nl KA (20mM) was injected into the dorsal hippocampus at a rate of 50 nl/minute. The needle was left in place for 5 minutes, withdrawn, and the cannula cap resealed. Successful induction of status epilepticus was confirmed via monitoring of animal behaviour. Status epilepticus was allowed to continue until spontaneous seizure termination. We have recorded EEG this model and the first seizure appears at 13.2±2.2 (mean ± SD) with interictal spikes present in the hippocampus after SE (recorded with depth hippocampal electrodes).

ECoG data acquisition and PyEcoG event detection

The wireless ECoG data acquisition was previously described (*14*). A list of features were extracted from the recordings, from which coastline and power spectrum across different frequencies (1-120Hz) were calculated.

Interictal spike analysis

Interictal spikes were detected by locating peaks with an amplitude exceeding 6x the standard deviation of the recording. Seizures were manually excluded from the analysis and large amplitude artefacts were filtered from true interictal spikes by analysis of parameters derived from a library of both true positive and false positive

events, constructed by visual inspection of a random sample of 750 identified events. Spike half-width, maximal amplitude, maximal slope, peak power frequency, and kurtosis were calculated for each spike and the 90% confidence interval of the distribution of true positive and false positive parameter values used to filter the total dataset. Filter values were adjusted empirically. Spike frequency and amplitude were analyzed for each mouse during both baseline and post-treatment phases of recording.

Behavior assessments

All behavioral tests were performed in age-matched and littermate mice 1 hour after starting the light phase and 1 hour before starting the dark phase of their daily day/night cycle. When possible, the tests were performed under red light to reduce anxiety.

Contextual Fear Conditioning (CFC). The fear conditioning was carried out in the Near Infrared (NIR) Video Fear Conditioning package by Med Associates Inc. A glass chamber with a metal grid was placed inside a sound proof chamber. Near infrared (NIR) light was used for recording under dark conditions. Mice were randomized into two groups, each injected bilaterally with either *cfos*-dsGFP or *cfos*-EKC. Mice were housed in their home cage for at least 3 weeks before CFC was assessed. Mice were handled for 7 days before experiment, until signs of fear or anxiety (jumping, freezing, excretion) were extinguished. On the day of the experiment, mice were kept in the habituation room for 1 hour and were only introduced to the behavior room where the CFC apparatus was located when they were due to take the test. The CFC experiment consisted of three phases. On day one, the mice received harmless electrical foot shocks. The electrical stimuli (0.6

mA) were delivered by a metal grid via manual control. Mice were placed in the chamber for 2 minutes of habituation, then three-foot shocks were delivered for 2s each, with ITI (inter-trial-intervals) of 60 seconds. The test was performed in a dark environment and the mice were recorded with the NIR camera. The grid floor was cleaned with 70% EtOH after testing each mouse. Different grid floors were used for males and females and males were always tested before females. The mice were then returned to the home cage. 24 hours after the foot shock, mice were returned to the same chamber with the same conditions, for assessment of fear recall. The mice were placed in the chamber for 5 minutes. No foot shock was delivered. After 1 hour, in which the mice were back in the home cage, the chamber was modified by replacing the grid floor with a hard plastic board, and the walls were covered with colored paper. We also placed a filter paper in the chamber with a drop of almond essential oil. The mice were placed in the ‘novel’ context for 5 minutes. The freezing behavior of the mice was counted manually by an experimenter blinded to the treatment given.

Open field. Open field behavior was tested in a white open arena of dimension 50x50x50cm. Mice had no prior exposure to the behavior room or to the apparatus. The mice were habituated for 30 minutes before being introduced into the behavior room. The test was performed under red light to reduce anxiety. At the start of the experiment, mice were gently placed in the center of the arena. The test lasted 30 minutes with the experimenter not present in the same room. The apparatus was cleaned with 70% EtOH between animals. The mice were recorded with an overhead Raspberry Pi4 camera (XL-RB-AluP4+07FAN) and VideoArchiver software. The animal tracking and data analysis were carried out semi-automatically using a script written in Bonsai (Open Ephys, https://open-ephys.org/bonsai). In brief, the outline

of the animal was extracted and its body centroid was calculated. The software generated a location for the center of the mice as an x, y coordinate every 30 frames of the recording. The tracked locations were then used to analyze parameters including thigmotaxis and travel distance using an in-house Python script.

Spontaneous T-maze alternations. Mice were returned to the same behavior room 24 hours after the Open field test assessment for T-maze alternations. This test was also performed under red light. The apparatus was specially made with transparent Pyrex materials. The apparatus was cleaned with water after each animal had finished their test. The walls of the apparatus were 20cm high. Each of the arms was 25cm long, and the running track was 50 cm long. A central partition 10 cm long divided the end of the track into two parts facing the two arms. The protocol for T-maze alternation was adapted from (*62*). The mice were habituated to the room for 5-10 minutes before the experiment start. They were then placed in the start area at the base of the T, facing away from the track. The mice were handled gently using a plastic tube to which they were habituated during the handling sessions prior to the experiment. The animals ran spontaneously towards the top of the T and entered one arm. An entry was scored once the whole body of the mouse has entered the arm. A guillotine door was then lowered to trap the mouse in the arm for 30 seconds. Mice were then transported back to the start area, and allowed to run down the track again with all doors open and without the separation wall in the middle. The arm of the T maze entered on the second trial was recorded as either the same or different from the first entry, and the mouse was again left confined to the arm for 30 seconds. Mice were then immediately returned to the start area with no delay for the next trial. For each mouse, 10 consecutive trials were performed. In any of the trials, if the mice failed to leave the start area after 90s, they were taken out of the maze for a

short break of 10 minutes and tried again. If the mice repeatedly failed to run, they were temporarily suspended from the test, and another trial was conducted on the next day. If the animal still failed to initiate directional movements, they were eliminated from the dataset.

Olfactory Discrimination. Mice were subjected to the olfactory discrimination test with at least a 48hour interval after the T-Maze test. The olfactory stimuli were prepared by diluting essential oils of peppermint, almond and raspberry in water (1:20), and used to dip clean cotton swabs. Both habituation and test were performed under white light. The mice were habituated for 30 minutes to an empty individually ventilated cage without bedding and a clean cotton swab inserted through the drinking bottle hole to remove any novelty confounder represented by the cotton swap tip. After that, they were introduced to the scented cotton swaps, inserted at the same height as the mouse head. Each smell was presented three times, for 2 minutes each trial with an ITI of approximately 1 minute (the time it took the experimenter to change the cotton swab). The order of the scents was water, peppermint and almond and the animals were expected to show habituation to each smell across the three trials and dishabituation when faced with a different smell. The exploration of the scented cotton swap tip was quantified as the seconds spent sniffing it, scored on-site by an experimenter blinded to the treatment given. The animals were tested twice for Olfactory Discrimination post-virus injection. The first test involved the same smells previously used. The second test involved changing almond for raspberry smell, to control for olfactory memory as a confounder for olfactory discrimination.

Immunofluorescence

Primary neuronal cultures. Primary cortical neurons in cultures were treated with 100μM 4-aminopyridine (4-AP) and 30μM picrotoxin (PTX) or equal volumes of water/DMSO as control on 18-21DIV. At 2, 6, 24, and 48 hours post-drug treatment, neurons were fixed with 4% PFA at room temperature for 30 minutes. The coverslips were then washed with PBS, followed by membrane permeabilization and non-specific binding block in PBS-T-BSA buffer (PBS plus 0.1% - 0.3% Triton, 1-3% BSA and 10% goat serum for 30 minutes. The blocking solution was replaced with PBS-T buffer supplemented with polyclonal rabbit *cfos* antibody (Synaptic systems, #226003) or chicken Turbo-GFP (Origene, #TA150075) with gentle rocking at 4°C overnight. The cells were washed with PBS-T to remove residual primary antibody, followed by incubation with secondary antibody Alexa 568 (goat-anti rabbit, 1:1000) or Alexa 488 (goat-anti chicken, 1:1000) for 1 hour. Cells were then briefly incubated in Hoechst solution (1:10000 diluted in PBS) for 30 minutes for nuclear staining or mounted directly onto glass slides with Vectorshield (with DAPI, Vector Laboratories #H-1200), or directly mounted with Fluoroshield supplemented with DAPI (D9452, Sigma). The cells stained with Hoechst solution were subsequently mounted with clear Fluor shield (Abcam).

PFA-fixed brain sections. Immunostaining was performed on 60um free-floating mouse brain PFA sections. Slices were blocked and permeabilized for 3 hours in PBS supplemented with 5% goat serum, 3% BSA and 0.3% triton-X before being labelled with the following antibodies overnight: rabbit anti-GABA (A2052, Sigma), mouse anti-GAD (MAB5406, MerckMillipore), mouse anti-parvalbumin (P3088, Sigma) and chicken anti-NeuN (ABN91, MerckMillipore). Slices were washed three times in PBS and incubated with secondary antibodies at room temperature for 3 hours: Alexa Fluor® 568 goat anti-rabbit (A11036, Invitrogen), Alexa Fluor® 555 goat

anti-mouse (A32727, Invitrogen) and Alexa Fluor® goat anti-chicken 633 (A21103, Invitrogen). Slices were washed and mounted with Fluoroshield mounting medium with DAPI (ab104139, Abcam) before acquisition.

Image acquisition and analysis. Confocal images were acquired with Zeiss AxioScop A1 polarized light microscope and/or an inverted confocal laser scanning microscope (LSM 800, ZEN 2009, Zeiss). Cell counting *in vitro*: analyses was performed using ImageJ 1.52q (Wayne Rasband, National Institute of Health). All the images were acquired with identical laser settings and digital gain values. This was processed separately for DAPI and dsGFP channels and the dsGFP count was normalized to DAPI. Cell counting *in vivo*: all quantification was performed on ImageJ 1.52q. For interneuron analysis tile scans were acquired of the entire hippocampus and each marker was quantified for dsGFP co-localization. For excitatory neurons analysis, 4-8 micrographs were acquired at various positions across the specified hippocampal region and $NeuN^+$, $GABA^+$ and GFP^+ cells were quantified. Total excitatory neuron number was estimated by subtracting $GABA^+$ cells from $NeuN^+$ cells.

Human Cortical Assembloids

Culture of control hiPS cells. An hiPSCs line that has been previously described (*63*) was maintained in human recombinant vitronectin (Life Technologies), diluted 1:100 in PBS, and Essential 8 medium (Life Technologies). hiPSCs were routinely passaged using ethylenediaminetetraacetic acid (EDTA), 0.02% solution (Sigma-Aldrich) when reaching 90% confluency.

Generation and assembly of hCS and hSS. hiPS cells were harvested using Accumax™ (Sigma), counted, resuspended in Essential 8 medium supplemented with ROCK-inhibitor (StemCell technology) and seeded at 3,000,000 cells/well of an

Aggrewell^{TM} 800 (StemCell Technologies), corresponding to 10,000 cells/microwell, to form embryoid bodies (EBs). After 24h, EBs were dislodged from the microwells and transferred to ultralow-attachment 10cm dishes (Corning) in Essential 6 medium (Life Technologies) supplemented with SMAD inhibitors dorsomorphin (5 µM, Sigma-Aldrich) and SB-431542 (10 µM, Tocris). No media change was performed the next day, and media was then changed every day for 4 days. On day six, neural spheroids were transferred to neural medium containing Neurobasal A (Life Technologies), B-27 supplement without vitamin A (1:50, Life Technologies), GlutaMax (1:100, Life Technologies), penicillin and streptomycin (1:100, Life Technologies) and supplemented with the growth factors EGF (20 ng/ml; Bio Techne) and FGF2 (20 ng/ml; Sigma). Media was then changed every day from day 6 to day 15 and every 2 days from day 15 to day 24. For the generation of hSSs, the medium was supplemented with additional small molecules during the first 23 days in culture: Wnt pathway inhibitor IWP-2 (inhibitor of WNT production-2, 5 µM, Selleckchem) from day 4 until day 24; Shh pathway agonist SAG (smoothened agonist, 100 nM, Selleckchem) from day 12 to day 24. From day 25 to 42, neural medium for both hCSs and hSSs was supplemented with the growth factors BDNF (20 ng/ml, Peprotech) and NT3 (20 ng/ml, Peprotech) and changed every other day. hCSs and hSSs were maintained in neural medium without growth factors with medium changes every 3-4 days from day 43 onwards. To generate assembloids, day 60-65 hCSs and hSSs were fused together by placing one hCS and one hSS in a 1.5ml Eppendorf tube with 1ml of media for 48-72h with a media change after 24h.

Induction of neural activity in assembloids. Assembloids were incubated for 4h in 50µM 4-AP + 100µM PTX or in maintenance neural media (control). Samples were

then washed with PBS, fixed in 4% PFA overnight and processed for immunofluorescence.

Induction of neural activity in assembloids with 4AP and PTX. Whole assembloids were plated onto glass coverslips using Geltrex™ (ThermoFisher) prior to recording. Assembloids plated on coverslips were perfused at a fast flow rate (8-10 $ml.min^{-1}$) with artificial cerebrosbinal fluid (aCSF) containing: 126 mM NaCl, 26 mM $NaHCO_3$, 10 mM Glucose, 5 mM KCl, 1.25 mM NaH_2PO_4, 2 mM $CaCl_2$, 1.2 mM $MgCl_2$ that was saturated with 95% 0_2 and 5%CO_2 (pH 7.4), and maintained at 34-36 °C with an in-line heater. Local field potential (LFP) recordings were performed using thin-walled borosilicate glass pipettes (1.5 mm OD, 1.17 mm ID; Harvard Apparatus) that had a tip resistance of ~1 MΩ when filled with aCSF. Signals were recorded using a Multiclamp 700B amplifier (Molecular Devices) with the gain set to 50, high (1 Hz) and low pass (3 kHz) filtered, and digitized at 5 kHz (BNC-2090A, NI-6221, National Instruments) using WinEDR software (courtesy of John Dempster, University of Strathclyde, Glasgow, UK). Following baseline LFP recordings, 4-AP (in ddH_20) + 100μM PTX (in DMSO) were added to the aCSF to give a final concentration of 50μM and 100μM, respectively.

Measuring the efficacy of cfos-EKC in assembloids. Hyperactivity was induced in assembloids by incubating them for 1hr 45 mins in their culture media supplemented with 55 mM KCl (2M stock in dH20). After this, the KCl-supplemented media was replaced with fresh culture media and the assembloids were returned to the incubator for 2 hrs 15 mins. LFP recordings were conducted for 6hrs after this induction protocol. 15 mins prior to recording, assembloids were plated onto glass coverslips using Geltrex™(ThermoFisher). For recordings, the plated assembloids were perfused at a fast flow rate (8-10 $ml.min^{-1}$) with aCSF containing (in mM): 125

NaCl, 2.5 KCl, 25 $NaHCO_3$, 1.25 NaH_2PO_4, 25 glucose, 1 $MgCl_2$, 2 $CaCl_2$ that was saturated with 95% 0_2 and 5%CO_2 (pH 7.4), and maintained at 34-36 °C with an in-line heater. LFP recordings were performed using thin-walled borosilicate glass pipettes (1.5 mm OD, 1.17 mm ID; Harvard Apparatus) that had a tip resistance of ~1 MΩ when filled with aCSF. LFP signals were recorded using a Multiclamp 700B amplifier (Molecular Devices) with the gain set to 50, high- (3 Hz) and low-pass (3 kHz) filtered, and digitized at 10 kHz (BNC-2090A, NI-6221, National Instruments) using WinEDR software (courtesy of John Dempster, University of Strathclyde, Glasgow, UK). Assembloids were inspected under differential interference contrast illumination and the glass electrode was inserted into the hCS, 30 to 60 microns below the surface, in a neuron-rich area close to the hCS-hSS junction. Successful penetration of the geltrex and organoid was indicated by an increase in the SD of the LFP signal. A baseline of 450-600 seconds was recorded before KCl (2M stock in dH20) was added to the aCSF to bring the concentration of K^+ to 55 mM. The addition of KCl typically resulted in a progressive increase in neuronal activity over the first 10 minutes, as indicated by an increase in LFP SD. If no increase in activity was observed over the first 10 minutes the position of the pipette was moved to a new position within the same region to find a focus of elevated activity, if there was no increase in activity detected after 15 minutes the recording was discarded. Otherwise, LFPs were recorded for a total of 6000 seconds. We used the standard deviation of the LFP as a measure of excitability of the assembloid. The standard deviation was sampled over 200 ms at 5 second intervals using Clampfit. The median SD over 500 seconds was used to assess changes in activity after the addition of KCl.

Viral labelling of spheroids and assembloids. To specifically label hSS-derived neurons, 8-10 days before assembly hSSs were placed into a well of an ultra-low attachment 24-well plate in 400 μl media plus 10 μl lentivirus suspension (LV-mdlx5/6-GFP) and incubated for 48h. For transduction with AAV9-*cfos*-dsGFP, assembloids were placed into a well of an ultra-low attachment 24-well plate in 400 μl media plus 20 μl AAV9-*cfos*-dsGFP. 500 μl of media was added the next day and a half media change was performed after 48h. Routine media changes were performed afterwards. Transduced spheroids were analyzed for transgene expression 11 days after AAV infection.

Functional titers for the *cfos*-dsGFP and *cfos*-dsGFP-EKC lentiviruses were both found to be 1.7 x10^9 IFU/mL using the Lenti-X^{TM} Provirus Quantitation kit (Takara Bio, Cat 631239) according to the manufacturer's protocol, with the following amendment: the calculated provirus copy number per cell was scaled to account for the HEK cell hypotriploid genome.

Cryopreservation. Spheroids at day 25 and day 55 were placed in 1.5ml Eppendorf tubes, washed with 1x PBS and fixed with 4% PFA from 2h to overnight at 4°C. They were then washed with 1x PBS and placed in a 30% sucrose solution for 1-2 days, washed with 1x PBS and embedded in cryogenic molds using O.C.T. compound (VWR). The embedded samples were then snap-frozen on dry ice and stored at -80°C. 10 to 20 μm thick sections were cut using a cryostat (Bright Instruments) and sections were stored at -80°C.

Immunostaining of cryopreserved slices. Cryosections were washed with 1x PBS to remove excess O.C.T. and blocked using blocking solution (10% fetal bovine serum (FBS) and 0.3% Triton X-100 (Sigma) diluted in 1x PBS) for 1h at room temperature (RT). The sections were then incubated overnight at 4°C with primary antibodies

diluted in blocking solution. The primary antibodies used are listed in Table 1. Sections were then washed in 1X PBS and incubated with fluorochrome-labeled secondary antibodies diluted in blocking solution for 45mn at room temperature. Secondary antibodies used were as follows: Alexa Fluor® 488 Goat Anti-mouse IgG (1:400; Alexa Fluor, Life Technologies), Alexa Fluor® 594 Goat Anti-rabbit (1:400; Alexa Fluor, Life Technologies), Alexa Fluor® 488 Goat Anti-rabbit (1:400; Alexa Fluor, Life Technologies). Sections were mounted using ProLong™ Gold Antifade (Life Technologies) and images were acquired with ZEN software (Zeiss) on a LSM710 confocal microscope (Zeiss).

Immunostaining of free-floating slices. Assembloids were placed in 1.5ml Eppendorf tubes, washed with 1x PBS and fixed with 4% PFA overnight at 4°C. Fixed samples were then embedded in 4% low-melting point agarose (ThermoFisher) and 70μm slices were cut in PBS using a vibratome (VT1000 S, Leica). Immunostaining was performed on free-floating slices whereby blocking was performed in PBS supplemented with 0.3% Triton, 3% BSA and 5% goat serum gently rocking at room temperature for 3 hours. Primary antibodies dilutions were done in blocking solution diluted 4X in PBS and incubated overnight at 4°C. The primary antibodies used are listed in Table S1. Slices were washed in 1X PBS and incubated with fluorochrome-labeled secondary antibodies diluted in blocking solution for 3h at room temperature. Slices were mounted using ProLong™ Gold Antifade (Life Technologies) and images were acquired with ZEN software (Zeiss) on a LSM710 confocal microscope (Zeiss).

Table S1 – Primary antibodies used for immunofluorescence

**Antibody**	**Species, isotype**	**Dilution**	**Supplier**
**NESTIN**	Mouse, IgG1	1:1000	Sigma
**FOXG1**	Rabbit	1:300	BioLegend
**PAX6**	Rabbit	1:100	BioLegend
**NKX2.1** (TTF-1)	Mouse, IgG1	1:50	Sigma
**MAP2**	Mouse, IgG1	1:400	Sigma
**NEUN**	Mouse, IgG1	1:100	Sigma
**CFOS**	Rabbit	1:200	Synaptic Systems
**TurboGFP**	Chicken, IgY	1:500	Origene
**GABA**	Rabbit	1:1000	Sigma

Statistical analysis

The statistical tests for significance are detailed in the figure legends. Data are plotted as box and whiskers, representing interquartile range (box), median (horizontal line), and max and min (whiskers), together with all the points. The mean is further shown as "+". Deviation from normal distributions was assessed using D'Agostino-Pearson's test, and the F-test was used to compare variances between two sample groups. Student's t-test (parametric) or Mann-Whitney (non-parametric) tests were used to compare two groups. Fisher's exact test were used for event occurrence frequency comparisons. One-sample t-test was used to compare normalized data. One-way ANOVA was used to compare multiple groups. To compare two groups at different time points we used two-way repeated measure ANOVA, followed by a Bonferroni post-hoc test for functional analysis. Three-way

ANOVA was used to compare data with three variables. Statistical analysis was carried out using Prism (GraphPad Software, Inc., CA, USA) and SPSS (IBM SPSS statistics, NY, USA).

Supplementary Text

Table S2. Properties of Immediate Early Gene Promoters

Promoter	Host gene	Main Binding domains	Earliest promoter activation post-stimulation	Return to baseline expression	Function	Reference
c-Fos	fos	CRE, SRE, SP1, SIE, AP-1	≈ 30 minutes (1-2 hours*)	6 hours (rats and mice)	Learning and memory; cell survival	*64, 65, 66*
Egr1	egr1/zif268	CRE, SRE, SP1	≈ 30 minutes (cell lines)	3 hours (cell lines)	Learning and memory	67, 68
mini-Arc	Arc/Arg3.1	TATA-box	peak at 2 hours	24 hours (neuronal culture)	Synaptic plasticity	29, 69, 70
E-SARE (synthetic)	Arc/Arg3.1	CRE, SRF/TCF, MEF2	peak at 2 hours	24 hours (neuronal culture)	Synaptic plasticity	69, 70
NRAM (synthetic)	Npas4	NRE	1hr (Npas4)	24 hours (neuronal culture)	Excitatory-inhibitory homeostasis; neuroprotection	31, 71, 72, 73

*Peak protein expression window

Parameters of neuronal activity

We observed modified parameters of neuronal activity in CA1 pyramidal neurons transduction with EKC or *KCNJ2* placed under the control of activity-dependent promoters. These changes are consistent with overexpression of Kv1.1 and Kir2.1 channels. Kv1.1 channel overexpression led to a decrease in firing rate, an increase in action potential current threshold and a larger afterhyperpolarization. Conversely, overexpression of Kir2.1 resulted in a net hyperpolarization of the resting membrane potential without affecting any other parameters. These effects on neuronal activity were largely consistent regardless of which IEG promoter was driving expression. The small differences between the promoters can be ascribed to either the different time course of activation (see Table S2) or the degree of activation reached by each

promoter. We hypothesise that the antiepileptic effect of the *cfos*-EKC construct is due to the multiple mechanisms of action of this potassium channel that regulates both intrinsic and synaptic excitability, and potentially contributes to an homeostatic rearrangement of the network (*74, 75*) On the other hand, the inability of *cfos-KCNJ2* to decrease seizures, even with hyperpolarisation of the RMP, may be due to a short half-life of this potassium channel in the cell membrane (12-24hrs), which is also affected by its own expression levels (*76, 77*).

Supplementary Figures

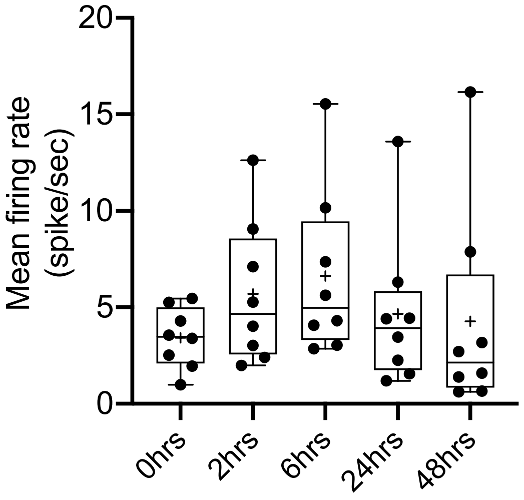

Fig. S1. Network mean firing rate following disinhibition with PTX application.

All data points are shown. Statistics in **Fig.1**. n=8 in 2 independent repeats.

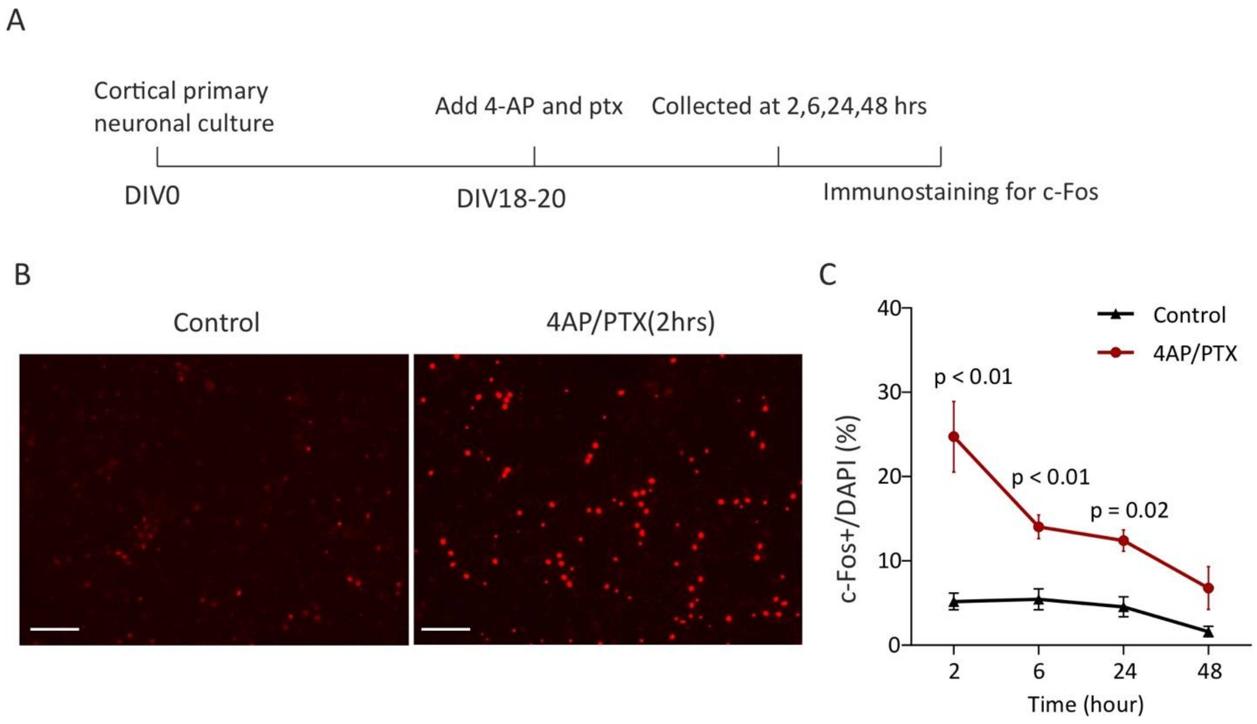

Fig. S2. In vitro characterization of the activity-dependent activation of cfos. **A.** Experimental timeline. B. Representative images of c-Fos staining (red) in control and 2 hours post 4AP/PTX treatment (scale bar = 100 μm). C. Quantitative analysis of the percentage of c-Fos positive compared to DAPI positive cells at 2, 6, 24 and 48hours after the 4AP/PTX treatments (n = 3, 9, 7, 2). Control groups were treated with saline (n = 5, 5, 6, 3). 2-way ANOVA followed by Bonferroni multiple-comparison test.

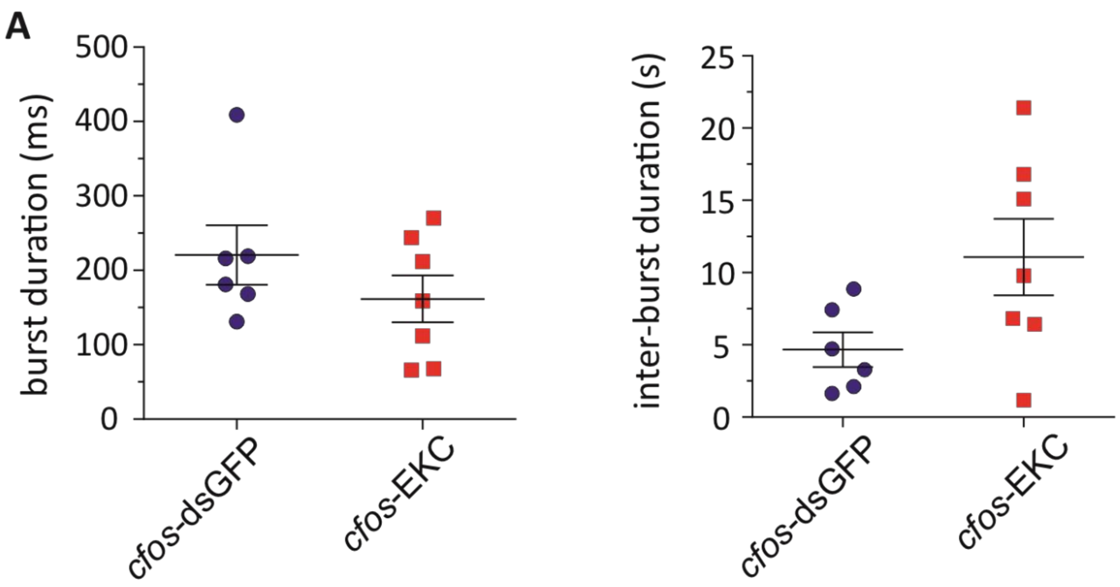

Fig. S3. Burst and inter-burst duration were not significantly different in cultures treated with either *cfos*-dsGFP or *cfos*-EKC.

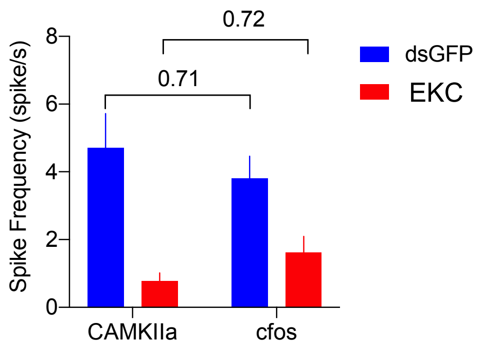

Fig. S4. Constitutive and Activity-dependent EKC effect in vitro. Primary cultures were transduced with either cfos-dsGFP or cfos-EKC or CAMKIIa-dsGFP or CAMKIIa-EKC. Graph represents the effect of different treatments on spike frequency. Two-way ANOVA.

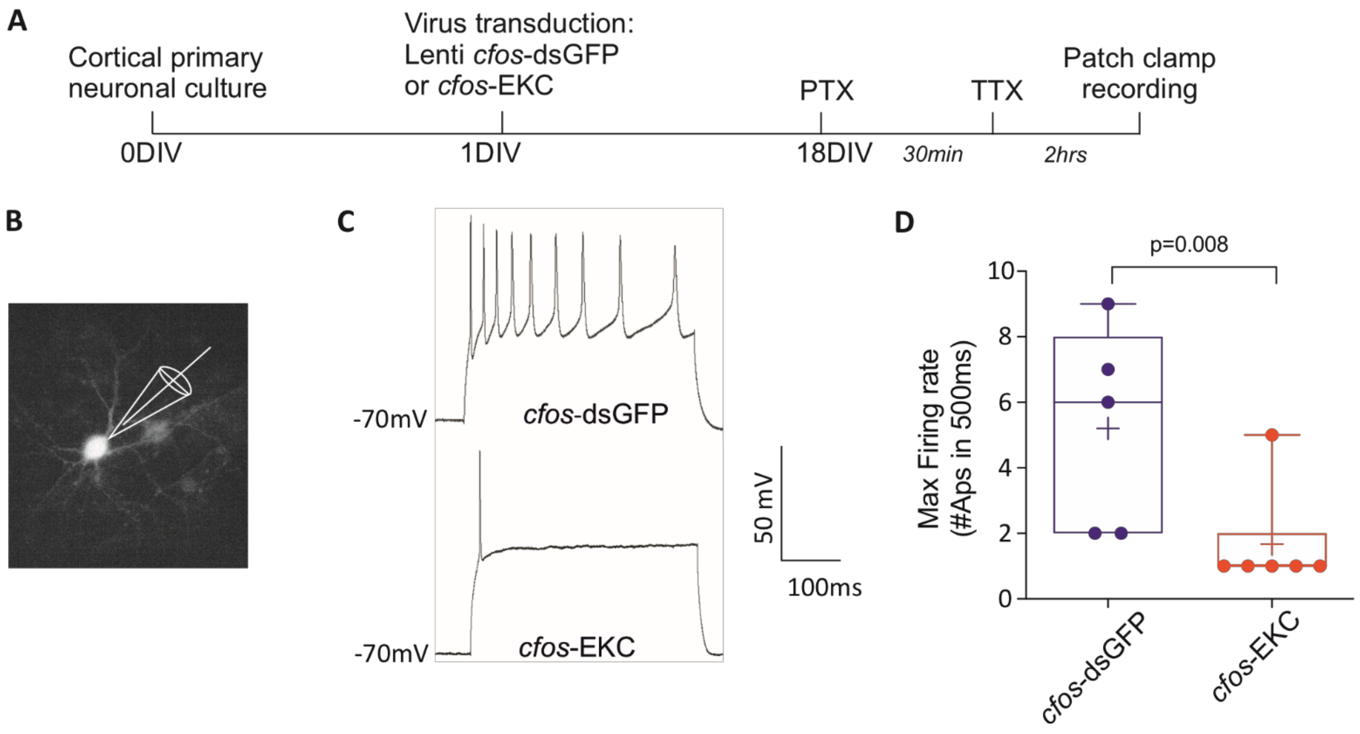

Fig. S5. Firing rate was reduced in *cfos*-EKC- compared to *cfos*-dsGFP-treated cultured neurons. A. Experimental timeline. **B.** Representative live imaging of a dsGFP positive neuron. **C.** Representative current clamp traces of action potentials elicited by a current step (250pA) from neurons transduced either with *cfos*-dsGFP or *cfos*-EKC. **D.** Box plot of the maximum number of action potentials triggered in *cfos*-dsGFP (n=5) compared to *cfos*-EKC (n=6). Mann-Whitney U test.

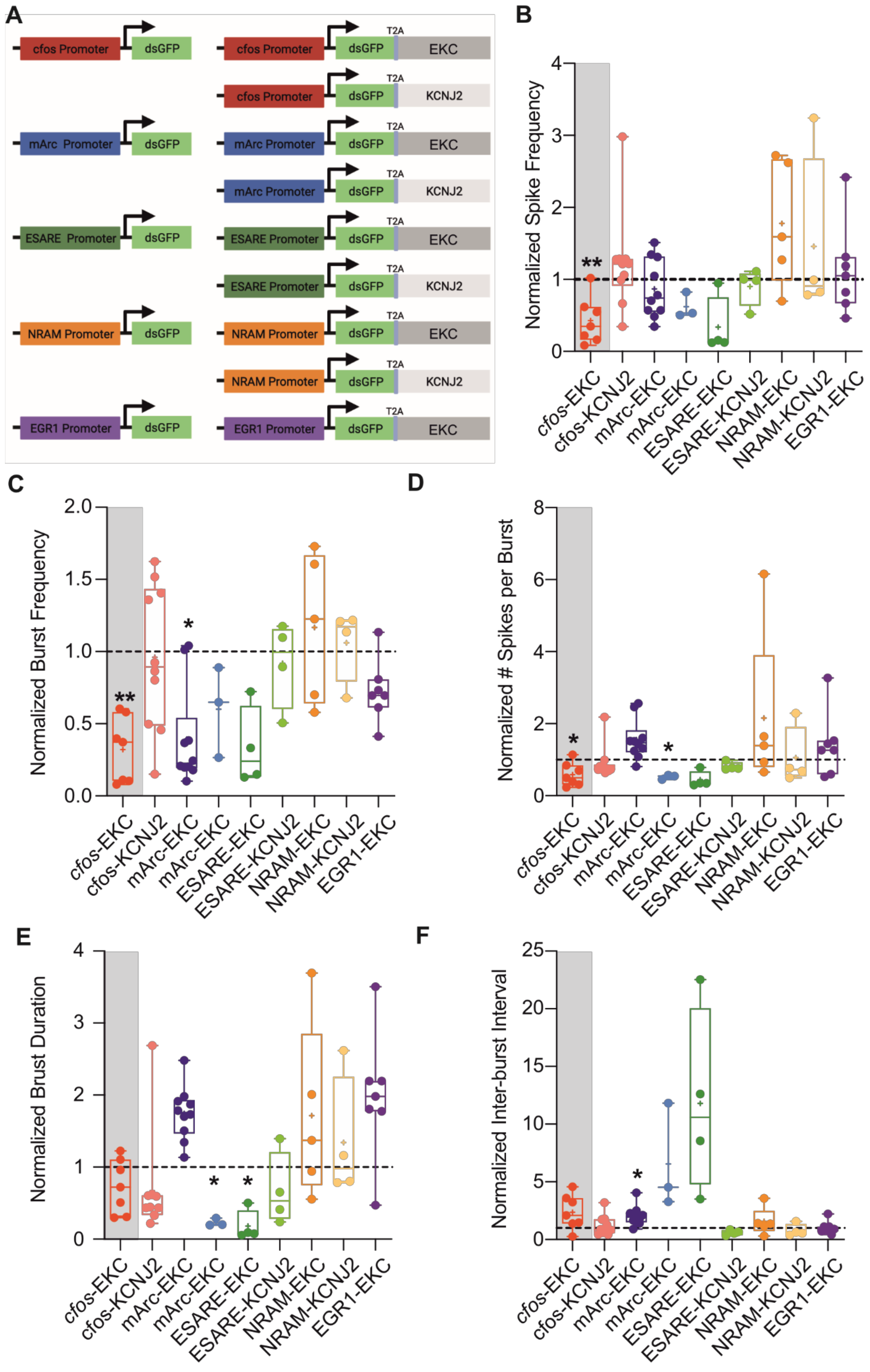

A
cfos Promoter
dsGFP
mArc Promoter
ESARE Promoter
NRAM Promoter
EGR1 Promoter
T2A
EKC
KCNJ2
B
Normalized Spike Frequency
C
Normalized Burst Frequency
D
Normalized # Spikes per Burst
E
Normalized Brust Duration
F
Normalized Inter-burst Interval
cfos-EKC
cfos-KCNJ2
mArc-EKC
mArc-EKC
ESARE-EKC
ESARE-KCNJ2
NRAM-EKC
NRAM-KCNJ2
EGR1-EKC

Fig. S6. *In vitro* activity-dependent gene therapy with different IEG promoters and transgenes*.* A. Primary cultures were transduced with either AAV9 *IEG*-dsGFP as control or AAV9 *IEG*-[EKC*/KCNJ2*]*.* **B-F.** Box-and-whisker plots showing the effect of *IEG*-[EKC*/KCNJ2*]*,* normalized to control, on spike frequency (B), mean bursting rate (C), average number of spikes per burst (D), average burst duration (E) and average inter-burst interval (F). * $p<0.006$, One sample t test vs 1, corrected for multiple comparisons, $\alpha=0.006$. Gray shading: data replotted from fig. 2D.

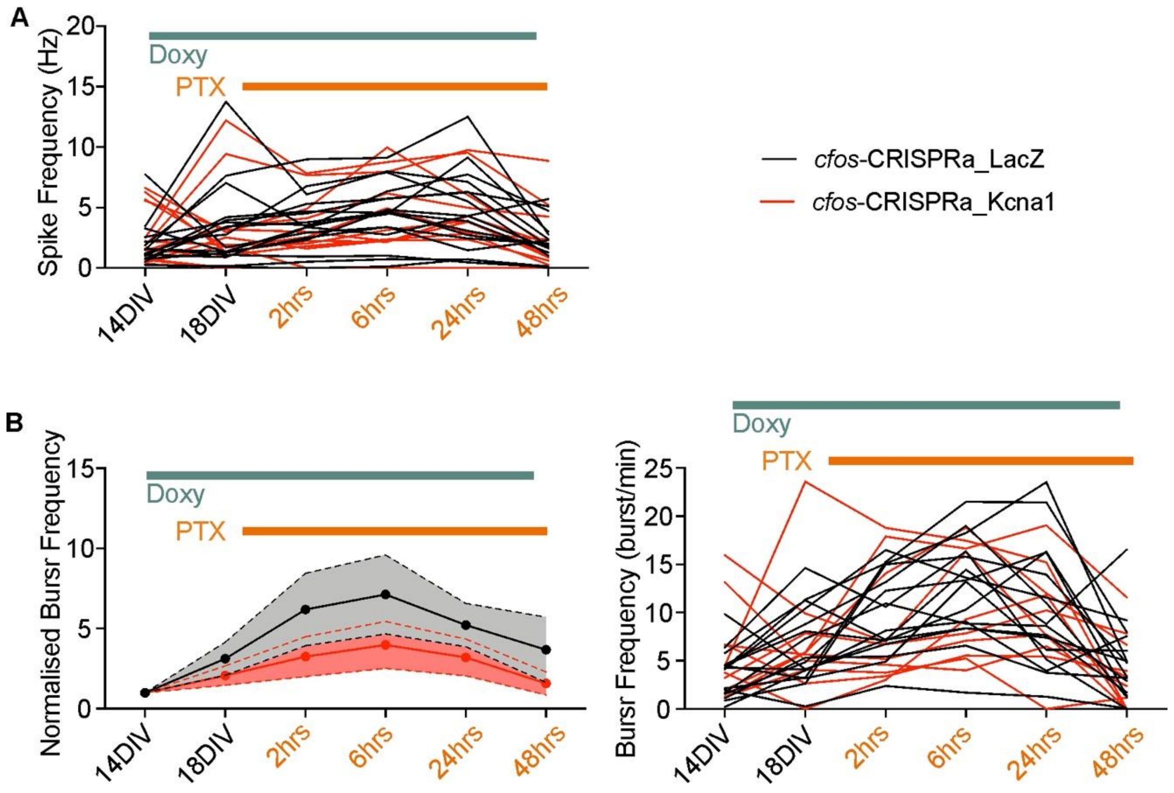

Fig. S7. Spike and Burst frequency in cultures treated with either *cfos*-CRISPRa_LacZ or *cfos*-CRISPRa_*KCNA1*. A. Data not normalized for spike frequency. B. Burst frequency for culture transduced with either *cfos*-CRISPRa_LacZ or *cfos*-CRISPRa_*KCNA1* normalized (left) or not normalized (right) to 14DIV recording.

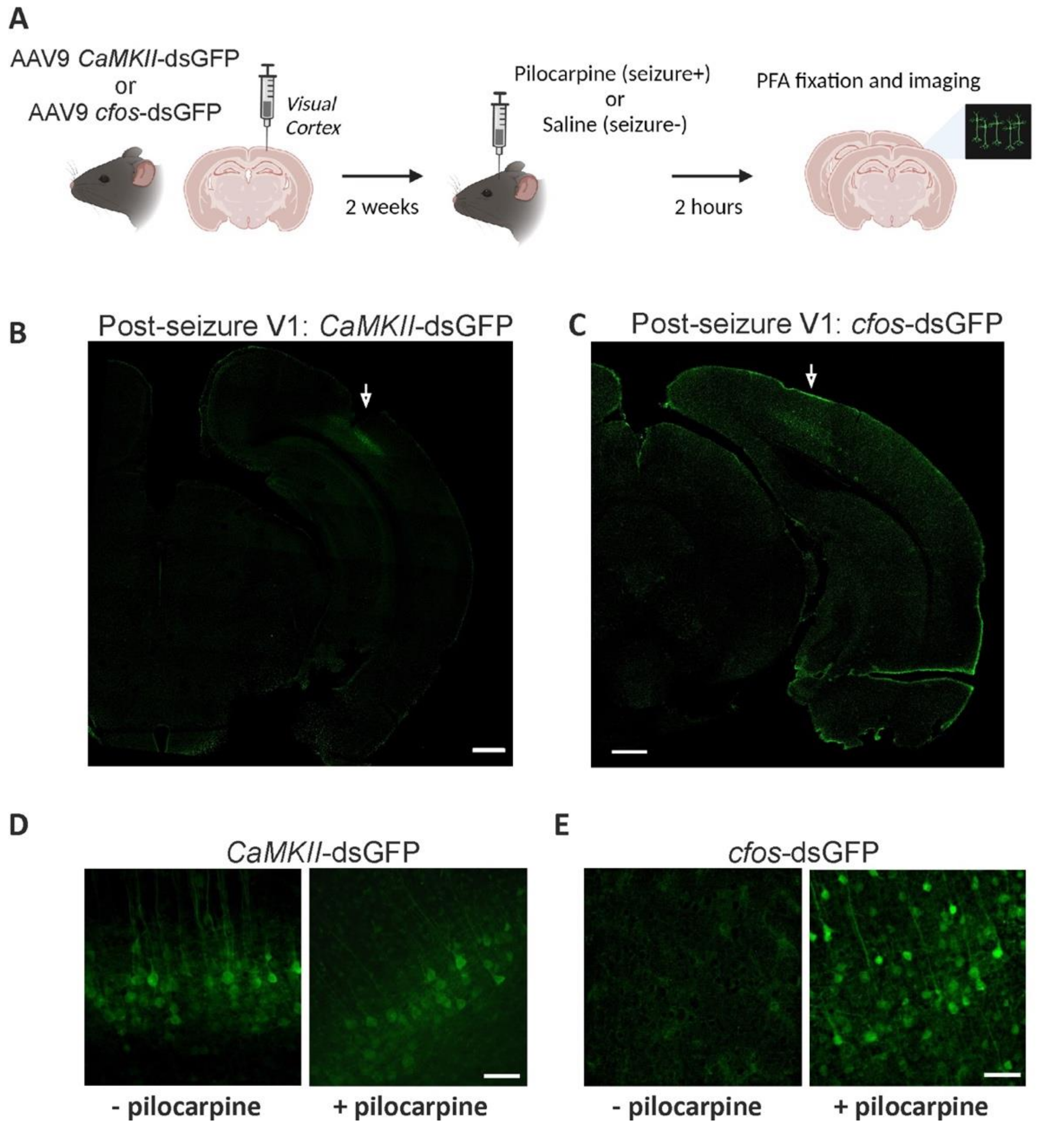

Fig. S8. *cfos*-driven transgene expression was activated by a chemoconvulsant *in vivo*. A. Experimental timeline. AAV9 *CaMKII*-dsGFP and *cfos*-dsGFP were injected in the visual cortex and acute seizures were induced by focal pilocarpine injection. **B, C.** Representative images showing dsGFP expression post-seizure for *CaMKII*-dsGFP (B) or *cfos*-dsGFP (C). dsGFP driven by *cfos* showed more localized expression. Scale bar = 500µm **D, E**. Zoomed in images of the visual cortex in animals injected with saline (D, E *left*) or with pilocarpine (D, E *right*). dsGFP driven by *cfos* was expressed only following pilocarpine injection. Scale bar: 50 µm.

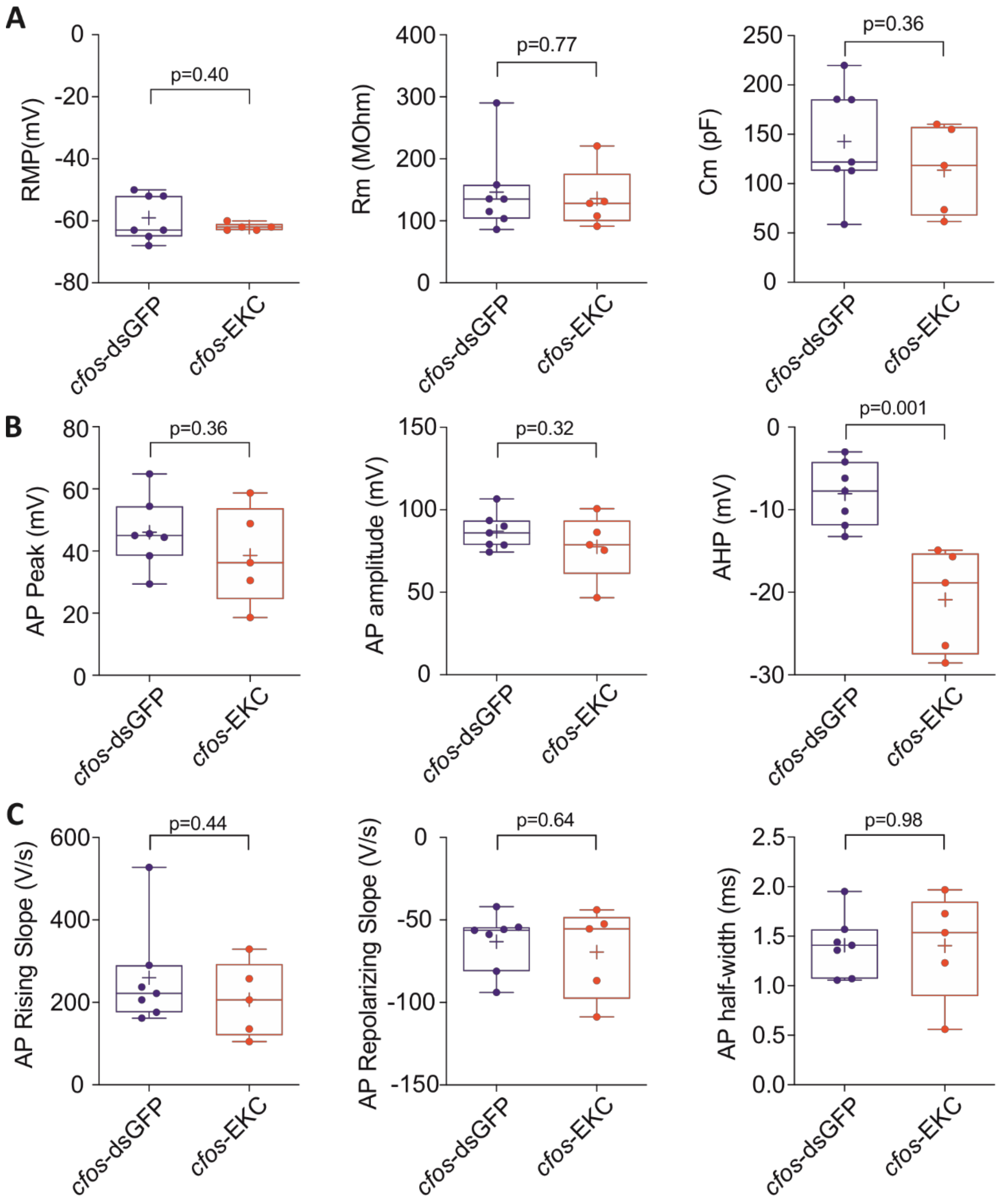

Fig. S9. Passive and active neuronal properties following *cfos*-EKC gene therapy after a single chemoconvulsant challenge. A. Passive neuronal properties in neurons transduced with either *cfos*-dsGFP or *cfos*-EKC: resting membrane potential (RMP), membrane resistance (Rm) and capacitance (Cm). **B.** Single action potential (AP) parameters: peak, amplitude and after hyperpolarization (AHP). **C.** AP shape: rising slope, repolarizing slope and AP half-width. Student's t tests.

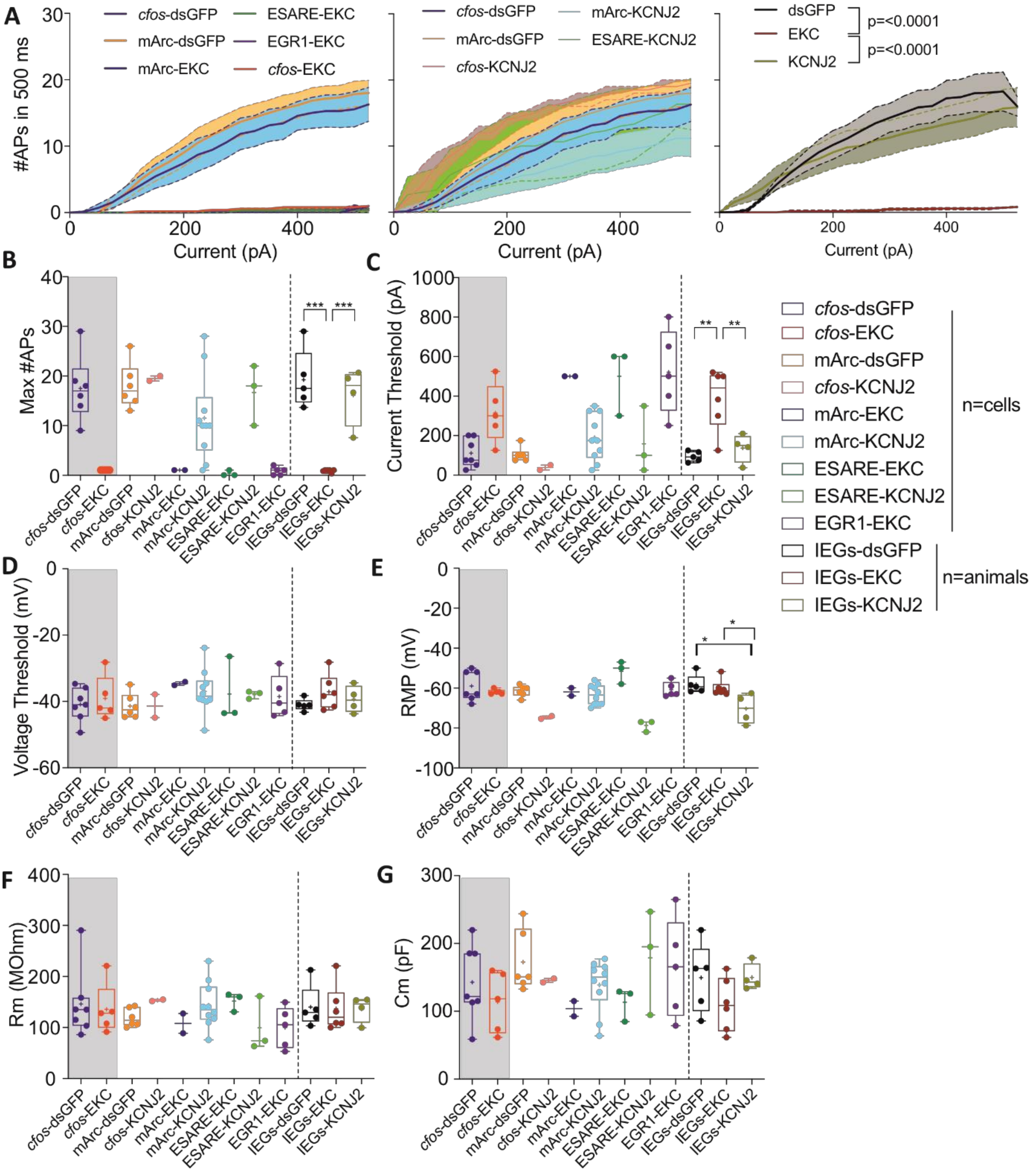

Fig. S10. Activity-dependent gene therapies decreased neuronal excitability. Graphs showing *ex vivo* neuronal excitability parameters of neurons expressing [*IEG*]-dsGFP, [*IEG*]-EKC or [*IEG*]-*KCNJ2* after a single chemoconvulsant-evoked generalized seizure. **A.** Number of action potentials evoked by increasing current steps for different *IEG*/Transgene combinations (n=cells). Right: dsGFP, EKC and

KCNJ2 driven by different activity-dependent promoters were each averaged, showing that EKC profoundly attenuated spiking (n=animals). One-way ANOVA. **B-D.** Maximum number of evoked action potential, current and voltage threshold are plotted for different *IEG*/Transgene combinations (n=cells) and for the averaged dsGFP, EKC and *KCNJ2* combinations (n=animals). ***p<0.001; **p<0.01 One-way ANOVA followed by Bonferroni multiple comparison test. **E-G.** Passive properties: RMP, membrane resistance and capacitance for different *IEG*/Transgene combinations (n=cells) and for the averaged dsGFP, EKC and *KCNJ2* combinations (n=animals). *p<0.05 One-way ANOVA followed by Bonferroni multiple comparison test. Grey shading: data replotted from Fig. 3C.

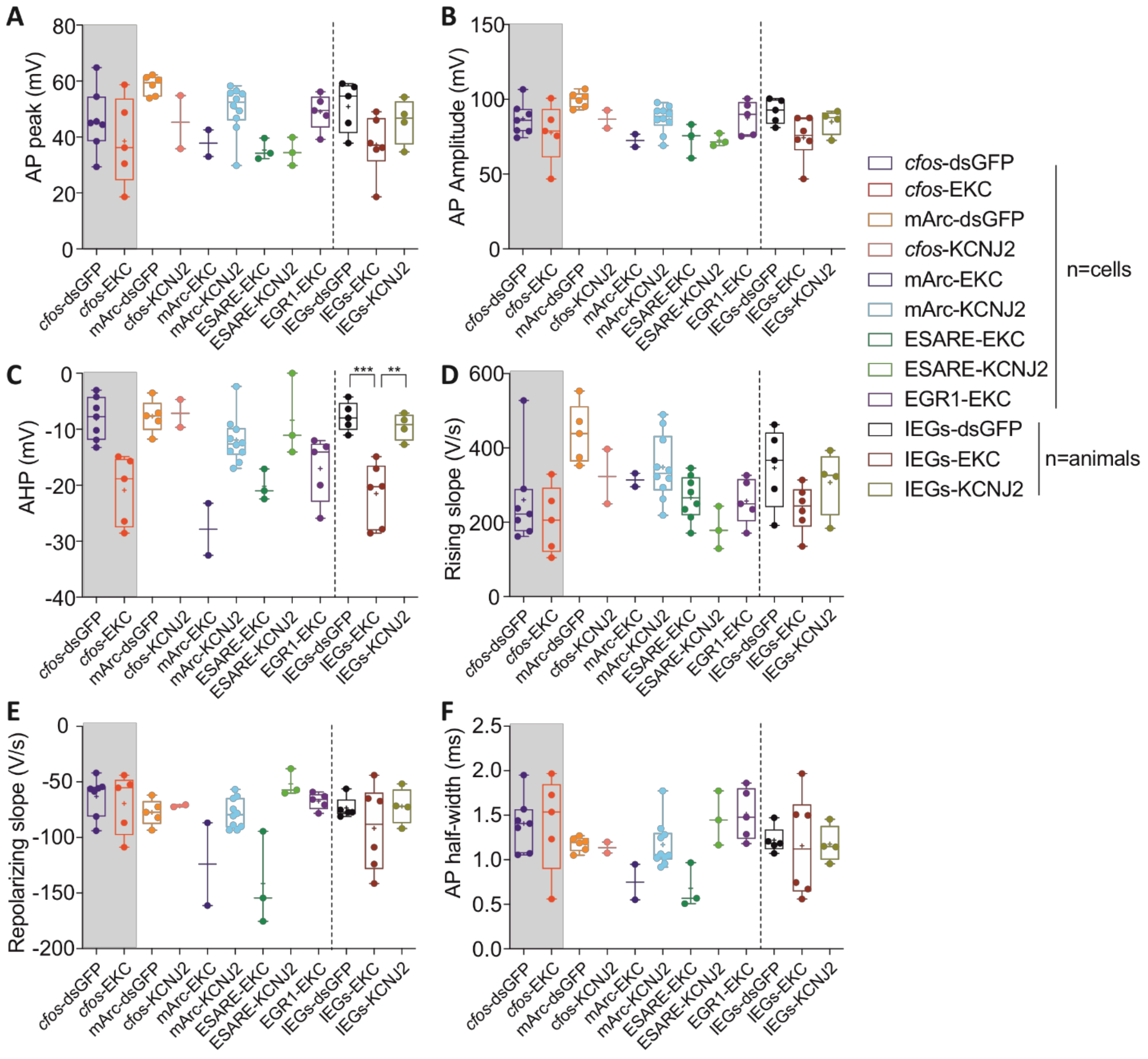

Fig. S11. Activity-dependent gene therapies modify action potential properties. Graphs showing *ex vivo* neuronal excitability parameters of neurons expressing [*IEG*]-dsGFP, [*IEG*]-EKC or [*IEG*]-*KCNJ2* after a single chemoconvulsant-evoked generalized seizure. **A-C.** Single action potential (AP) parameters: peak (A), amplitude (B) and after hyperpolarization (AHP) (C) for different *IEG*/Transgene combinations (n=cells) and for the averaged dsGFP, EKC and *KCNJ2* combinations (n=animals). **D-F.** AP shape: rising slope (D), repolarizing slope (E) and AP half-width (F) for different *IEG*/Transgene combinations (n=cells) and for the averaged dsGFP, EKC and *KCNJ2* combinations (n=animals). ***p<0.001; **p<0.01 One-way

ANOVA followed by Bonferroni multiple comparison test. Grey shading: data replotted from Fig. 3C.

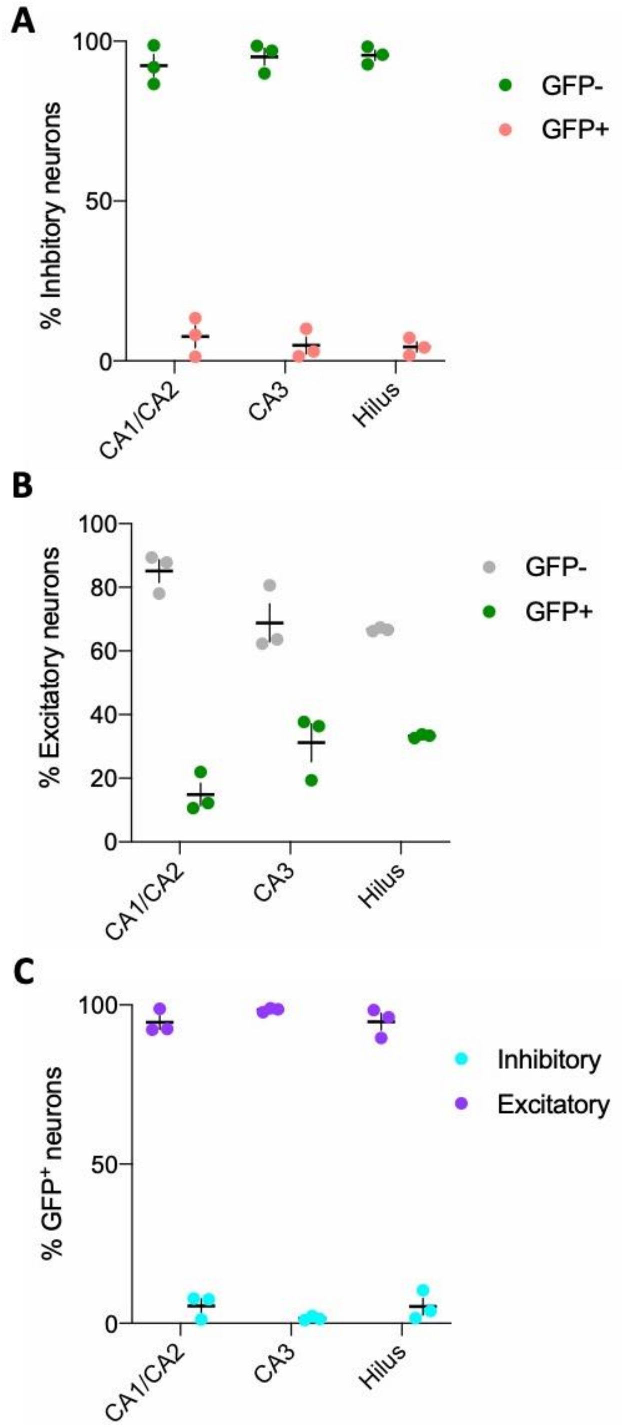

Fig. S12. Immunofluorescence analysis. All points graph of the percentage of inhibitory neurons positive or negative for dsGFP (**A**), of excitatory neurons positive or negative for dsGFP (**B**), and of dsGFP positive neurons identified as excitatory or inhibitory (**C**) (n=3 animals). Statistics can be found in Figure 3D legends.

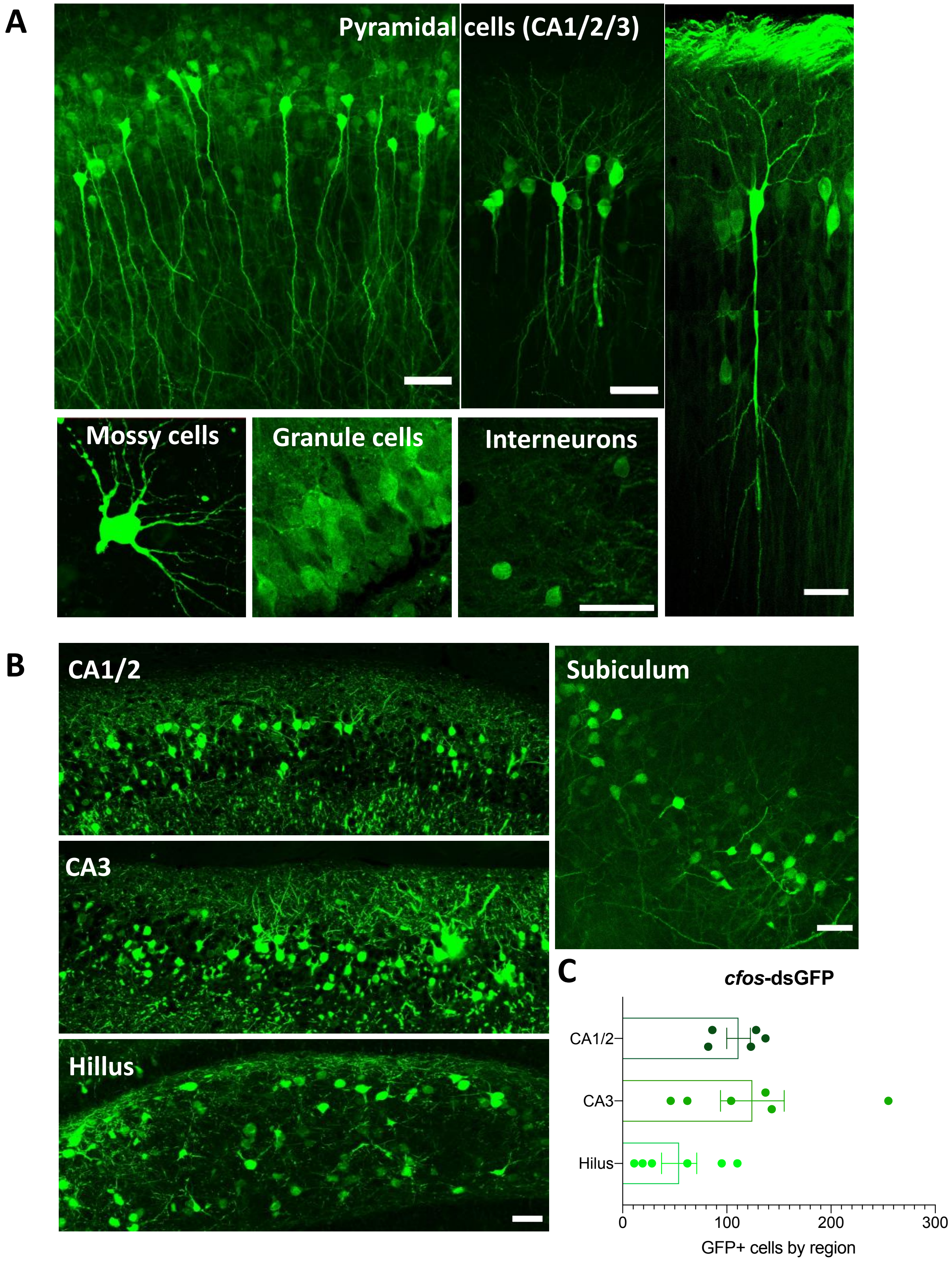

Fig. S13. Validating neuronal expression of *IEG*-GFP. A, B. For all immunofluorescence studies activation of AAV9 [*IEG*]-dsGFP was induced by PTZ injection. All brains were harvested and visualized for native dsGFP fluorescence.

Both (A) cellular and (B) regional targets were identified. **C.** Quantification of total dsGFP positive cells in CA1, CA3 and hilus regions of hippocampus for *cfos*-dsGFP (N=3, n=6) where N: number of mice and n: number of slices. Scale bars: 50 μm.

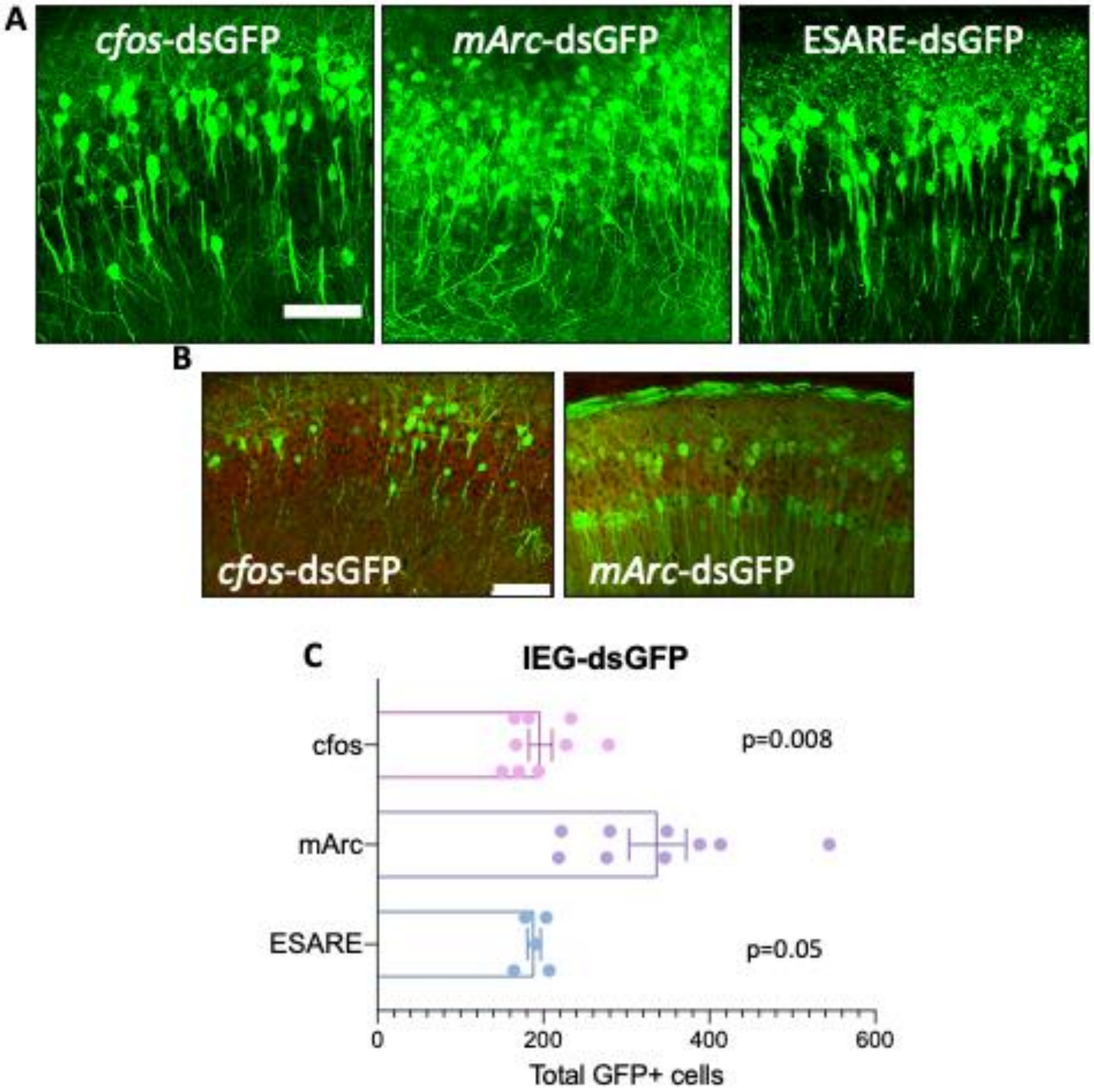

Fig. S14. AAV9 *mArc*-dsGFP was abundantly expressed in pyramidal cell layers. *IEG*-dsGFP expression was induced as previously described in Supplementary Figure 8 and total dsGFP positive cells were quantified in ventral hippocampus. **A.** Representative density micrographs of dsGFP positive CA1/2 pyramidal cells of mice injected with *cfos*-dsGFP, *mArc*-dsGFP or ESARE-dsGFP. **B.** Representative tile scans illustrating expression of mArc-dsGFP in deeper pyramidal layers. **C.** Total numbers of dsGFP positive cells were quantified for *cfos*-dsGFP (N=3, n=9), *mArc*-dsGFP (N=3 n=9) and ESARE-dsGFP (N=2, n=6). Scale

bar =100 μm. One-way ANOVA followed by Bonferroni multiple comparison test. P values represent comparison vs *mArc*-dsGFP.

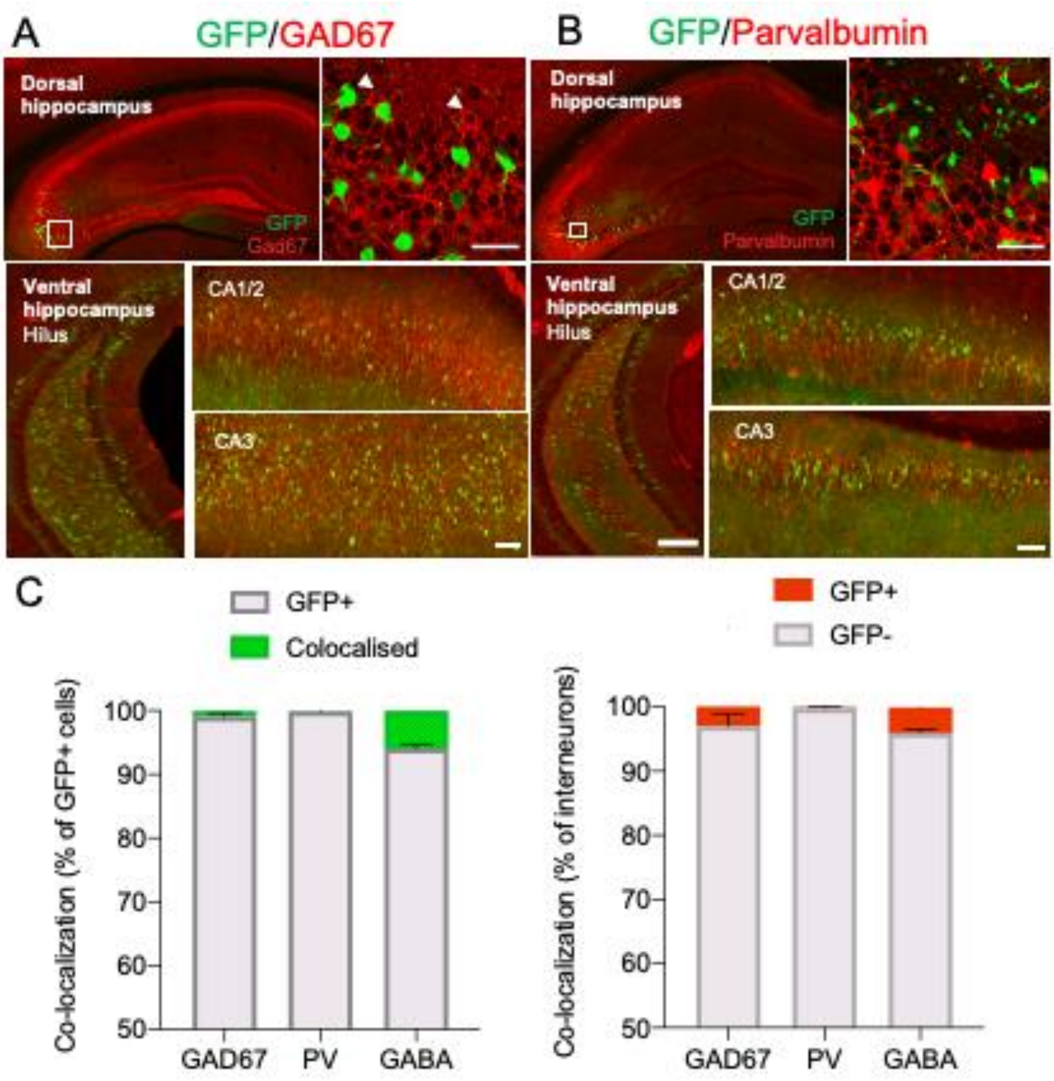

Fig. S15. *Cfos*-dsGFP mostly targets excitatory neurons. *Cfos*-dsGFP expression was induced as previously described in Suppplemntary Figure 8 and hippocampal slices were immunolabeled with either **(A)** GAD67 **(B)** parvalbumin (PV) or GABA (shown in Fig.3). **C.** Quantification of co-localization as a % of total dsGFP positive cells (*left*) and % of total interneuron marker-positive cells (*right*). Scale bars = 100 µm. N=3 n=6 where N= number of mice and n=number of slices quantified.

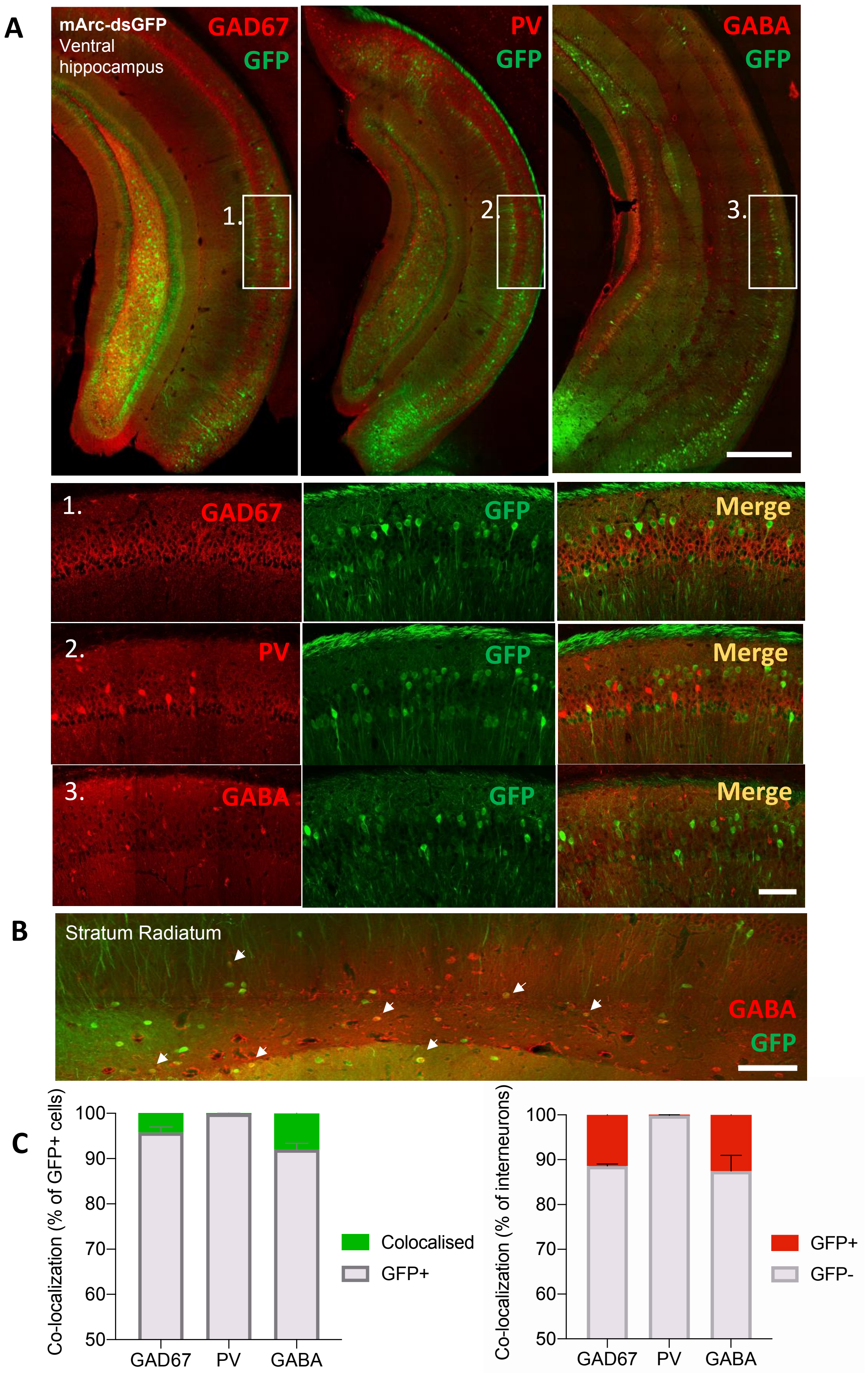
A
mArc-dsGFP
Ventral
hippocampus
GAD67
GFP
PV
GFP
GABA
GFP
1.
2.
3.
1.
GAD67
GFP
Merge
2.
PV
GFP
Merge
3.
GABA
GFP
Merge
B
Stratum Radiatum
GABA
GFP
C
Co-localization (% of GFP+ cells)
100
90
80
70
60
50
GAD67
PV
GABA
Colocalised
GFP+
Co-localization (% of interneurons)
100
90
80
70
60
50
GAD67
PV
GABA
GFP+
GFP-

Fig. S16. mArc-dsGFP expression in excitatory and inhibitory neurons. mArc-dsGFP expression was induced as previously described. **A.** Hippocampal sections were immunolabeled with either GAD67, parvalbumin or GABA (red) and colocalization with dsGFP was quantified. Scale bar = 500 µm **B.** Region of *stratum radiatum* where highest density of co-localization occurred. **C.** Quantification of co-localization as a % of total GFP+ cells (*left*) and % of total interneuron marker+ cells (*right*). Dorsal hippocampus. Scale bars = 100 µm. N=3 n=6 where N= number of mice and n=number of slices quantified.

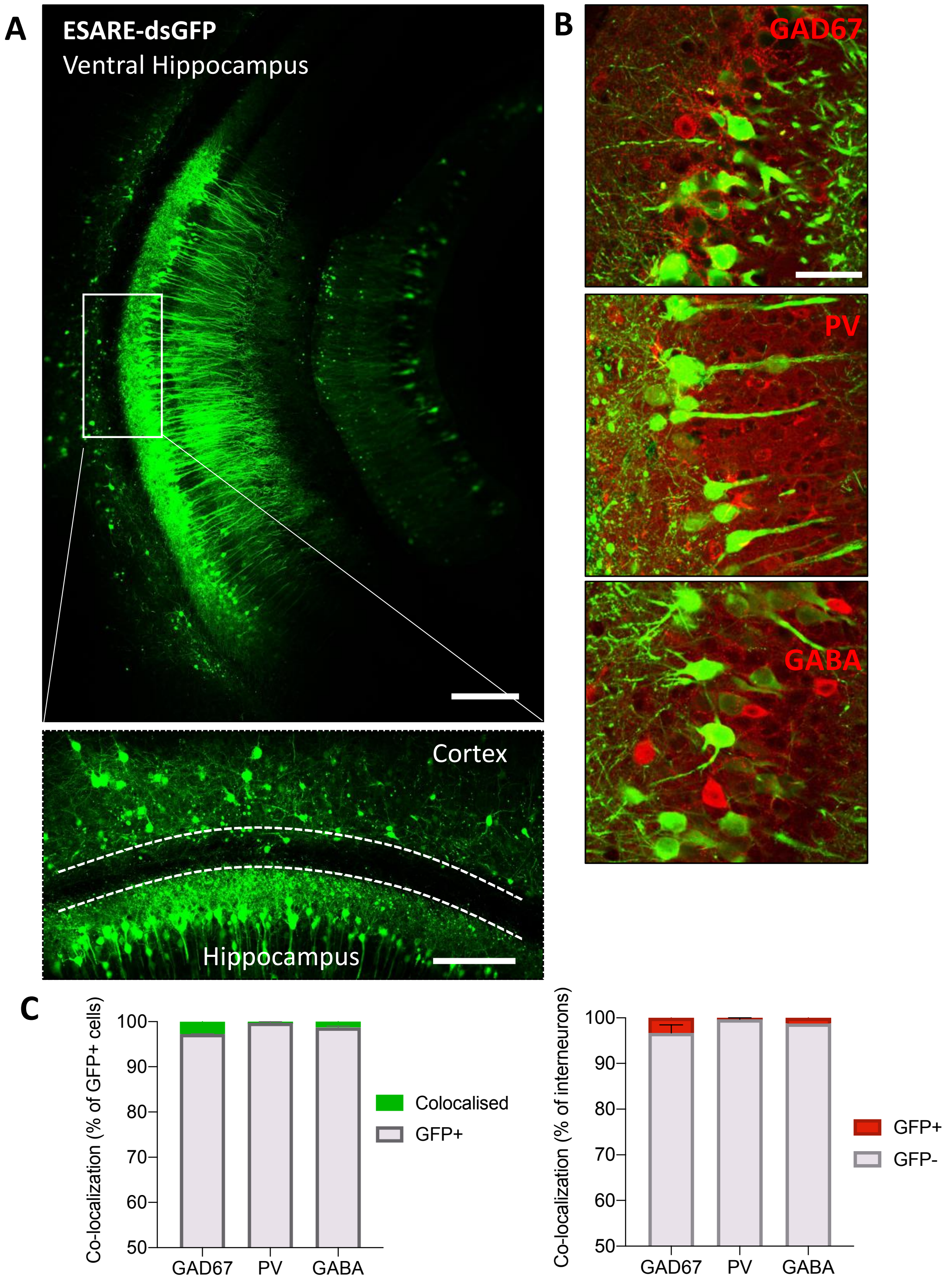

Fig. S17. ESARE-dsGFP expression in excitatory and inhibitory neurons.

ESARE-dsGFP expression was induced as previously described. **A.** Representative tile scan of ESARE-dsGFP expression in dorsal hippocampus. **B.** Representative

interneuron stains for GAD67, parvalbumin (PV) and GABA (scale bar = 50 μm). **C.** Quantification of co-localization as a % of total GFP+ cells (*top panel*) and % of total interneuron marker-positive cells (*bottom panel*). Scale bar = 200 μm.

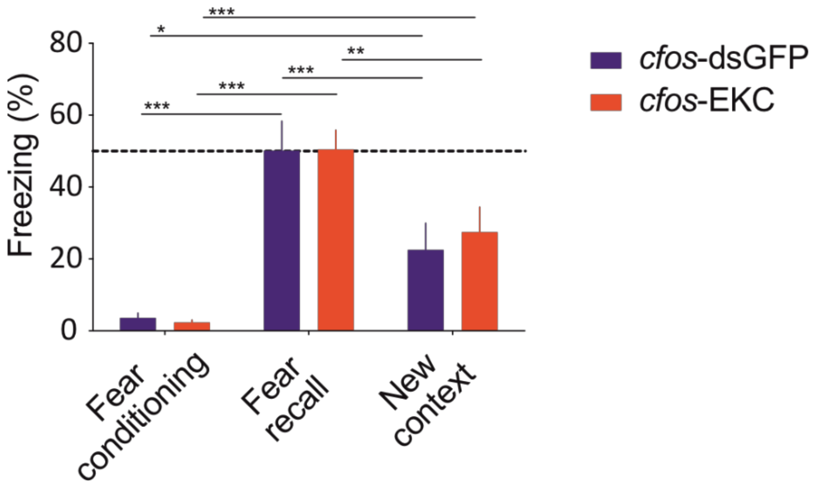

Fig. S18. Activity-dependent gene therapy does not affect contextual fear conditioning in naïve animals. Percentage of freezing time for naïve animals injected with either *cfos*-dsGFP (n=7) or *cfos*-EKC (n=7) during fear conditioning, fear recall and in a new context. **p<0.01; ***p<0.001; two-way ANOVA followed by Bonferroni multiple-comparison test.

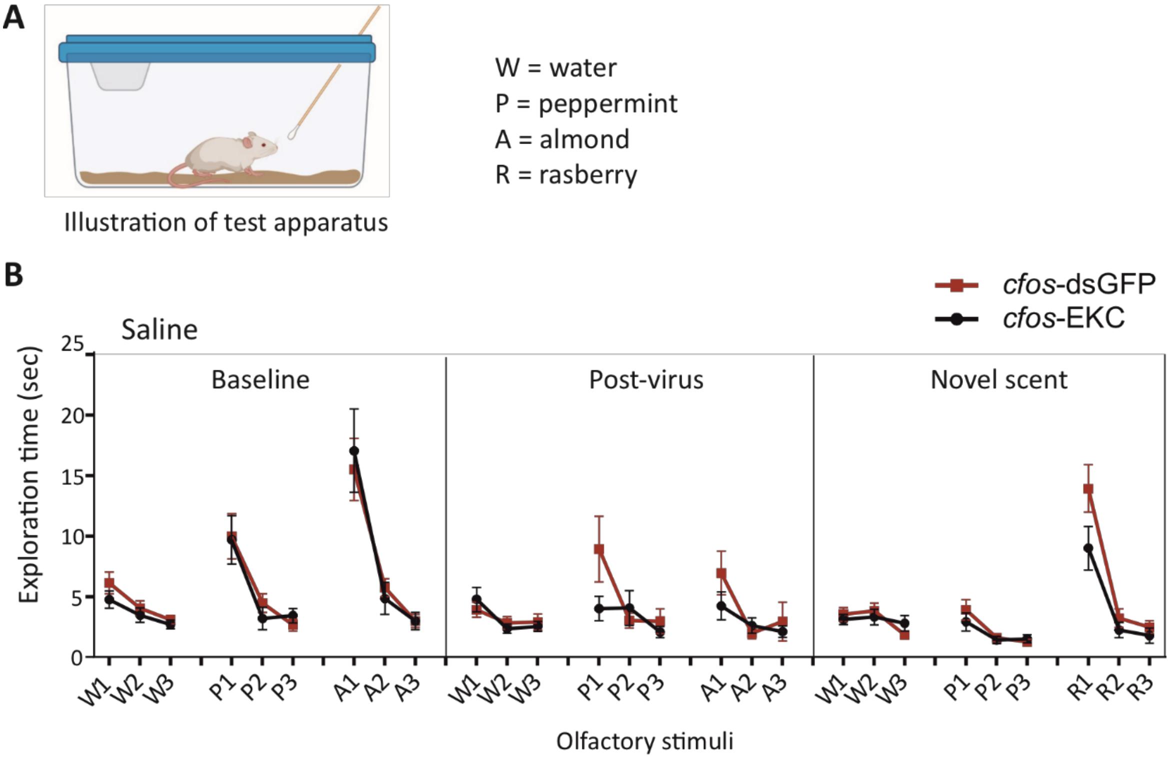

Fig. S19. Olfactory Discrimination test. A. Illustration of behavior chamber. **B.** Exploration time with different odorants in animal before and after treatment with either *cfos*-dsGFP or *cfos*-EKC. A novel scent (Raspberry) was used 24hrs after the test of post-virus animals. 3-way ANOVA followed by Bonferroni multiple comparison test.

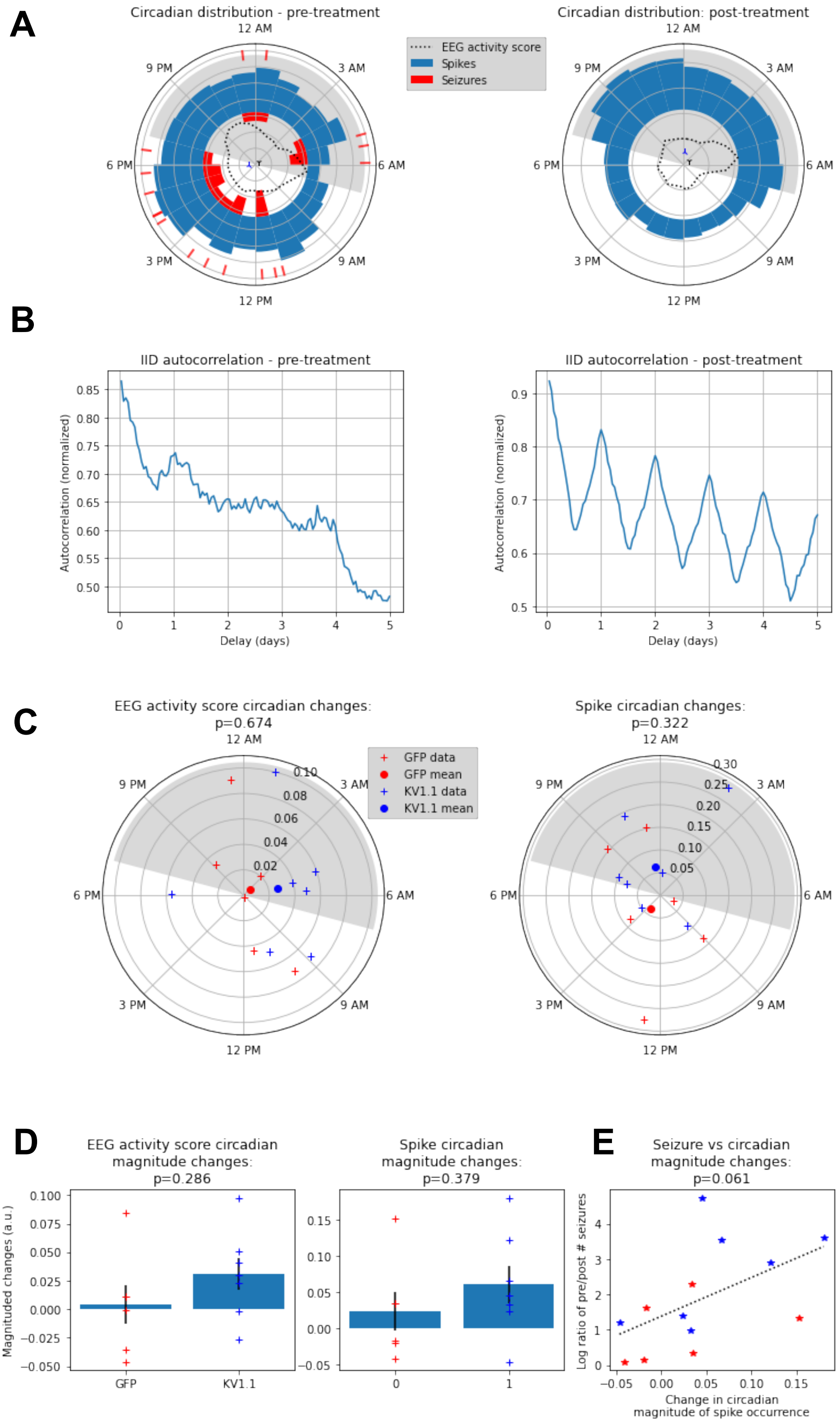

A
Circadian distribution - pre-treatment
Circadian distribution: post-treatment
12 AM
3 AM
6 AM
9 AM
12 PM
3 PM
6 PM
9 PM
EEG activity score
Spikes
Seizures
B
IID autocorrelation - pre-treatment
IID autocorrelation - post-treatment
Autocorrelation (normalized)
Delay (days)
C
EEG activity score circadian changes:
p=0.674
Spike circadian changes:
p=0.322
GFP data
GFP mean
KV1.1 data
KV1.1 mean
D
EEG activity score circadian magnitude changes:
p=0.286
Magnitude changes (a.u.)
GFP
KV1.1
Spike circadian magnitude changes:
p=0.379
E
Seizure vs circadian magnitude changes:
p=0.061
Log ratio of pre/post # seizures
Change in circadian magnitude of spike occurrence

Fig. S20. Changes in the magnitude of the circular means for EEG activity score and inter-ictal spike distributions after activity-dependent gene therapy. **A.** Circadian distribution of inter-ictal spikes (■), seizures (■) and EEG activity score (EAS) (···) pre (left) and post (right) treatment for an example *cfos*-EKC treated animal. The EAS is the ratio of the EEG power in the [12Hz-124Hz] frequency band over the power in the [1Hz-8Hz] frequency band. The circular means of the distribution of the spike and EAS are annotated with (**+**,**+** respectively). The light and dark periods of the day are represented with white and gray backgrounds. **B.** Normalized auto-correlation of the hourly spike frequency over the pre (*left*) and post-treatment (*right*) periods for the same *cfos*-EKC treated animal as in panel A. **C.** Changes in the circular means of the EAS (*left*) and inter-ictal spike (*right*) distributions (post minus pre-treatment). The bi-variate means are compared using an F-test. **D.** Changes in the magnitude of the circular means for the EAS (left) and inter-ictal spike (right) distributions (post minus pre-treatment). The means are compared using two-sample t-test. **E.** Changes in the magnitude of the circular mean of the spike distributions (post minus pre-treatment) plotted versus the log ratio of the number of seizures (+1) pre and post treatment.

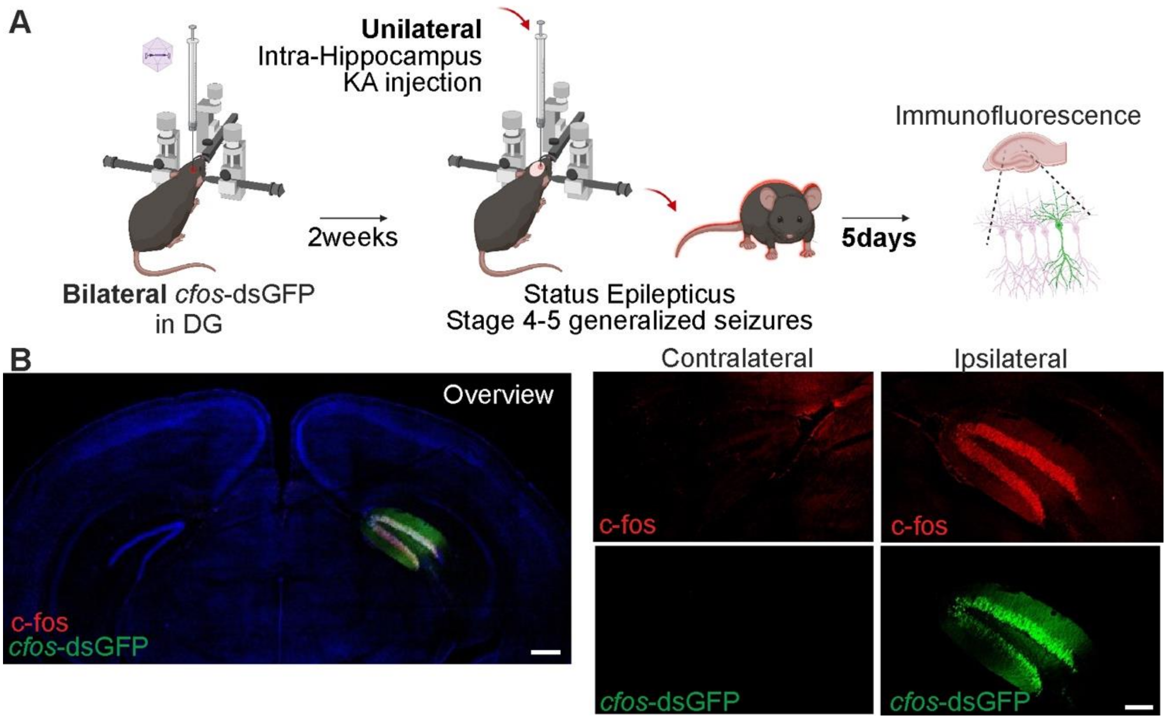

Fig. S21. *cfos*-driven transgene expression was activated by interictal activity. **A.** Experimental timeline. AAV9 *cfos*-dsGFP was injected bilaterally in the DG and SE was induced unilaterally by focal KA injection in dentate gyrus (DG). **B** Representative images showing dsGFP expression post-seizure for *cfos*-dsGFP. dsGFP driven by *cfos* showed restricted expression in the ipsilateral DG. In our lab this model shows the first seizure at 13.25±2.2 (mean ± SD), with interictal spikes present in the hippocampus after SE (recorded with depth hippocampal electrodes). Scale bar overview = 500μm; Scale bar zoomed in images: 250 μm.

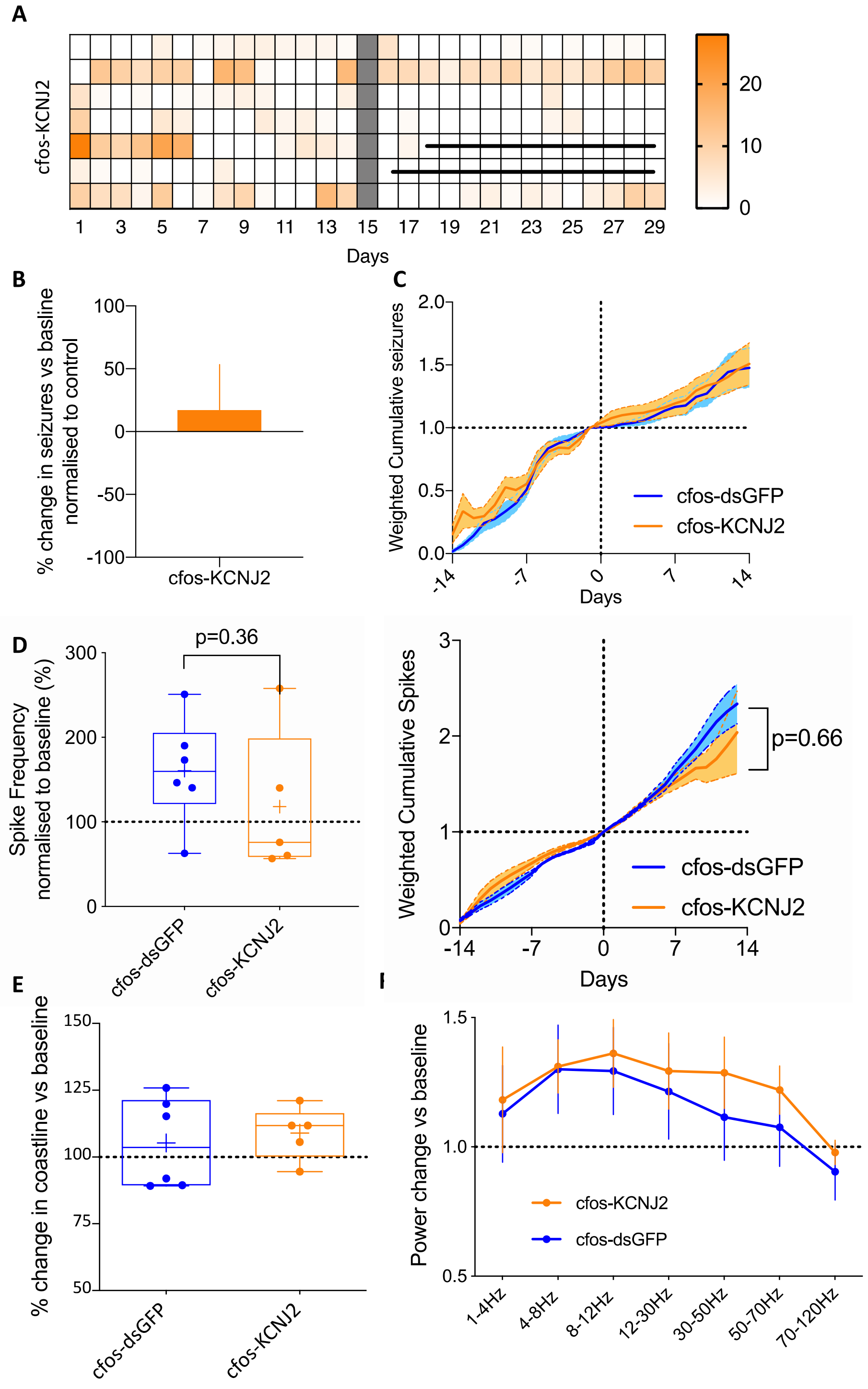

Fig. S22. *cfos*-KCNJ2 did not reduce spontaneous generalized seizures and interictal spikes. A. Heat-map showing seizures over time (square= 1 day) for each mouse (lines). Gray boxes indicate the viral injection, followed by a 2-week period to allow viral expression. **B.** Percentage change in spontaneous generalized seizures

after *cfos-KCNJ2* treatment compared to baseline, normalized to the same percentage of change in *cfos*-dsGFP treated mice (re-plotted from Fig. 5D). **C.** Weighted cumulative plot normalized by the total seizure count before treatment. **D.** *Left:* Spike frequency normalized to baseline (before viral injection). Student's t test. *Right:* Weighted cumulative plot normalized by the total interictal spike count before viral treatment (*cfos*-dsGFP re-plotted from Fig. 5D). Two-way ANOVA. **E.** Percentage change in coastline normalized to baseline (before viral injection) (*cfos*-dsGFP re-plotted from Fig. 5F). Student's t test. **F.** Percentage power change vs baseline in different frequency bands (*cfos*-dsGFP re-plotted from Fig. 5D). Two-way ANOVA.

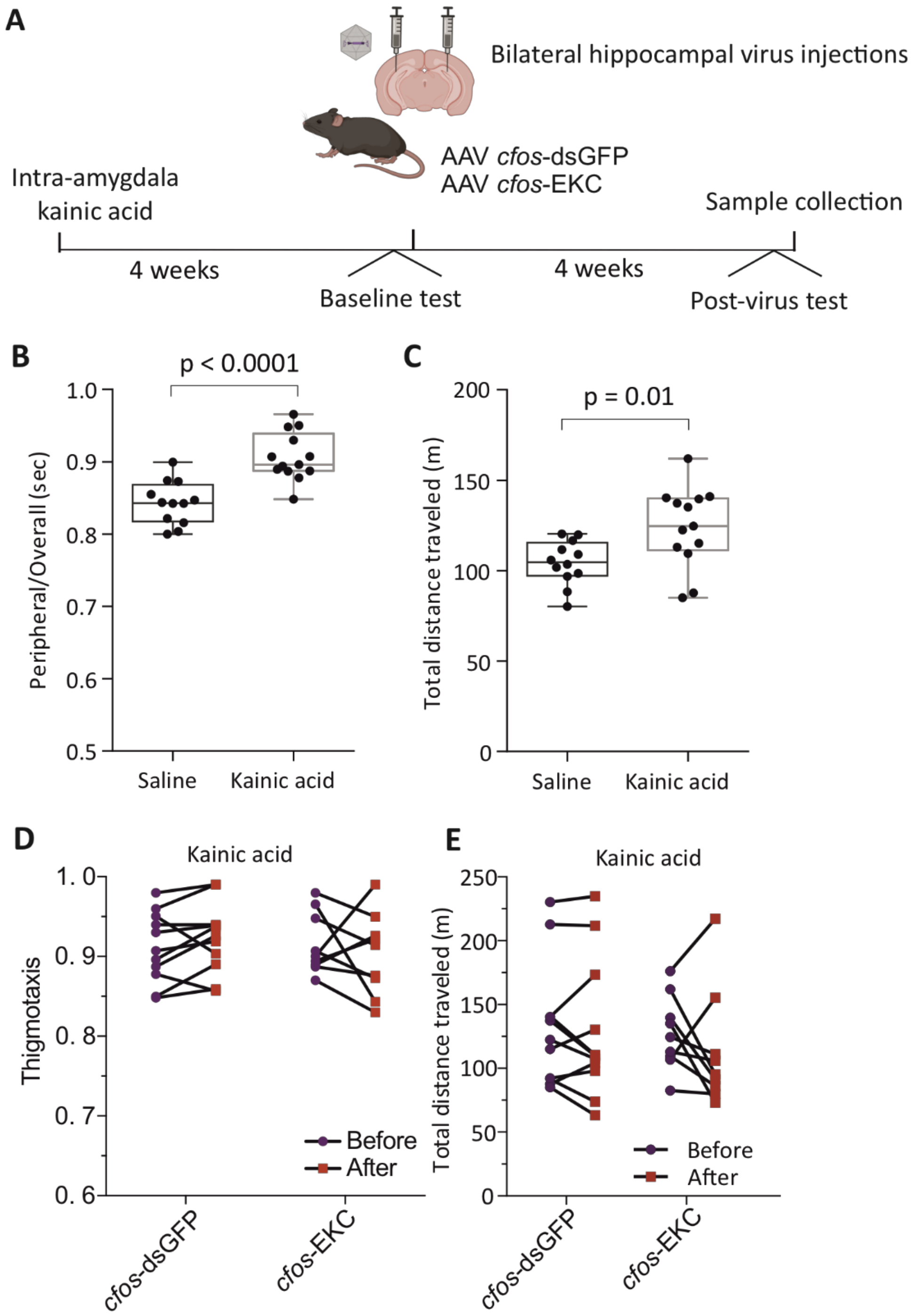

Fig. S23. Activity-dependent gene therapy does not worsen open field behavior in epileptic animals. A. Schematic of experimental plan for all behaviour tests. **B, C.** Open field test. Thigmotaxis (ratio of the time spent in the periphery vs total time, B) and distance traveled (C), were analyzed in naïve and epileptic animals. Student's

t test. **D, E.** Thigmotaxis (D) and distance traveled (E), were analyzed before and after either *cfos*-dsGFP or *cfos*-EKC injection in both hippocampi in epileptic animals.

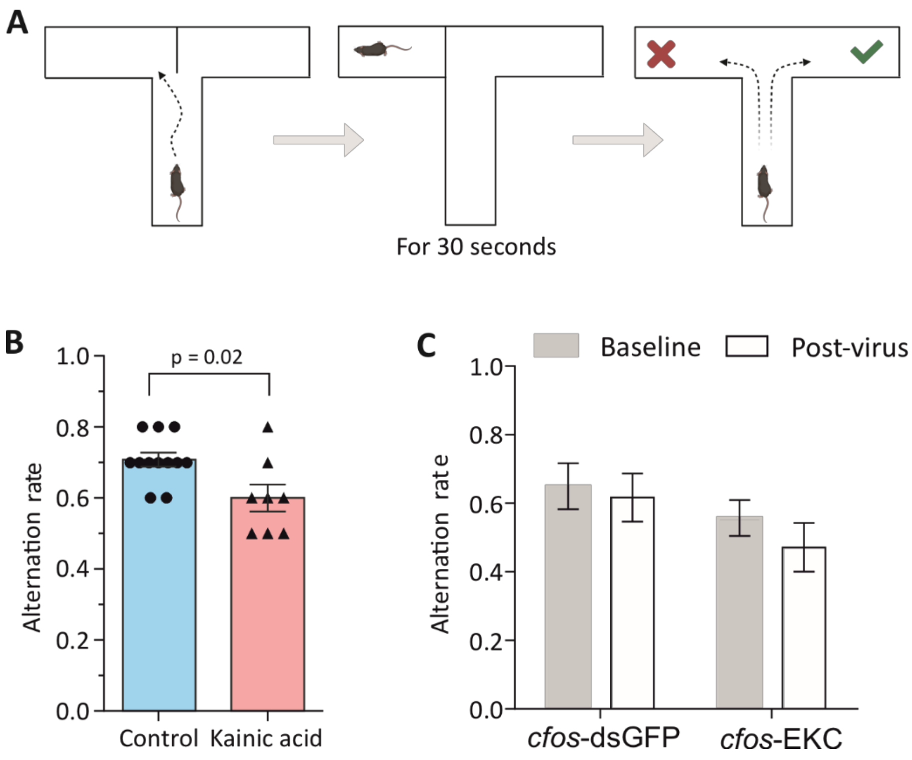

Fig. S24. Activity-dependent gene therapy does not worsen performance in the T-Maze alternation test in epileptic animals. Schematic of experimental plan for all behavior tests in Suppl. Figure 16. **A.** Schematic of the T-Maze spontaneous alteration test. **B.** Alternation rate in naïve and epileptic animals. Student's t test. **C.** Alternation rate before and after either *cfos*-dsGFP or *cfos*-EKC injection in both hippocampi in epileptic animals.

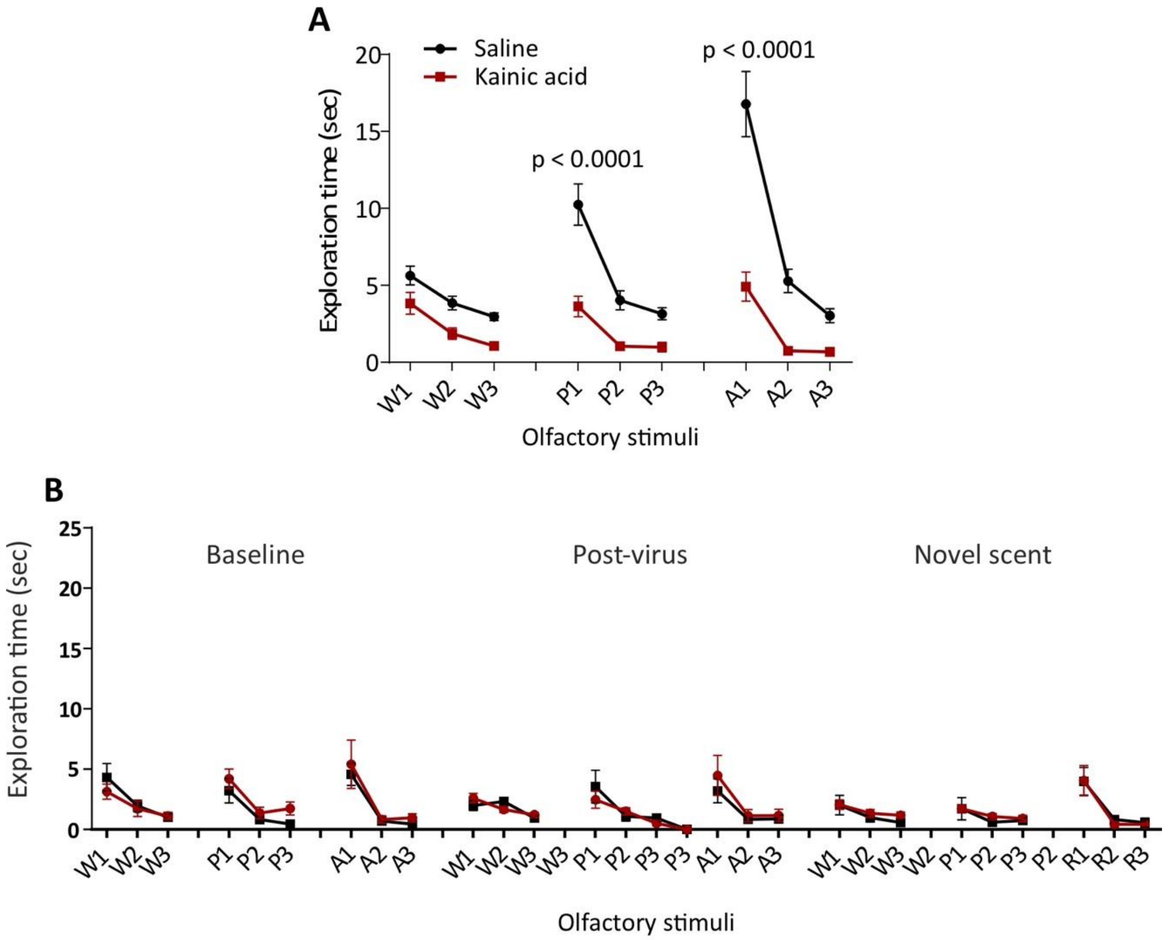

Fig. S25. Activity-dependent gene therapy does not worsen olfactory discrimination in epileptic animals. Schematic of experimental plan for all behavior tests in Suppl. Figure 16. **A.** Exploration time for different odorants was analyzed in naïve and epileptic animals. 3-Way ANOVA followed by Bonferroni multiple-comparison test. **B.** Exploration time for different odor stimuli was analyzed before and after either *cfos*-dsGFP (black) or *cfos*-EKC (red) injection in both hippocampi in epileptic animals.

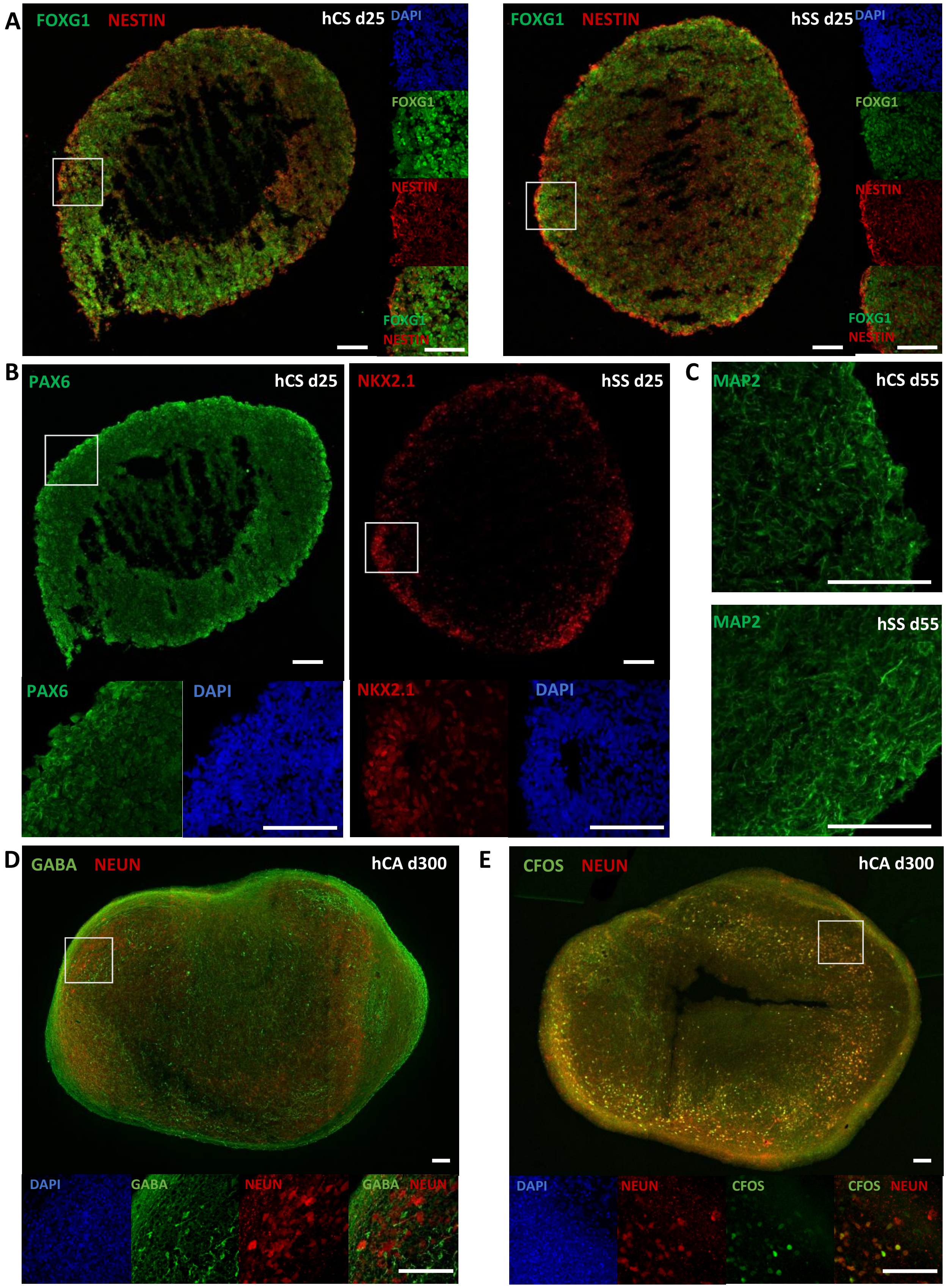
A
FOXG1 NESTIN
hCS d25
DAPI
FOXG1
NESTIN
FOXG1
NESTIN
FOXG1 NESTIN
hSS d25
DAPI
FOXG1
NESTIN
FOXG1
NESTIN
B
PAX6
hCS d25
NKX2.1
hSS d25
PAX6
DAPI
NKX2.1
DAPI
C
MAP2
hCS d55
MAP2
hSS d55
D
GABA NEUN
hCA d300
DAPI
GABA
NEUN
GABA NEUN
E
CFOS NEUN
hCA d300
DAPI
NEUN
CFOS
CFOS NEUN

Fig. S26. Characterization of cortical assembloids. A. Immunostaining of hCSs and hSSs at day 25 for Nestin and FOXG1 markers of neural stem cells and forebrain identities, respectively. **B.** Immunostaining for the dorsal forebrain marker PAX6 and the ventral forebrain marker NKX2.1 in hCSs and hSSs respectively at day 25. **C.** Immunostaining for MAP2 in hCSs and hSSs at day 55. **D.** Immunostaining for GABA and NeuN at day 240 showing the presence of mature GABAergic neurons. **E.** Immunostaining at day 240 indicating mature neurons expressing c-Fos. All scale bars: 100μm.

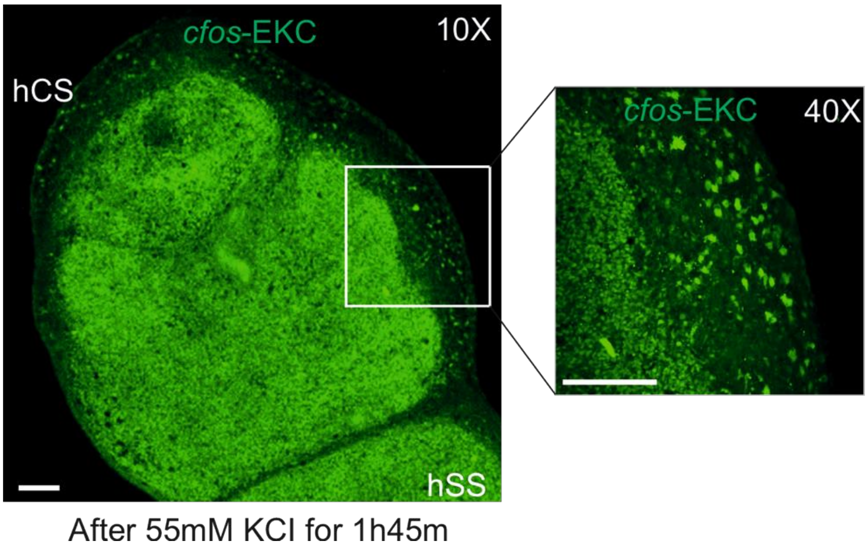

Fig. S27. *Cfos*-EKC efficacy in cortical assembloids. Image of an assembloid transduce with *cfos*-EKC after 1 hour and 45 min in KCl. Scale bars: 100μm.

References and Notes

1. P. Fusar-Poli, S. Borgwardt, A. Bechdolf, J. Addington, A. Riecher-Rössler, F. Schultze-Lutter, M. Keshavan, S. Wood, S. Ruhrmann, L. J. Seidman, L. Valmaggia, T. Cannon, E. Velthorst, L. De Haan, B. Cornblatt, I. Bonoldi, M. Birchwood, T. McGlashan, W. Carpenter, P. McGorry, J. Klosterkötter, P. McGuire, A. Yung, The psychosis high-risk state: A comprehensive state-of-the-art review. *JAMA Psychiatry* **70**, 107–120 (2013).

2. S. Harvey, M. D. King, K. M. Gorman, Paroxysmal movement disorders. *Front. Neurol.* **12**, 659064 (2021).

3. K. Staley, Molecular mechanisms of epilepsy. *Nat. Neurosci.* **18**, 367–372 (2015).

4. M. J. Brodie, Sodium channel blockers in the treatment of epilepsy. *CNS Drugs* **31**, 527–534 (2017).

5. D. M. Kullmann, S. Schorge, M. C. Walker, R. C. Wykes, Gene therapy in epilepsy-is it time for clinical trials? *Nat. Rev. Neurol.* **10**, 300–304 (2014).

6. T. J. Turner, C. Zourray, S. Schorge, G. Lignani, Recent advances in gene therapy for neurodevelopmental disorders with epilepsy. *J. Neurochem.* **157**, 229–262 (2021).

7. K. M. Tye, Neural circuit reprogramming: A new paradigm for treating neuropsychiatric disease? *Neuron* **83**, 1259–1261 (2014).

8. R. C. Wykes, G. Lignani, Gene therapy and editing: Novel potential treatments for neuronal channelopathies. *Neuropharmacology* **132**, 108–117 (2018).

9. B. E. Deverman, B. M. Ravina, K. S. Bankiewicz, S. M. Paul, D. W. Y. Sah, Gene therapy for neurological disorders: Progress and prospects. *Nat. Rev. Drug Discov.* **17**, 641–659 (2018).

10. A. Lieb, Y. Qiu, C. L. Dixon, J. P. Heller, M. C. Walker, S. Schorge, D. M. Kullmann, Biochemical autoregulatory gene therapy for focal epilepsy. *Nat. Med.* **24**, 1324–1329 (2018).

11. R. C. Wykes, J. H. Heeroma, L. Mantoan, K. Zheng, D. C. MacDonald, K. Deisseroth, K. S. Hashemi, M. C. Walker, S. Schorge, D. M. Kullmann, Optogenetic and potassium channel gene therapy in a rodent model of focal neocortical epilepsy. *Sci. Transl. Med.* **4**, 161ra152 (2012).

12. D. Kätzel, E. Nicholson, S. Schorge, M. C. Walker, D. M. Kullmann, Chemical-genetic attenuation of focal neocortical seizures. *Nat. Commun.* **5**, 3847 (2014).

13. F. M. Noe, A. T. Sorensen, M. Kokaia, A. Vezzani, in *Jasper's Basic Mechanisms of the Epilepsies*, J. L. Noebels, M. Avoli, M. A. Rogawski, R. W. Olsen, A. V. Delgado-Escueta, Eds. (Oxford Univ. Press, 2012).

14. G. Colasante, Y. Qiu, L. Massimino, C. Di Berardino, J. H. Cornford, A. Snowball, M. Weston, S. P. Jones, S. Giannelli, A. Lieb, S. Schorge, D. M. Kullmann, V. Broccoli, G. Lignani, In vivo CRISPRa decreases seizures and rescues cognitive deficits in a rodent model of epilepsy. *Brain* **143**, 891–905 (2020).

15. A. Snowball, E. Chabrol, R. C. Wykes, T. Shekh-Ahmad, J. H. Cornford, A. Lieb, M. P. Hughes, G. Massaro, A. A. Rahim, K. S. Hashemi, D. M. Kullmann, M. C. Walker, S.

Schorge, Epilepsy gene therapy using an engineered potassium channel. *J. Neurosci.* **39**, 3159–3169 (2019).

16. A. Nanobashvili, E. Melin, D. Emerich, J. Tornøe, M. Simonato, L. Wahlberg, M. Kokaia, Unilateral ex vivo gene therapy by GDNF in epileptic rats. *Gene Ther.* **26**, 65–74 (2019).
17. P. Kwan, S. C. Schachter, M. J. Brodie, Drug-resistant epilepsy. *N. Engl. J. Med.* **365**, 919–926 (2011).
18. F. Tang, A. M. S. Hartz, B. Bauer, Drug-resistant epilepsy: Multiple hypotheses, few answers. *Front. Neurol.* **8**, 301 (2017).
19. W. Truccolo, J. A. Donoghue, L. R. Hochberg, E. N. Eskandar, J. R. Madsen, W. S. Anderson, E. N. Brown, E. Halgren, S. S. Cash, Single-neuron dynamics in human focal epilepsy. *Nat. Neurosci.* **14**, 635–641 (2011).
20. M. Wenzel, J. P. Hamm, D. S. Peterka, R. Yuste, Reliable and elastic propagation of cortical seizures in vivo. *Cell Rep.* **19**, 2681–2693 (2017).
21. S. W. Flavell, M. E. Greenberg, Signaling mechanisms linking neuronal activity to gene expression and plasticity of the nervous system. *Annu. Rev. Neurosci.* **31**, 563–590 (2008).
22. E. L. Yap, M. E. Greenberg, Activity-regulated transcription: Bridging the gap between neural activity and behavior. *Neuron* **100**, 330–348 (2018).
23. T. Kawashima, H. Okuno, H. Bito, A new era for functional labeling of neurons: Activity-dependent promoters have come of age. *Front. Neural Circuits* **8**, 37 (2014).
24. N. Dabrowska, S. Joshi, J. Williamson, E. Lewczuk, Y. Lu, S. Oberoi, A. Brodovskaya, J. Kapur, Parallel pathways of seizure generalization. *Brain* **142**, 2336–2351 (2019).
25. S. R. Haut, R. B. Lipton, A. J. LeValley, C. B. Hall, S. Shinnar, Identifying seizure clusters in patients with epilepsy. *Neurology* **65**, 1313–1315 (2005).
26. J. H. Heeroma, C. Henneberger, S. Rajakulendran, M. G. Hanna, S. Schorge, D. M. Kullmann, Episodic ataxia type 1 mutations differentially affect neuronal excitability and transmitter release. *Dis. Model. Mech.* **2**, 612–619 (2009).
27. A. L. Lee, T. C. Dumas, P. E. Tarapore, B. R. Webster, D. Y. Ho, D. Kaufer, R. M. Sapolsky, Potassium channel gene therapy can prevent neuron death resulting from necrotic and apoptotic insults. *J. Neurochem.* **86**, 1079–1088 (2003).
28. H. J. Wenzel, H. Vacher, E. Clark, J. S. Trimmer, A. L. Lee, R. M. Sapolsky, B. L. Tempel, P. A. Schwartzkroin, Structural consequences of Kcna1 gene deletion and transfer in the mouse hippocampus. *Epilepsia* **48**, 2023–2046 (2007).
29. T. Kawashima, K. Kitamura, K. Suzuki, M. Nonaka, S. Kamijo, S. Takemoto-Kimura, M. Kano, H. Okuno, K. Ohki, H. Bito, Functional labeling of neurons and their projections using the synthetic activity-dependent promoter E-SARE. *Nat. Methods* **10**, 889–895 (2013).
30. A. T. Sørensen, Y. A. Cooper, M. V. Baratta, F. J. Weng, Y. Zhang, K. Ramamoorthi, R. Fropf, E. LaVerriere, J. Xue, A. Young, C. Schneider, C. R. Gøtzsche, M. Hemberg, J. C.

Yin, S. F. Maier, Y. Lin, A robust activity marking system for exploring active neuronal ensembles. *eLife* **5**, e13918 (2016).

31. X. Sun, M. J. Bernstein, M. Meng, S. Rao, A. T. Sørensen, L. Yao, X. Zhang, P. O. Anikeeva, Y. Lin, Functionally distinct neuronal ensembles within the memory engram. *Cell* **181**, 410–423.e17 (2020).

32. N. A. Hager, C. K. McAtee, M. A. Lesko, A. F. O'Donnell, Inwardly rectifying potassium channel Kir2.1 and its "Kir-ious" regulation by protein trafficking and roles in development and disease. *Front. Cell Dev. Biol.* **9**, 796136 (2022).

33. C. R. Yu, J. Power, G. Barnea, S. O'Donnell, H. E. V. Brown, J. Osborne, R. Axel, J. A. Gogos, Spontaneous neural activity is required for the establishment and maintenance of the olfactory sensory map. *Neuron* **42**, 553–566 (2004).

34. G. Colasante, G. Lignani, S. Brusco, C. Di Berardino, J. Carpenter, S. Giannelli, N. Valassina, S. Bido, R. Ricci, V. Castoldi, S. Marenna, T. Church, L. Massimino, G. Morabito, F. Benfenati, S. Schorge, L. Leocani, D. M. Kullmann, V. Broccoli, dCas9-based Scn1a gene activation restores inhibitory interneuron excitability and attenuates seizures in Dravet syndrome mice. *Mol. Ther.* **28**, 235–253 (2020).

35. V. Magloire, J. Cornford, A. Lieb, D. M. Kullmann, I. Pavlov, KCC2 overexpression prevents the paradoxical seizure-promoting action of somatic inhibition. *Nat. Commun.* **10**, 1225 (2019).

36. J. Van Erum, D. Van Dam, P. P. De Deyn, PTZ-induced seizures in mice require a revised Racine scale. *Epilepsy Behav.* **95**, 51–55 (2019).

37. E. F. Fornasiero, S. Mandad, H. Wildhagen, M. Alevra, B. Rammner, S. Keihani, F. Opazo, I. Urban, T. Ischebeck, M. S. Sakib, M. K. Fard, K. Kirli, T. P. Centeno, R. O. Vidal, R.-U. Rahman, E. Benito, A. Fischer, S. Dennerlein, P. Rehling, I. Feussner, S. Bonn, M. Simons, H. Urlaub, S. O. Rizzoli, Precisely measured protein lifetimes in the mouse brain reveal differences across tissues and subcellular fractions. *Nat. Commun.* **9**, 4230 (2018).

38. F. T. Gallo, C. Katche, J. F. Morici, J. H. Medina, N. V. Weisstaub, Immediate early genes, memory and psychiatric disorders: Focus on c-Fos, Egr1 and Arc. *Front. Behav. Neurosci.* **12**, 79 (2018).

39. K. Minatohara, M. Akiyoshi, H. Okuno, Role of immediate-early genes in synaptic plasticity and neuronal ensembles underlying the memory trace. *Front. Mol. Neurosci.* **8**, 78 (2016).

40. K. Z. Tanaka, A. Pevzner, A. B. Hamidi, Y. Nakazawa, J. Graham, B. J. Wiltgen, Cortical representations are reinstated by the hippocampus during memory retrieval. *Neuron* **84**, 347–354 (2014).

41. A. R. Garner, D. C. Rowland, S. Y. Hwang, K. Baumgaertel, B. L. Roth, C. Kentros, M. Mayford, Generation of a synthetic memory trace. *Science* **335**, 1513–1516 (2012).

42. E. M. Jimenez-Mateos, T. Engel, P. Merino-Serrais, R. C. McKiernan, K. Tanaka, G. Mouri, T. Sano, C. O'Tuathaigh, J. L. Waddington, S. Prenter, N. Delanty, M. A. Farrell, D. F. O'Brien, R. M. Conroy, R. L. Stallings, J. DeFelipe, D. C. Henshall, Silencing

microRNA-134 produces neuroprotective and prolonged seizure-suppressive effects. *Nat. Med.* **18**, 1087–1094 (2012).

43. P. J. West, K. Thomson, P. Billingsley, T. Pruess, C. Rueda, G. W. Saunders, M. D. Smith, C. S. Metcalf, K. S. Wilcox, Spontaneous recurrent seizures in an intra-amygdala kainate microinjection model of temporal lobe epilepsy are differentially sensitive to antiseizure drugs. *Exp. Neurol.* **349**, 113954 (2022).

44. S. P. Paşca, Building three-dimensional human brain organoids. *Nat. Neurosci.* (2018).

45. S. A. Sloan, J. Andersen, A. M. Paşca, F. Birey, S. P. Paşca, Generation and assembly of human brain region-specific three-dimensional cultures. *Nat. Protoc.* **13**, 2062–2085 (2018).

46. C. Zourray, M. A. Kurian, S. Barral, G. Lignani, Electrophysiological properties of human cortical organoids: Current state of the art and future directions. *Front. Mol. Neurosci.* **15**, 839366 (2022).

47. F. Birey, J. Andersen, C. D. Makinson, S. Islam, W. Wei, N. Huber, H. C. Fan, K. R. C. Metzler, G. Panagiotakos, N. Thom, N. A. O'Rourke, L. M. Steinmetz, J. A. Bernstein, J. Hallmayer, J. R. Huguenard, S. P. Paşca, Assembly of functionally integrated human forebrain spheroids. *Nature* **545**, 54–59 (2017).

48. U. Vivekananda, P. Novak, O. D. Bello, Y. E. Korchev, S. S. Krishnakumar, K. E. Volynski, D. M. Kullmann, Kv1.1 channelopathy abolishes presynaptic spike width modulation by subthreshold somatic depolarization. *Proc. Natl. Acad. Sci. U.S.A.* **114**, 2395–2400 (2017).

49. L. A. Lau, K. J. Staley, K. P. Lillis, In vitro ictogenesis is stochastic at the single neuron level. *Brain* **145**, 531–541 (2022).

50. M. McHugo, P. Talati, K. Armstrong, S. N. Vandekar, J. U. Blackford, N. D. Woodward, S. Heckers, Hyperactivity and reduced activation of anterior hippocampus in early psychosis. *Am. J. Psychiatry* **176**, 1030–1038 (2019).

51. M. Jahanshahi, I. Obeso, C. Baunez, M. Alegre, P. Krack, Parkinson's disease, the subthalamic nucleus, inhibition, and impulsivity. *Mov. Disord.* **30**, 128–140 (2015).

52. P. J. Goadsby, P. R. Holland, M. Martins-Oliveira, J. Hoffmann, C. Schankin, S. Akerman, Pathophysiology of migraine: A disorder of sensory processing. *Physiol. Rev.* **97**, 553–622 (2017).

53. K. D. Fitzgerald, R. C. Welsh, W. J. Gehring, J. L. Abelson, J. A. Himle, I. Liberzon, S. F. Taylor, Error-related hyperactivity of the anterior cingulate cortex in obsessive-compulsive disorder. *Biol. Psychiatry* **57**, 287–294 (2005).

54. M. Przybyla, J. van Eersel, A. van Hummel, J. van der Hoven, M. Sabale, A. Harasta, J. Müller, M. Gajwani, E. Prikas, T. Mueller, C. H. Stevens, J. Power, G. D. Housley, T. Karl, M. Kassiou, Y. D. Ke, A. Ittner, L. M. Ittner, Onset of hippocampal network aberration and memory deficits in P301S tau mice are associated with an early gene signature. *Brain* **143**, 1889–1904 (2020).

55. S. Varma, J. Voldman, A cell-based sensor of fluid shear stress for microfluidics. *Lab Chip* **15**, 1563–1573 (2015).

56. J. C. Carpenter, R. Männikkö, C. Heffner, J. Heneine, M. Sampedro-Castañeda, G. Lignani, S. Schorge, Progressive myoclonus epilepsy *KCNC1* variant causes a developmental dendritopathy. *Epilepsia* **62**, 1256–1267 (2021).

57. J. C. Grieger, V. W. Choi, R. J. Samulski, Production and characterization of adeno-associated viral vectors. *Nat. Protoc.* **1**, 1412–1428 (2006).

58. D. Pozzi, G. Lignani, E. Ferrea, A. Contestabile, F. Paonessa, R. D'Alessandro, P. Lippiello, D. Boido, A. Fassio, J. Meldolesi, F. Valtorta, F. Benfenati, P. Baldelli, REST/NRSF-mediated intrinsic homeostasis protects neuronal networks from hyperexcitability. *EMBO J.* **32**, 2994–3007 (2013).

59. L. L. Bologna, V. Pasquale, M. Garofalo, M. Gandolfo, P. L. Baljon, A. Maccione, S. Martinoia, M. Chiappalone, Investigating neuronal activity by SPYCODE multi-channel data analyzer. *Neural Netw.* **23**, 685–697 (2010).

60. V. Pasquale, S. Martinoia, M. Chiappalone, A self-adapting approach for the detection of bursts and network bursts in neuronal cultures. *J. Comput. Neurosci.* **29**, 213–229 (2010).

61. J. Bischofberger, D. Engel, L. Li, J. R. Geiger, P. Jonas, Patch-clamp recording from mossy fiber terminals in hippocampal slices. *Nat. Protoc.* **1**, 2075–2081 (2006).

62. R. M. J. Deacon, J. N. P. Rawlins, T-maze alternation in the rodent. *Nat. Protoc.* **1**, 7–12 (2006).

63. J. Ng, S. Barral, C. De La Fuente Barrigon, G. Lignani, F. A. Erdem, R. Wallings, R. Privolizzi, G. Rossignoli, H. Alrashidi, S. Heasman, E. Meyer, A. Ngoh, S. Pope, R. Karda, D. Perocheau, J. Baruteau, N. Suff, J. Antinao Diaz, S. Schorge, J. Vowles, L. R. Marshall, S. A. Cowley, S. Sucic, M. Freissmuth, J. R. Counsell, R. Wade-Martins, S. J. R. Heales, A. A. Rahim, M. Bencze, S. N. Waddington, M. A. Kurian, Gene therapy restores dopamine transporter expression and ameliorates pathology in iPSC and mouse models of infantile parkinsonism. *Sci. Transl. Med.* **13**, eaaw1564 (2021).

64. J. I. Morgan, D. R. Cohen, J. L. Hempstead, T. Curran, *Science* **237**, 3213 (1987).

65. V. N. Barros, M. Mundim, L. T. Galindo, S. Bittencourt, M. Porcionatto, L. E. Mello, The pattern of c-Fos expression and its refractory period in the brain of rats and monkeys. *Front. Cell. Neurosci.* **9**, 72 (2015).

66. M. E. Greenberg, E. B. Ziff, Stimulation of 3T3 cells induces transcription of the c-fos proto-oncogene. *Nature* **311**, 433–438 (1984).

67. G. Tur, E. I. Georgieva, A. Gagete, G. López-Rodas, J. L. Rodríguez, L. Franco, Factor binding and chromatin modification in the promoter of murine Egr1 gene upon induction. *Cell. Mol. Life Sci.* **67**, 4065–4077 (2010).

68. A. L. Riffo-Campos, J. Castillo, G. Tur, P. González-Figueroa, E. I. Georgieva, J. L. Rodríguez, G. López-Rodas, M. I. Rodrigo, L. Franco, Nucleosome-specific, time-dependent changes in histone modifications during activation of the early growth response 1 (Egr1) gene. *J. Biol. Chem.* **290**, 197–208 (2015).

69. A. V. Tzingounis, R. A. Nicoll, Arc/Arg3.1: Linking gene expression to synaptic plasticity and memory. *Neuron* **52**, 403–407 (2006).

70. T. Kawashima, H. Okuno, M. Nonaka, A. Adachi-Morishima, N. Kyo, M. Okamura, S. Takemoto-Kimura, P. F. Worley, H. Bito, Synaptic activity-responsive element in the *Arc/Arg3.1* promoter essential for synapse-to-nucleus signaling in activated neurons. *Proc. Natl. Acad. Sci. U.S.A.* **106**, 316–321 (2009).

71. C. Prestigio, D. Ferrante, A. Marte, A. Romei, G. Lignani, F. Onofri, P. Valente, F. Benfenati, P. Baldelli, REST/NRSF drives homeostatic plasticity of inhibitory synapses in a target-dependent fashion. *eLife* **10**, e69058 (2021).

72. K. Ramamoorthi, R. Fropf, G. M. Belfort, H. L. Fitzmaurice, R. M. McKinney, R. L. Neve, T. Otto, Y. Lin, Npas4 regulates a transcriptional program in CA3 required for contextual memory formation. *Science* **334**, 1669–1675 (2011).

73. Y. Lin, B. L. Bloodgood, J. L. Hauser, A. D. Lapan, A. C. Koon, T.-K. Kim, L. S. Hu, A. N. Malik, M. E. Greenberg, Activity-dependent regulation of inhibitory synapse development by Npas4. *Nature* **455**, 1198–1204 (2008).

74. G. Lignani, P. Baldelli, V. Marra, Homeostatic plasticity in epilepsy. *Front. Cell. Neurosci.* **14**, 197 (2020).

75. R. Begum, Y. Bakiri, K. E. Volynski, D. M. Kullmann, Action potential broadening in a presynaptic channelopathy. *Nat. Commun.* **7**, 12102 (2016).

76. M. Okada, M. Kano, H. Matsuda, The degradation of the inwardly rectifying potassium channel, Kir2.1, depends on the expression level: Examination with fluorescent proteins. *Brain Res.* **1528**, 8–19 (2013).

77. E. Ambrosini, F. Sicca, M. S. Brignone, M. C. D'Adamo, C. Napolitano, I. Servettini, F. Moro, Y. Ruan, L. Guglielmi, S. Pieroni, G. Servillo, A. Lanciotti, G. Valvo, L. Catacuzzeno, F. Franciolini, P. Molinari, M. Marchese, A. Grottesi, R. Guerrini, F. M. Santorelli, S. Priori, M. Pessia, Genetically induced dysfunctions of Kir2.1 channels: Implications for short QT3 syndrome and autism-epilepsy phenotype. *Hum. Mol. Genet.* **23**, 4875–4886 (2014).
